# Transcriptomic Analysis of Tail Regeneration in the Lizard *Anolis carolinensis* Reveals Activation of Conserved Vertebrate Developmental and Repair Mechanisms

**DOI:** 10.1371/journal.pone.0105004

**Published:** 2014-08-20

**Authors:** Elizabeth D. Hutchins, Glenn J. Markov, Walter L. Eckalbar, Rajani M. George, Jesse M. King, Minami A. Tokuyama, Lauren A. Geiger, Nataliya Emmert, Michael J. Ammar, April N. Allen, Ashley L. Siniard, Jason J. Corneveaux, Rebecca E. Fisher, Juli Wade, Dale F. DeNardo, J. Alan Rawls, Matthew J. Huentelman, Jeanne Wilson-Rawls, Kenro Kusumi

**Affiliations:** 1 School of Life Sciences, Arizona State University, Tempe, Arizona, United States of America; 2 Neurogenomics Division, Translational Genomics Research Institute, Phoenix, Arizona, United States of America; 3 Department of Basic Medical Sciences, University of Arizona College of Medicine-Phoenix, Phoenix, Arizona, United States of America; 4 Departments of Psychology and Zoology, Program in Neuroscience, Michigan State University, East Lansing, Michigan, United States of America; Oxford Brookes University, United Kingdom

## Abstract

Lizards, which are amniote vertebrates like humans, are able to lose and regenerate a functional tail. Understanding the molecular basis of this process would advance regenerative approaches in amniotes, including humans. We have carried out the first transcriptomic analysis of tail regeneration in a lizard, the green anole *Anolis carolinensis*, which revealed 326 differentially expressed genes activating multiple developmental and repair mechanisms. Specifically, genes involved in wound response, hormonal regulation, musculoskeletal development, and the Wnt and MAPK/FGF pathways were differentially expressed along the regenerating tail axis. Furthermore, we identified 2 microRNA precursor families, 22 unclassified non-coding RNAs, and 3 novel protein-coding genes significantly enriched in the regenerating tail. However, high levels of progenitor/stem cell markers were not observed in any region of the regenerating tail. Furthermore, we observed multiple tissue-type specific clusters of proliferating cells along the regenerating tail, not localized to the tail tip. These findings predict a different mechanism of regeneration in the lizard than the blastema model described in the salamander and the zebrafish, which are anamniote vertebrates. Thus, lizard tail regrowth involves the activation of conserved developmental and wound response pathways, which are potential targets for regenerative medical therapies.

## Introduction

Regeneration of appendages in the adult is observed in a number of vertebrates, including in the lizard tail, the salamander limb and tail [1], and the zebrafish caudal fin [2]. Molecular and cellular analyses in these model organisms are beginning to reveal conserved versus divergent mechanisms for tissue regeneration [3]–[7], which impacts the translation of these findings to human therapies. Regeneration in newts is associated with proteins specific to urodele amphibians, casting doubt on the conservation of these regenerative pathways with other vertebrates [7]. In addition, muscle formation during limb regeneration differs between newts and the axolotl [8]. Mammals possess some neonatal regenerative capabilities, including mouse and human digit tip regeneration [9], [10] and heart regeneration in the mouse [11], but these processes are limited in the adult organism [12]. Lizards are capable of regrowing appendages, and as amniote vertebrates, are evolutionarily more closely related to humans than other models of regeneration, e.g., salamander and zebrafish. An examination of the genetic regulation of regeneration in an amniote model will advance our understanding of the conserved processes of regeneration in vertebrates, which is relevant to develop therapies in humans.

**Figure 1:**
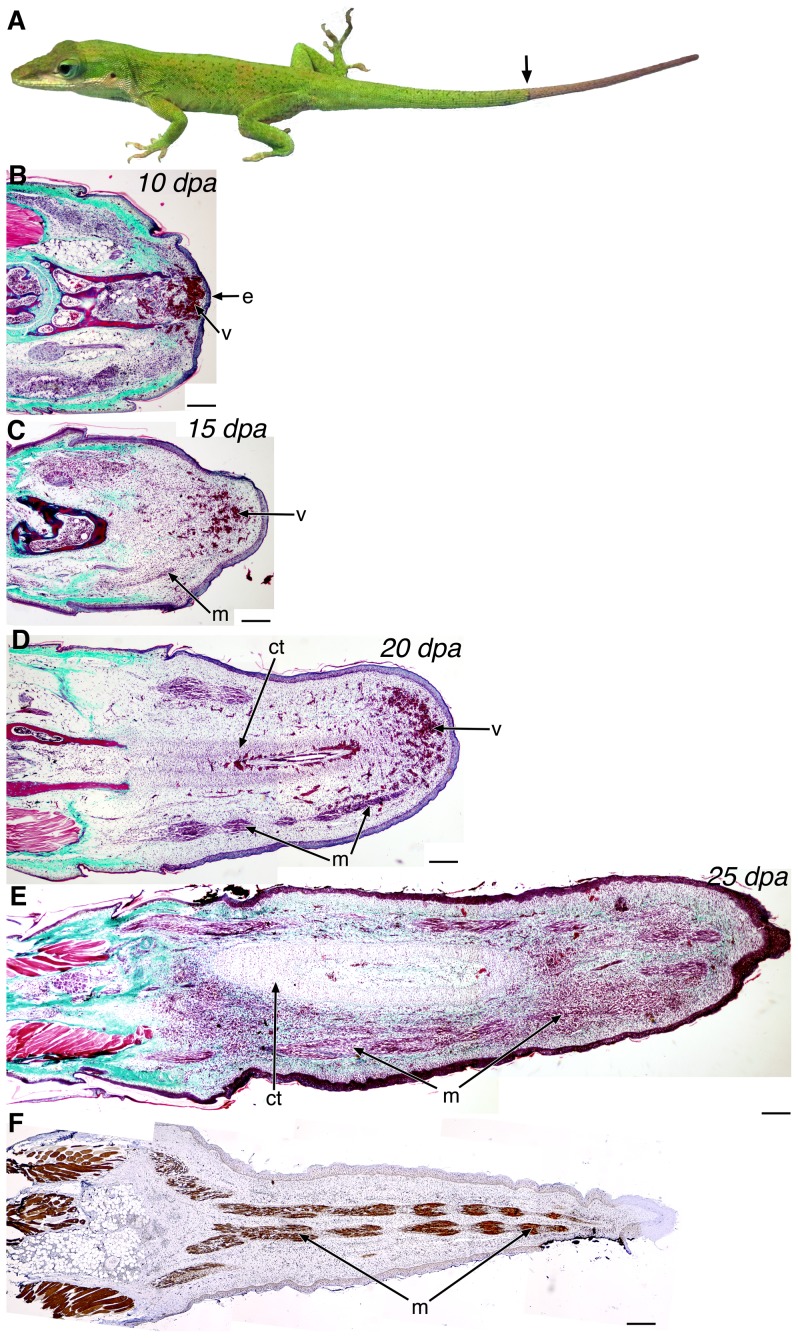
Overview of the stages of lizard tail regeneration. (*A*) *Anolis carolinensis* lizard with a regenerating tail (distal to arrow). (*B-E*) Histology of the 10 dpa (*B*), 15 dpa (*C*), 20 dpa (*D*), and 25 dpa (*E*) regenerating tail by Gomori's trichrome stain, with which connective tissues and collagen stain green-blue, muscle, keratin, and cytoplasm stain red, and nuclei are black. (*F*) Immunohistochemistry of myosin heavy chain in a 25 dpa regenerating tail using the MY-32 antibody. e, wound epithelium; v, blood vessels; m, muscle; ct, cartilaginous tissue. Composites: *B-F*. Scale bars in black: 200 µm.

In response to threats, lizards have evolved the ability to autotomize, or self-amputate, their tails and regenerate a replacement (Figure 1A) [13], [14]. The patterning and final structure of the lizard tail is quite distinct between embryonic development and the process of regeneration [15], [16]. Whereas the original tail skeleton and muscular groups are segmentally organized, reflecting embryonic patterning, the regenerated tail consists of a single unsegmented cartilaginous tube surrounded by unsegmented muscular bundles [15], [16]. In addition, the segmental organization of the spinal cord and dorsal root ganglia in the original tail are absent in the replacement, with regenerated axons extending along the length of the endoskeleton [17], [18]. While the regenerative process in lizards has been described previously [14]–[16], [19], [20], both the source of regenerating tissue and the cellular and molecular mechanisms that are activated during the regenerative process remain unclear. Dedifferentiation has been proposed to be a major source of proliferating cells in the anamniote salamander blastema model [21]. However, no clear evidence of dedifferentiation has been identified in tail regeneration in the lizard, an amniote vertebrate [14], [15], [19], [20]. A temporal-spatial gradient of tissue patterning and differentiation along the regenerating tail axis has been described [14], [19], [20].

The green anole lizard, *Anolis carolinensis*, is an emerging model organism, and has provided insights in the fields of evolution and development [22], [23], population genetics [24], [25], reproductive physiology [26], behavior [27], and functional morphology [28]. Large-scale gene expression analyses of biological processes such as tail regeneration in the green anole have previously been limited by a lack of genomic resources. However, the *A. carolinensis* genome was recently made available [29]. In addition, our group has generated a robust genome annotation based on 14 deep transcriptomes using both directional and non-directional RNA-Seq data from a diverse number of tissues [30]. These genomic resources provide a platform for transcriptome-wide analysis of the genes involved in regeneration in the green anole. Here we describe, to our knowledge, the first transcriptomic analysis of lizard tail regeneration.

## Materials and Methods

### Animals and collection of regenerating tail samples

Animals were collected and maintained in strict accordance with Protocol Number 12-1247R approved by the Institutional Animal Care and Use Committee at Arizona State University. Adult *A. carolinensis* lizards were purchased from Marcus Cantos Reptiles (Fort Myers, FL) or Charles D. Sullivan Co., Inc. (Nashville, TN). Animals were housed as previously described [15], [16]. Autotomy was induced by applying pressure to the tail until it was released. Animal health was monitored following autotomy. We collected 5 biological replicates of regenerating tail sections at 25 days post autotomy (dpa). Regenerating tails (n = 5) at 25 dpa were divided into five sections (approximately 1 mm each) for RNA-Seq analysis.

### RNA-Seq

RNA-Seq of the lizard embryos has been described previously [22]. Total RNA was isolated from tissue samples, including 25 dpa regenerating tail (n = 5) and satellite cells (n = 3; mirVana miRNA Isolation Kit total RNA protocol only, Ambion). The Ovation RNA-Seq kit (NuGEN) was used to synthesize double stranded cDNA. Paired-end sequencing libraries were then generated using manufacturer protocols and sequenced on an Illumina HiSeq 2000. For our analysis, 4 of the 5 regenerating tail replicates were multiplexed together and 2 of the 3 satellite cell replicates were multiplexed together.

### Bioinformatic analysis

RNA-Seq reads were trimmed to eliminate nucleotide bias where necessary. Trimmed reads were then mapped to the *A. carolinensis* genome [29] using Bowtie2.1.0 and TopHat2.0.8 with the ASU_Acar_v2.2.1 annotation revised from Eckalbar et al., 2013 [30] (Table S1). For Cuffdiff analysis, TopHat aligned reads were assembled using Cufflinks2.1.1 and genes with differential expression were identified using Cuffdiff2.1.1 with the following options: —upper-quartile-norm —multi-read-correct. Cuffdiff data were then imported into CummeRbund [31], [32]. For DESeq2 analysis, raw counts were generated from TopHat aligned reads using HTSeq and normalized for library size in DESeq2 [33]–[35]. In order to identify variant genes using DESeq2, normalized data were fitted to a negative binomial general linear model and adjusted for multiple testing using the Benjamini-Hochberg method, and a likelihood ratio test was performed. CummeRbund and DESeq2 are part of the Bioconductor set of software packages [36], which use the R statistical programming environment (http://www.R-project.org). P-values for Gene Ontology (GO) and Kyoto Encyclopedia of Genes and Genomes (KEGG) analysis of differentially expressed genes were generated using the Database for Annotation, Visualization, and Integrated Discovery (DAVID) functional analysis tool [37], [38]. Significant GO terms (p<0.05) were mapped with the REViGO online tool (http://revigo.irb.hr), which removes redundant GO terms and visualizes the semantic similarity of remaining terms [39]. For all heatmaps, genes were clustered by Jensen-Shannon divergence of the log10(FPKM+1) value.

### *A. carolinensis* genome annotation revision

An annotation of the *A. carolinensis* genome was reported using fourteen deep transcriptomes (ASU Acar v2.1) [30]. We further revised this annotation as follows: RNA-Seq data was assembled using the ABySS and Trans-ABySS pipeline [40]–[42]. Each of the 25 dpa regenerating tail sections was assembled individually in ABySS using every 5th kmer ranging from 26 bp to 96 bp. These assemblies were then combined using trans-ABySS to create a merged assembly with reduced redundancy. This merged assembly was then mapped to the genome using BLAT inside trans-ABySS. *De novo* assembled contigs were then filtered to require at least 90% coverage of the contig to the genome and to require at least one 25 bp gap. Seqclean was first used to remove Illumina adapters and any contaminants from the UniVec databases from the *de novo* assembled transcripts and the EST libraries. The cleaned *de novo* assembled transcripts from ABySS/Trans-ABySS were then assembled using the PASA reference genome guided assembly, and PASA alignment and assembly was executed using default parameters [43]–[46]. The PASA assemblies were then used to update the ASU Acar v2.1 annotations inside PASA to v2.2. The annotation was further updated to v2.2.1 with a subset of manual annotations.

### Isolation of satellite cells from *A. carolinensis*

Lizard satellite cell isolation was adapted from mammalian [47]–[49] and avian [50], [51] methods. Following euthanasia, large limb muscle groups were dissected in PBS and minced. Cells were separated by protease treatment and suspensions were initially plated to remove adherent fibroblasts and other debris. Satellite cells remaining in suspension were then collected and plated onto Matrigel-coated tissue culture plates in growth medium (Ham's F-10, 20% FBS, 100 µg/mL penicillin, 100 µg/mL streptomycin, 40 µg/mL gentamicin, 20 ng/mL bFGF) at 30°C in a 5% CO2 humidified chamber. While a number of conditions were tested, 30°C was the optimal temperature identified.

### Histological analysis

For paraffin sectioning, regenerated tails were fixed and embedded as described previously [15]. Embedded tails were sectioned into 20 µm sections using a CM1950UV Leica Cryostat and placed on HistoBond slides. Paraffin-embedded tissue sections were stained according to hematoxylin-eosin or Gomori's trichrome and mounted in Permount as described previously [15]. Hematoxylin stains nuclei and nucleoli blue and eosin stains cytoplasmic and extracellular matrix proteins pink/red, while hydrophobic cells such as adipocytes and myelin will remain clear. With Gomori's trichrome stain, connective tissues and collagen appear green-blue; muscle, keratin, and cytoplasm are red; and nuclei are black.

### Immunohistochemistry

Paraffin-embedded tissue sections were deparaffinized, rehydrated, and bathed in sodium citrate buffer (pH 6.0). Cells were fixed in 100% methanol. Tissue sections and cells were stained using the Histostain-SP Broad Spectrum kit (Invitrogen) as follows: Tissue sections and cells were blocked in serum, incubated with primary antibody (MY-32, Sigma Aldrich, MFCD00145920; PCNA, Santa Cruz Biotechnology, sc-7907; MCM2, Abcam, ab4461) incubated with secondary antibody, and incubated with HRP-strepavidin complex, with blocking and antibody incubations at 37°C. Tissue sections and cells were counterstained with hematoxylin and mounted in Permount (Fisher Scientific).

### Immunofluorescence

Cells were fixed in 100% methanol, blocked in serum, incubated with PAX7 antibody (Developmental Studies Hybridoma Bank), and incubated with secondary antibody, with blocking and antibody incubations at 37°C. Slides were then counterstained with DAPI.

### Data Access

RNA-Seq data for the lizard embryo samples, which have been previously reported [22], are deposited in at the National Center for Biotechnology Information (NCBI), under BioProject PRJNA149661. RNA-Seq data for the lizard tail regeneration and satellite cell samples are deposited under BioProject PRJNA253971.

## Results

### Histology of early regenerative stages

Progressively increasing tissue patterning and differentiation are evident in the early regenerative stages of the lizard tail. The first 10 days are characterized by wound healing (0–10 days post autotomy (dpa); Figure 1B). By 10 dpa, a wound epithelium has formed over the autotomized stump and blood vessels have formed immediately below. There was no appreciable outgrowth at this stage. Outgrowth begins after the wound epithelium forms and is characterized by early growth of the ependyma from the spinal cord into the surrounding mesenchymal tissue (10–15 dpa). By 15 dpa, there was noticeable outgrowth of highly vascularized tissue and myofibers began to form (Figure 1C). With continued tail outgrowth, the central cartilage tube and surrounding skeletal muscle began to differentiate (15–20 dpa; Figure 1D). Note that the tip of the tail remains vascular (10–20 dpa, Figure 1B-D). By 25 dpa, further lengthening of the regenerating tail was observed, along with formation of muscle and cartilage surrounding the ependymal core (Figure 1E). Further outgrowth with continued tissue differentiation is evident post-25 dpa, and there is no significant outgrowth after 60 dpa [15]. In fact, by 25 dpa, myosin heavy chain (MHC) positive skeletal muscle was present along the length of the developing tail, except at the very distal tip (Figure 1F). Spatially, there is an increase in patterning and differentiation along the regenerating tail was observed at early outgrowth stages (e.g., 15–25 dpa, Figure 1C-E), with differences in tissue organization particularly evident along the proximal-distal axis.

### Sequencing and differential expression testing of regenerating tail transcripts

**Figure 2:**
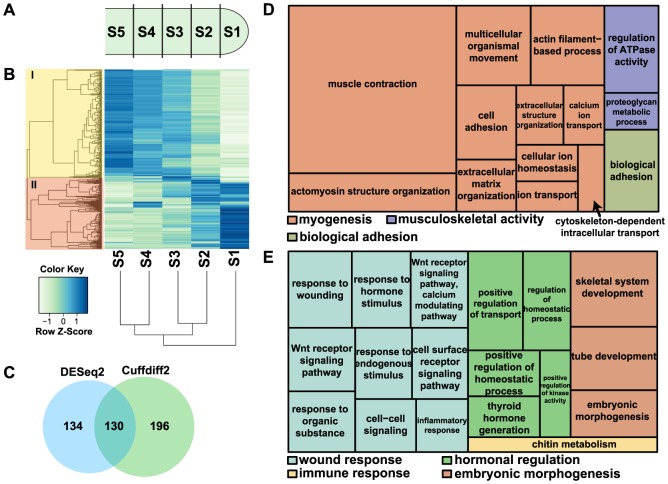
Transcriptomic analysis of gene expression in the 25 dpa regenerating lizard tail. (*A*) 25 dpa regenerated tail tissue was divided into five equal sized segments (S1-S5) with S1 representing the most distal regenerating tip, and total RNA was extracted for RNA-Seq analysis. (*B*) A heatmap showing 326 genes that were differentially expressed, i.e., displayed significant differences between any two segments in the regenerating tail as determined by Cuffdiff (p<0.05). Genes were clustered by Jensen-Shannon divergence of the log10(FPKM+1) value into two major groups, as shown in the dendrogram on the left. 129 genes displayed increased expression distally towards the tail tip (Cluster II) while 197 displayed increased expression proximally (Cluster I). This clustering also demonstrated that the distal-most regenerating tail tip (S1) was the outlier among these samples. (*C*) Venn diagram of differentially expressed genes identified by DESeq2 and Cuffdiff2. (*D-E*) A treemap overview of differentially expressed genes in (*D*) Cluster I and (*E*) Cluster II based on representative Gene Ontology Biological Processes. The relative sizes of the treemap boxes are based on the |log10(p-value)| of the respective GO term. Related terms are visualized with the same color, with the representative category for each color group denoted in the legend.

To identify differentially expressed genes along the proximal-distal axis of regenerating tails, we carried out RNA-Seq analysis on five tails at 25 dpa (Table S2). Tails were sectioned into five segments of equal length (Figure 2A). RNA-Seq analysis identified 326 differentially expressed genes with p<0.05 after correcting for multiple testing using Cuffdiff2 [31], [52], 302 of which have mammalian orthologs (Figure 2B; Table S3). Data were also analyzed by DESeq2 [33], [34], which yielded 264 differentially expressed genes, 252 of which have mammalian orthologs (Figure 2C; Table S4). These Cuffdiff2 differentially expressed genes clustered into two major groups, representing genes elevated towards the proximal base (Cluster I, Figure 2B) or the distal tip (Cluster II, Figure 2B).

### Differential expression of genes involved in developmental and repair mechanisms in the regenerating tail

Our RNA-Seq analysis identified Gene Ontology (GO) groups associated with the differentiation of tissues present in the proximal regenerating tail, predominantly those that are specific to skeletal muscle (Figure 2D; Figure S1A; Table 1; Table S5). Sarcomeric proteins, including myosin heavy chains and actinins, were elevated in the proximal tail. This pattern of expression was validated by the presence of myosin heavy chain positive muscle fibers (Figure 1F). Myogenic regulatory factors associated with muscle growth and repair were also elevated in the proximal tail. These include the transcription factors *pax7*, mohawk (*mkx*), and *tcf15*, which are expressed in myogenic stem/progenitor cells [53]–[55], NFATc1, which regulates muscle hypertrophy [56], and the TGFβ family member myostatin (*mstn*), which modulates muscle mass [57] (*Anolis* Gene Nomenclature Committee standards used for gene symbols; [58]). Also, the MADS box factor *mef2c*, and the myogenic regulatory factor *myod1*, which synergize to activate muscle specific gene transcription, were elevated [59]. As growth and repair of skeletal muscle in vertebrates normally relies on the expansion and differentiation of muscle-specific progenitor cells, the enrichment for genes associated with the regulation of this population predicts a similar mechanism of muscle growth and repair occurring in a zone of active regeneration. Furthermore, the increase in *mkx* transcription raises the possibility of a coordinated growth between tendons and muscle in the regenerating tail, given that the orthologous gene is required for growth and repair in mammals [60].

**Table 1:** Selected Genes Ontology categories represented along the regenerating tail axis.

	Category	GO Term	Description	Count	P-value	Genes
**Cluster I**						
	**myogenesis**	GO:0006936	muscle contraction	30	6.63E-29	*mybpc2, tnnc2, tnnc1, myl3, mybpc1, mybpc3, myl1, pgam2, myot, des, myom2, myl6b, myom1, chrna1, scn5a, dtna, kcnma1, actc1, acta1, actn2, myh6, tnni2, trdn, tnnt3, tnnt1, ryr1, stbd1, chrne, casq2, chrng*
		GO:0007517	muscle organ development	28	3.44E-22	*mef2c, myod1, myl2, tnnc1, myl3, mybpc3, myl1, trim72, speg, myl6b, pax7, obsl1, mkx, mkl2, chrna1, actc1, acta1, mstn, mylpf, myh6, csrp3, flnb, murc, neb, xirp1, itga7, vgll2, tcf15*
		GO:0007519	skeletal muscle tissue development	9	3.73E-07	*myod1, acta1, myl3, myl6b, pax7, mylpf, vgll2, chrna1, csrp3*
		GO:0042692	muscle cell differentiation	11	4.86E-07	*myod1, actc1, acta1, xirp1, myl2, speg, lgals1, obsl1, myh6, mkl2, chrna1*
		GO:0050881	musculoskeletal movement	6	1.14E-06	*tnnt3, tnnt1, tnnc2, tnnc1, chrna1, tnni2*
		GO:0030029	actin filament-based process	14	1.28E-06	*actc1, tnxb, myl2, acta1, myl1, pdlim3, myh6, gas7, flnb, xirp1, xirp2, myl6b, limch1, obsl1*
		GO:0007155	cell adhesion	21	3.41E-05	*hapln1, tnxb, mybpc2, clstn2, egfl6, lpp, mybpc1, col22a1, mybpc3, col28a1, mgp, actn2, col2a1, actn3, ecm2, col9a1, itga7, acan, susd5, col11a2, thbs4*
		GO:0001501	skeletal system development	12	4.79E-04	*bmp3, col9a1, col9a2, tbx15, lect1, clec3a, pax7, acan, mgp, col2a1, col11a2, tcf15*
		GO:0030198	extracellular matrix organization	7	7.29E-04	*csgalnact1, tnxb, adamts20, acan, col2a1, col11a2, ecm2*
		GO:0030705	cytoskeleton-dependent intracellular transport	4	0.0166	*actc1, myl6b, myl1, myh6*
		GO:0006873	cellular ion homeostasis	11	0.0055	*kcnma1, jph2, xirp1, pygm, atp2a1, ryr1, chrna1, chrne, csrp3, sypl2, chrng*
	**chondrogenesis**	GO:0051216	cartilage development	8	1.10E-05	*bmp3, col9a1, lect1, pax7, acan, mgp, col2a1, col11a2*
		GO:0002062	chondrocyte differentiation	4	7.90E-04	*col9a1, acan, col2a1, col11a2*
		GO:0001502	cartilage condensation	3	0.0162	*acan, mgp, col2a1*
	**musculoskeletal activity**	GO:0043462	regulation of ATPase activity	5	1.82E-05	*tnnt3, myl3, tnnc1, mybpc3, myh6*
		GO:0006029	proteoglycan metabolic process	4	0.0099	*csgalnact1, lect1, acan, col2a1*
	**biological adhesion**	GO:0022610	biological adhesion	21	3.48E-05	*hapln1, tnxb, mybpc2, clstn2, egfl6, lpp, mybpc1, col22a1, mybpc3, col28a1, mgp, actn2, col2a1, actn3, ecm2, col9a1, itga7, acan, susd5, col11a2, thbs4*
**Cluster II**						
	**wound response**	GO:0009611	response to wounding	10	0.0040	*pcsk1, scube1, pdgfra, pla2g7, entpd1, ptx3, mdk, igfbp4, f2r, spp1*
		GO:0009725	response to hormone stimulus	8	0.0059	*cga, pcsk1, krt19, tnfrsf11b, bsg, th, pdgfra, spp1*
		GO:0007223	Wnt receptor signaling pathway, calcium modulating pathway	3	0.0067	*wnt5a, wnt16, ror2*
		GO:0016055	Wnt receptor signaling pathway	5	0.0079	*dkk2, wnt5a, wnt16, ror2, wif1*
		GO:0007166	cell surface receptor signaling pathway	20	0.0106	*wnt5a, cga, edn3, fgfr4, il1r1, wnt16, gpr158, bsg, maml2, ptpn22, thy1, dkk2, ednra, or5v1, pdgfra, ror2, wif1, pdgfc, entpd1, f2r*
		GO:0010033	response to organic substance	11	0.0098	*ednra, cga, pcsk1, krt19, il1r1, tnfrsf11b, bsg, th, pdgfra, f2r, spp1*
		GO:0006954	inflammatory response	6	0.0433	*scube1, pla2g7, ptx3, igfbp4, f2r, spp1*
	**hormonal regulation**	GO:0051050	positive regulation of transport	7	0.0020	*ednra, edn3, pcsk1, rab8b, ptx3, f2r, thy1*
		GO:0032844	regulation of homeostatic process	5	0.0046	*ednra, tnfrsf11b, f2r, spp1, thy1*
		GO:0006590	thyroid hormone generation	2	0.0350	*cga, dio2*
	**embryonic morphogenesis**	GO:0001501	skeletal system development	9	5.81E-04	*wnt5a, tnfrsf11b, pdgfra, ror2, mepe, cbfb, igfbp4, spp1, twist1*
		GO:0035295	tube development	7	0.0019	*wnt5a, ednra, fgfr4, sall1, pdgfra, ptk7, twist1*
		GO:0048598	embryonic morphogenesis	7	0.0096	*wnt5a, sall4, th, ptk7, ror2, twist1, ptprq*
	**immune response**	GO:0006030	chitin metabolic process	2	0.0407	*chi3l1, chit1*

Our transcriptome analysis identified multiple genetic pathways activated towards the tip of the regenerating tail. Genes differentially elevated at the tip were enriched for GO categories related to i.) wound response, ii.) hormonal regulation, and iii.) embryonic morphogenesis (Figure 2E; Figure S1B; Table 1; Table S6). Wound and inflammatory response genes elevated in the distal regenerating tail include *igfbp4*, *mdk*, *ptx3*, and *pdgfra*. Mouse *Ptx3* is required for fungal resistance [61], and *Mdk* plays a role in angiogenesis [62]. Hormonal and homeostatic regulation genes included those involved in thyroid hormone generation, such as *cga* and *dio2*. Thyroid hormone plays a critical role in neuromuscular growth, both during normal development and in repair after injury. *Dio2* has been shown to co-regulate myogenesis and muscle regeneration in the mouse [63]. In the rat model, triiodothyronine (T3) treatment after sciatic nerve injury has been shown to enhance reinnervation of muscles [64]. In the *Xenopus laevis* tadpole, thyroid hormone is critical for limb development during metamorphosis, where limb muscle growth, innervation of the limb, cartilage growth, and skin development are all thyroid hormone-dependent [65]. Genes involved in homeostatic regulation and vascular development include *ednra* and *edn3*, which are members of the endothelin family and regulate vasoconstriction and cell proliferation [66], the thrombin receptor *f2r*, which promotes vascular development by negatively regulating hematopoietic differentiation of mouse embryonic stem cells [67], and *thy1*, which is a marker of angiogenesis [68]. The *wnt5a* ligand and its receptor, *ror2*, were both significantly expressed at the tip, indicating non-canonical Wnt signaling, which can promote chondrogenesis [69], [70]. Skeletal system development genes elevated in the regenerating tail include the basic helix-loop-helix transcription factor *twist1*, which regulates a number of pathways, including FGF, by chromatin modification via histone acetyltransferases [71].

**Figure 3:**
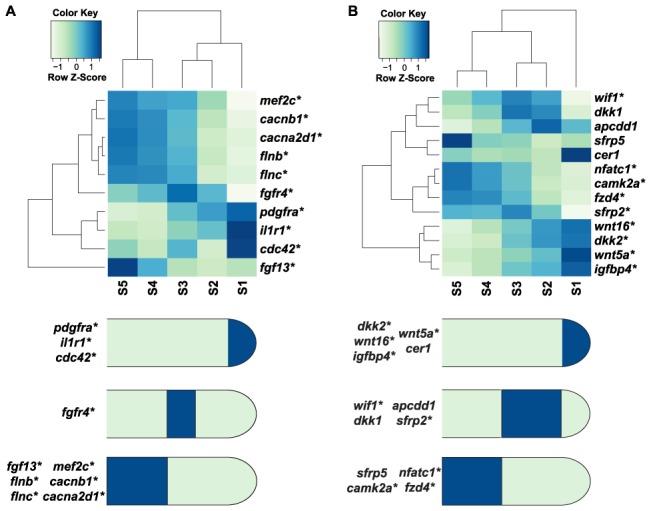
MAPK/FGF and Wnt pathway genes differentially expressed in the 25 dpa regenerating lizard tail. (*A, B*) Based on RNA-Seq analysis described in Figure 2, the heatmaps show the 10 MAPK/FGF pathway genes (*A*), or 9 Wnt pathway genes (*B*) defined by KEGG, that were differentially expressed, i.e., displayed significant differences between any two segments in the regenerating tail as determined by Cuffdiff2 (p<0.05), along with previously identified Wnt inhibitors. A diagram summarizing the tail segment(s) with highest expression level for each MAPK/FGF (*A*) or Wnt (*B*) pathway gene is also shown. Differentially expressed genes are denoted with an asterisk.

Differentially expressed genes analyzed for Kyoto Encyclopedia of Genes and Genomes (KEGG) categories (p<0.05) identified axon guidance and neural development genes, including slit homolog 2 (*slit2*), actin binding LIM protein family member 2 (*ablim2*), and netrin receptor unc-5 homolog C (*unc5c*) (Table 1; Table S7). KEGG groups enriched in the regenerating tail also include the Wnt and MAPK/FGF signaling pathways. FGF signaling plays a key role in developmental patterning, proliferation, and differentiation [72]. Differentially expressed MAPK/FGF pathway genes at the tail tip include *pdgfra*, *il1r1*, and *cdc42* while *mef2c*, *cacnb1*, *cacna2d1*, *flnb*, *flnc*, and *fgfr13* are elevated at the proximal region of the regenerating tail (Figure 3A). A number of recent reports from mouse digit tip and salamander limb regeneration identified Wnt pathway involvement [3], [4], [10]. Wnt signaling promotes the differentiation of embryonic stem cells as well as cells from skeletal muscle, osteogenic, and cardiogenic lineages [73]. The tip to the middle regions of the regenerating tail are enriched with Wnt inhibitors, including *dkk2*, *igfbp4*, *wif1*, and *sgfrp2* (Figure 3B). The expression of soluble Wnt inhibitors from this region could create a proximal-distal gradient of Wnt signaling that is necessary to maintain the actively growing zone of the regenerating tail in a proliferative, undifferentiated state.

### Novel and uncharacterized transcripts in the regenerating tail

We sought to characterize the 22 differentially expressed genes, representing 29 transcript isoforms, without clear orthology, i.e., BLAST alignment scores against the nonredundant protein database were either E≥1.0, identity was ≤50%, or no match was identified. These transcripts could potentially be protein-coding genes specific to squamate reptiles, either novel or highly divergent within the squamate lineage, or could represent noncoding RNA species. Transcripts were queried against the protein family (Pfam) [74] and RNA family (Rfam) [75] databases, and coding potential was evaluated using the Coding-Non-Coding Index (CNCI; [76]), which evaluates coding potential by profiling adjoining trinucleotide sequences (Table 2). Four transcripts were identified as retrotransposons, including the gag-pol polyprotein and RNA-directed DNA polymerase from mobile element jockey-like, which are enriched in the proximal regenerating tail. Of the remaining transcripts, 3 were predicted as protein-coding and 22 were characterized as non-coding by the CNCI. The protein-coding gene ASU_Acar_G.15880, which is differentially expressed in the proximal regenerating tail, has a DUF4585 (domain of unknown function) domain, and orthologous genes found in the king cobra (*Ophiophagus hannah*; GenBank: ETE69491.1) genome, the green sea turtle (*Chelonia mydas*; GenBank: EMP32806.1; NCBI: XP_007063098.1) genome, and the axolotl (*Ambyostoma mexicanum*) transcriptome. The 2 remaining protein-coding transcripts were not matched to any known domains in the Pfam database. Of the 22 non-coding transcripts, we identified 2 differentially expressed genes in the proximal tail categorized within the miRNA precursor families miR-133 and miR-324. miR-133 acts in a negative feedback loop with serum response factor (SRF) to promote myoblast differentiation *in vitro*, and suppresses BMP2-induced osteogenesis by targeting *Runx2* [77], [78]. The remaining 20 non-coding transcripts represent potential modulators of genes down-regulated in regeneration. In summary, these unidentified transcripts represent novel protein-coding genes, long non-coding RNAs, and microRNAs that may regulate the regenerative process in concert with identified genes and signaling pathways.

**Table 2:** Novel and uncharacterized transcripts in the regenerating tail.

	Gene ID	Transcript ID	CNCI score	CNCI classification	Length (bp)	Longest ORF (bp)	Domain/Homology	Highest Section
**Predicted RNA only**								
	ASU_Acar_G.1063	ASU_Acar_T.1063.1	0.0	non-coding	216	213	lncRNA	S1
	ASU_Acar_G.14483	ASU_Acar_T.14483.1	−0.0029	non-coding	698	153	lncRNA	S4
	ASU_Acar_G.14483	ASU_Acar_T.14483.2	−0.0784	non-coding	1256	195	lncRNA	S4
	ASU_Acar_G.14483	ASU_Acar_T.14483.5	−0.0029	non-coding	712	153	lncRNA	S2
	ASU_Acar_G.14483	ASU_Acar_T.14483.7	−0.0029	non-coding	1430	195	lncRNA	-
	ASU_Acar_G.17546	ASU_Acar_T.17546.1	−0.0390	non-coding	225	222	lncRNA	S1
	ASU_Acar_G.17964	ASU_Acar_T.17964.1	−0.1550	non-coding	219	123	lncRNA	S4
	ASU_Acar_G.5235	ASU_Acar_T.5235.1	0.0	non-coding	216	213	lncRNA	S3
	ASU_Acar_G.7180	ASU_Acar_T.7180.1	−0.0038	non-coding	243	240	lncRNA	S5
	ASU_Acar_G.8849	ASU_Acar_T.8849.1	−0.0532	non-coding	291	288	lncRNA	S4
	ASU_Acar_G.8944	ASU_Acar_T.8944.1	−0.2007	non-coding	279	276	lncRNA	S1
	ASU_Acar_G.20175	ASU_Acar_T.20175.1	−0.0204	non-coding	261	258	lncRNA	S1
	ASU_Acar_G.1922	ASU_Acar_T.1922.1	−0.0114	non-coding	2286	213	mir-133	S5
	ASU_Acar_G.19355	ASU_Acar_T.19355.1	−0.0064	non-coding	2549	219	mir-324	S5
	ASU_Acar_G.10886	ASU_Acar_T.10886.1	−0.1770	non-coding	637	384	ncRNA	S1
	ASU_Acar_G.13829	ASU_Acar_T.13829.1	−0.0563	non-coding	189	186	ncRNA	S4
	ASU_Acar_G.14483	ASU_Acar_T.14483.4	0.0	non-coding	183	180	ncRNA	S3
	ASU_Acar_G.14483	ASU_Acar_T.14483.6	−0.0073	non-coding	459	456	ncRNA	S4
	ASU_Acar_G.14791	ASU_Acar_T.14791.1	0.0	non-coding	199	114	ncRNA	S1
	ASU_Acar_G.1721	ASU_Acar_T.1721.1	−0.0170	non-coding	192	189	ncRNA	S2
	ASU_Acar_G.2935	ASU_Acar_T.2935.1	0.0000	non-coding	195	192	ncRNA	S1
	ASU_Acar_G.3586	ASU_Acar_T.3586.1	0.0000	non-coding	195	192	ncRNA	S1
**Protein Coding - Not Described**								
	ASU_Acar_G.15880	ASU_Acar_T.15880.1	0.1481	coding	14705	4992	DUF4585	S2
	ASU_Acar_G.14483	ASU_Acar_T.14483.3	0.0510	coding	3395	2766	unknown	S5
	ASU_Acar_G.19198	ASU_Acar_T.19198.1	0.0293	coding	264	261	unknown	S1
**Retrotransposons**								
	ASU_Acar_G.14133	ASU_Acar_T.14133.1	0.2166	coding	3618	3615	gag-pol polyprotein	S5
	ASU_Acar_G.591	ASU_Acar_T.591.1	0.1336	coding	762	759	gag-pol polyprotein	S3
	ASU_Acar_G.591	ASU_Acar_T.591.2	−0.0102	non-coding	198	195	gag-pol polyprotein	S5
	ASU_Acar_G.4168	ASU_Acar_T.4168.1	0.0918	coding	2010	1863	rna-directed dna polymerase from mobile element jockey-like	S5

### Comparison of regenerating tail with stem/progenitor cells and developing embryo

**Figure 4:**
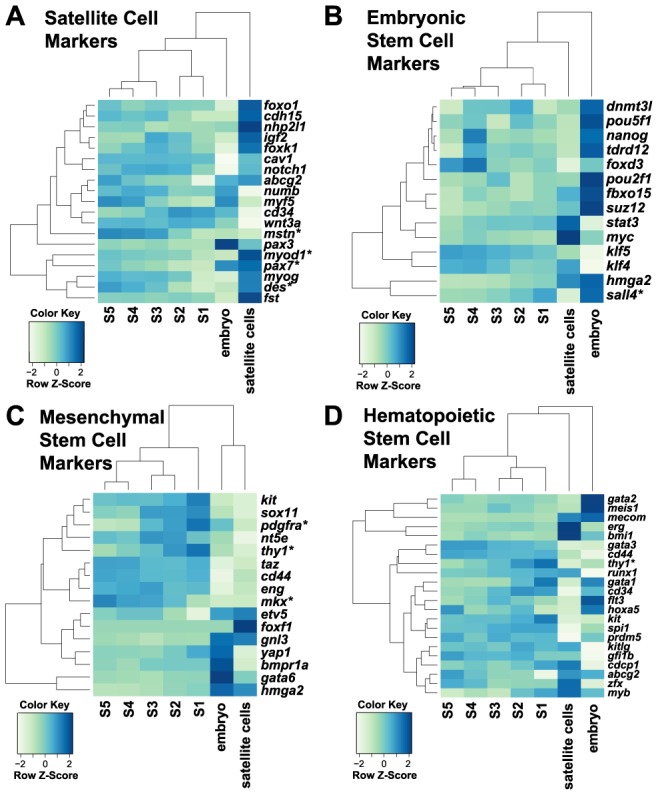
The 25 dpa regenerating tail has limited relative expression of stem cell markers. (*A-D*) Heatmap showing gene expression of satellite cell (*A*) and embryonic (*B*), mesenchymal (*C*), and hematopoietic stem cell markers in lizard embryos (n = 2), satellite cells (n = 3), and 25 dpa regenerating tail sections (n = 5). Differentially expressed genes along the regenerating tail axis are denoted with an asterisk.

Tissue regeneration in the lizard tail requires a source of cells; these could be tissue-specific oligopotent or progenitor stem cells, as in mammalian tissue repair, since there is no evidence of dedifferentiation in the lizard as observed in the salamander [14], [15], [19], [20]. We analyzed the regenerated tail in comparison with lizard embryos and satellite cells; both are highly enriched for stem cell populations (Figure S2). We profiled the transcriptome of lizard embryos at the 28–38 somite pair stages [79]. At this stage, the embryo contains paraxial mesoderm, a multipotent cell source for skeletal muscle, cartilage, bone, and tendon. Satellite cells capable of differentiating into skeletal muscle in response to injury serve as progenitor/stem cells for adult muscle repair in mammals [80]. We isolated a PAX7 positive cell population from adult lizard skeletal muscle that was morphologically comparable to mouse satellite cells (Figure S3A). These cells differentiated into multinucleated, MHC positive myotubes (Figure S3B), and express many of the same lineage-specific genes (Figure 4A, Figure S3C-F). The lizard embryos and satellite cells each possess distinct gene expression signatures based on gene markers for mouse and human embryonic, hematopoietic, and mesenchymal stem cells and satellite cells. In contrast, these genes are expressed at low levels without a distinct proximal-distal pattern in the regenerating tail (Figure 4A-D). These data predict a role for stem cells distributed throughout the regenerating tail, instead of being localized to the distal tip with a distal-to-proximal gradient of differentiation within the tail. While there are genes elevated in the regenerating tail relative to the embryo and satellite cells, genes elevated in the regenerating tail tip are primarily involved in the formation of tissues specific to the tail such as keratin-associated beta protein, and genes elevated in the proximal regenerating tail are primarily involved in tissue differentiation (Figure S2, Tables S8-S9). The lack of intensity in the signal compared to the embryo and satellite cells could be due to stem cells comprising only a minority population in the regenerating tail.

### Distributed pattern of cell proliferation in the regenerating tail

**Figure 5:**
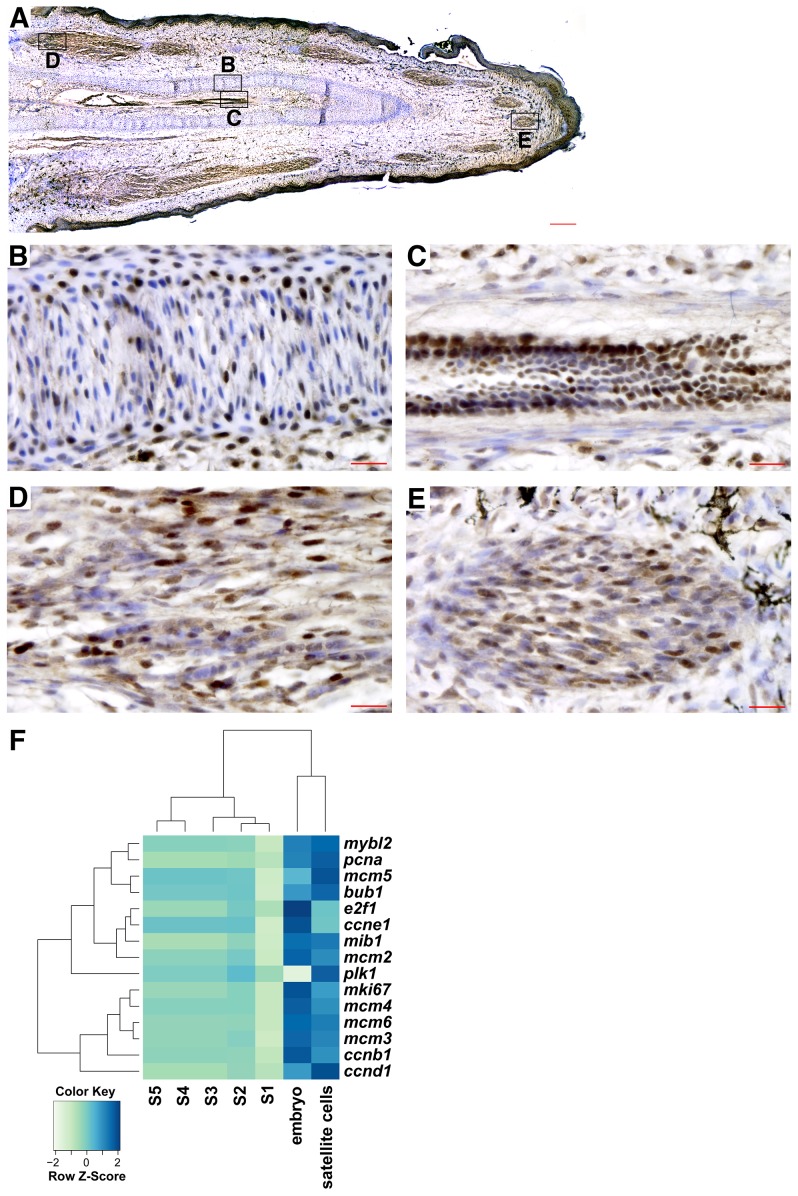
Histological and RNA-Seq analysis of proliferation in the 25 dpa regenerating tail. (*A-E*) MCM2 immunohistochemistry of the 25 dpa regenerating tail (brown nuclei), counterstained with hematoxylin (blue nuclei). (*A*) MCM2 is expressed throughout the regenerating tail, indicating a lack of a single proliferative zone. The condensing cartilage tube (*B*), ependymal core (*C*), developing muscles near the proximal base (*D*) and tip (*E*) of the regenerating tail are shown. (*F*) A heatmap showing gene expression of proliferative markers in the regenerating tail, the embryos, and satellite cells. DE genes along the regenerating tail axis are denoted with an asterisk. Composites: *A*. Scale bars in red: 200 µm (*A*) and 20 µm (*B-E*).

Proliferation and specification of progenitor cells is required for growth of the regenerating tail. While the regenerating tail did not express high levels of stem cell factors, selected progenitor/stem cell markers still displayed differential expression along the proximal-distal axis (Figure 4A-D). These genes included platelet-derived growth factor receptor *pdgfra*, which is expressed in subtypes of mesenchymal progenitor cells involved in muscle repair [81]. In addition, genes elevated in the tail tip include the *kit* ligand and *sox11* transcription factor, and genes elevated towards the proximal tail included the previously discussed transcription factor *mkx*. To visualize the pattern of proliferating cells within the regenerating tail, we analyzed the distribution of minichromosome maintenance complex component 3 (MCM2) in the regenerating tail (Figure 5A-E). MCM2 positive cells are observed in distributed, discrete regions in the regenerating tail, including the condensing cartilage tube and ependymal core (Figure 5B-C) and in developing muscle (Figure 5D-E). A second marker of proliferation, proliferating cell nuclear antigen (PCNA), showed a similar pattern of expression, confirming that proliferating cells are distributed throughout the regenerating tail in comparison to low levels of proliferating cells in the original tail (Figure S4; Figure S5). This pattern of proliferation is corroborated by RNA-Seq analysis of proliferation markers along the regenerating tail (Figure 5F). No segment along the proximal-distal axis of the regenerating tail demonstrated elevated expression of these markers, indicating that there is no single growth zone.

## Discussion

While transcriptomic analysis has been carried out in anamniote regenerative models, including the zebrafish tail, the newt limb, and the axolotl limb [3], [4], [6], [7], the genetic profile of pathways activated in regeneration of amniote appendages has not been described. Through transcriptomic analysis of lizard tail regeneration, we have identified that genes in pathways involved in developmental processes, including myogenesis, chondrogenesis, and neurogenesis, as well as adult processes, such as wound and immune responses, and are differentially expressed along the regenerating tail axis. The Wnt pathway was significantly enriched along the regenerating lizard tail axis, and activation of this pathway has also been noted in salamander tail tip and mouse digit tip regeneration [3], [4], [10]. Specifically, the Wnt pathway members *wnt5a* and *wif1* are differentially expressed in lizard as well as the salamander [3], [4]. The activation of Wnt signaling in two amniote lineages, mammals and squamate reptiles, as well as urodele amphibians supports a role for this pathway in regeneration that is conserved among tetrapod vertebrates. Transcriptomic analysis also revealed that genes involved in thyroid hormone generation (GO category GO:0006590; Table 1; Table S6) were differentially expressed, suggesting a regulatory connection between regeneration of the lizard tail and musculoskeletal transformations during amphibian metamorphosis. The lizard *dio2* gene is the ortholog of deiodinase, iodothyronine, type I, which in mammals converts thyroxine prohormone (T4) to bioactive 3,3',5-triiodothyronine (T3) [82]. In *Xenopus laevis*, T3 is the key signal for the process of metamorphosis from tadpole to adult frog [83]. Many of the changes associated with metamorphosis are also observed in the remodeling of the tail stump and outgrowth of the lizard tail. The lizard *cga* gene is the ortholog of chorionic gonadotropin, alpha chain, which encodes the alpha chain of thyroid-stimulating hormone and other key hormones [84]. During tadpole metamorphosis, both thyroid hormone (TH) and thyroid-stimulating hormone (TSH) rise, despite the normal expectation that TH would down-regulate TSH [85]. Changes in TH regulation of TSH may also be altered in regeneration, which has not been studied in the lizard. It is possible that among the amniotes, the lizard retains genetic pathways associated with thyroid hormone regulation of metamorphosis in amphibian vertebrates. Similarly, we previously identified conserved features in Notch pathway regulation of lizard and amphibian development, specifically a gradient of *hes6* expression in the presomitic mesoderm that was not observed in other amniote vertebrates and presumably lost [79]. Our transcriptomic analysis has highlighted the activation of multiple genetic pathways, sharing genes that have been identified as regulating development or wound response processes in other vertebrate model systems.

Developmental systems display different patterns of tissue outgrowth. For example, some tissues are formed from patterning from a localized region of a single multipotent cell type, such as the axial elongation of the trunk through production of somites from the presomitic mesoderm [86]. Other tissues are formed from the distributed growth of distinct cell types, such as the development of the eye from neural crest, mesenchymal, and placodal ectodermal tissue [87]. The regeneration of the amphibian limb involves a region of highly proliferative cells adjacent to the wound epithelium, the blastema, with tissues differentiating as they grow more distant from the blastema. However, regeneration of the lizard tail appears to follow a more distributed model. Stem cell markers and PCNA and MCM2 positive cells are not highly elevated in any particular region of the regenerating tail, suggesting multiple foci of regenerative growth. This contrasts with PNCA and MCM2 immunostaining of developmental and regenerative growth zone models such as skin appendage formation [88], liver development [89], neuronal regeneration in the newt [90], and the regenerative blastema [91], which all contain localized regions of proliferative growth. Skeletal muscle and cartilage differentiation occurs along the length of the regenerating tail during outgrowth; it is not limited to the most proximal regions. Furthermore, the distal tip region of the regenerating tail is highly vascular, unlike a blastema, which is avascular [92]. These data suggest that the blastema model of anamniote limb regeneration does not accurately reflect the regenerative process in tail regeneration of the lizard, an amniote vertebrate.

Regeneration requires a cellular source for tissue growth. Satellite cells, which reside along mature myofibers in adult skeletal muscle, have been studied extensively for their involvement in muscle growth and regeneration in mammals and other vertebrates [53], [55], [60], [80], [93]. For example, regeneration of skeletal muscle in the axolotl limb involves recruitment of satellite cells from muscle [8]. Satellite cells could contribute to the regeneration of skeletal muscle, and potentially other tissues, in the lizard tail. Mammalian satellite cells *in vivo* are limited to muscle, but *in vitro* with the addition of exogenous BMPs, they can be induced to differentiate into cartilage as well [80], [81]. High expression levels of BMP genes in lizard satellite cells could be associated with greater differentiation potential, and further studies will help to uncover the plasticity of this progenitor cell type.

In summary, we have identified a coordinated program of regeneration in the green anole lizard that involves both recapitulation of multiple developmental processes and activation of latent wound repair mechanisms conserved among vertebrates. However, the process of tail regeneration in the lizard does not match the dedifferentiation and blastema-based model as described in the salamander and zebrafish, and instead matches a model involving tissue-specific regeneration through stem/progenitor populations. The pattern of cell proliferation and tissue formation in the lizard identifies a uniquely amniote vertebrate combination of multiple developmental and repair mechanisms. We anticipate that the conserved genetic mechanisms observed in regeneration of the lizard tail may have particular relevance for development of regenerative medical approaches.

## Supporting Information

Figure S1 **Gene Ontology analysis of differentially expressed genes identified by both Cuffdiff2 and DESeq2.** 130 genes were identified as differentially expressed by both methods (Figure 1B-C; Table S3; Table S4). (*A-B*) A treemap overview of differentially expressed genes in (*A*) Cluster I and (*B*) Cluster II based on representative Gene Ontology Biological Processes. The relative sizes of the treemap boxes are based on the |log10(p-value)| of the respective GO term. Related terms are visualized with the same color, with the representative category for each color group denoted in the legend.
(EPS)

Figure S2 **Genes with high expression (>10-fold change) in the regenerating tail relative to the embryos and satellite cells.** 44 differentially expressed genes had >10-fold change in S1 and S2 gene expression (FPKM) relative to the embryos and satellite cells (orange cluster) and 86 genes had >10-fold change in S4 and S5 (yellow cluster).
(EPS)

Figure S3 **Satellite cells isolated from adult skeletal muscle express PAX7 and can differentiate into myotubes.** (*A-B*) Detection of myosin heavy chain (MHC) in proliferating (*A*) and differentiated (*B*) *A. carolinensis* satellite cells. MHC was detected using MY-32 monoclonal antibody and HRP-conjugated anti-mouse secondary antibody with DAB stain. Immunofluorescence of lizard (*C-F*) and mouse (*G-H*) satellite cells. (*C-D, G-H*) PAX7 was detected using a monoclonal antibody and visualized by FITC-conjugated anti-mouse secondary antibody, and nuclei were stained with DAPI. (*E-F*) Cells with no primary antibody and FITC-conjugated anti-mouse secondary antibody only, and nuclei stained with DAPI.
(TIF)

Figure S4 **Histological analysis of proliferation in the 25 dpa regenerating tail.** (*A-G*) PCNA immunohistochemistry of the 25 dpa regenerating tail (brown nuclei), counterstained with hematoxylin (blue nuclei). (*A*) PCNA is expressed throughout the regenerating tail, indicating a lack of a single proliferative zone. The dermis near the proximal base (*B*) and tip (*C*) of the regenerating tail, condensing cartilage tube (*D*), ependymal core (*E*), and developing muscles near the proximal base (*F*) and tip (*G*) of the regenerating tail all show PCNA positive cells.
(TIF)

Figure S5 **Immunohistochemistry shows few proliferating cells in the original tail.** (*A-D*) Proliferating cell nuclear antigen (PCNA) immunohistochemistry of the original tail (brown nuclei), counterstained with hematoxylin (blue nuclei). (*A*) Transverse section of the original tail. (*B-D*) There are limited PCNA-positive cells in the centrum (*B*), skeletal muscle (*C*) and skin (*D*). There is some endogenous pigmentation due to chromatophores in the skin (*D*). (*F*) Original tail no primary antibody control, counterstained with hematoxylin. Composites: *A* & *F*. Scale bars: 200 µm (*A, F*), 20 µm (*B-D*).
(TIF)

Table S1 ***A. carolinensis*** **genome annotation version 2.2.1 in comparison with previous annotations.**
(DOCX)

Table S2 **Summary of RNA-Seq reads.**
(DOCX)

Table S3 **Differentially expressed genes in the lizard regenerating tail at 25 dpa analyzed by Cuffdiff2.**
(DOCX)

Table S4 **Differentially expressed genes in the lizard regenerating tail at 25 dpa analyzed by DESeq2.**
(DOCX)

Table S5 **GO Biological Process analysis (DAVID) on differentially expressed genes in the proximal regenerating tail (Cluster I).**
(DOCX)

Table S6 **GO Biological Process analysis (DAVID) on differentially expressed genes in the regenerating tail tip (Cluster II).**
(DOCX)

Table S7 **KEGG pathway analysis (DAVID) on differentially expressed genes in the 25 dpa regenerating tail.**
(DOCX)

Table S8 **Differentially expressed genes elevated (10-fold) in the regenerating tip compared to embryo and satellite cells.**
(DOCX)

Table S9 **Differentially genes elevated (10-fold) in the proximal regenerating tail compared to embryo and satellite cells.**
(DOCX)
