# Supplementary figures and images for: Transcriptomic Analysis of Tail Regeneration in the Lizard Anolis carolinensis Reveals Activation of Conserved Vertebrate Developmental and Repair Mechanisms
Source: PLoS One. 2014 Aug 20;9(8):e105004. doi: 10.1371/journal.pone.0105004 (PMC4139331; doi:10.1371/journal.pone.0105004)

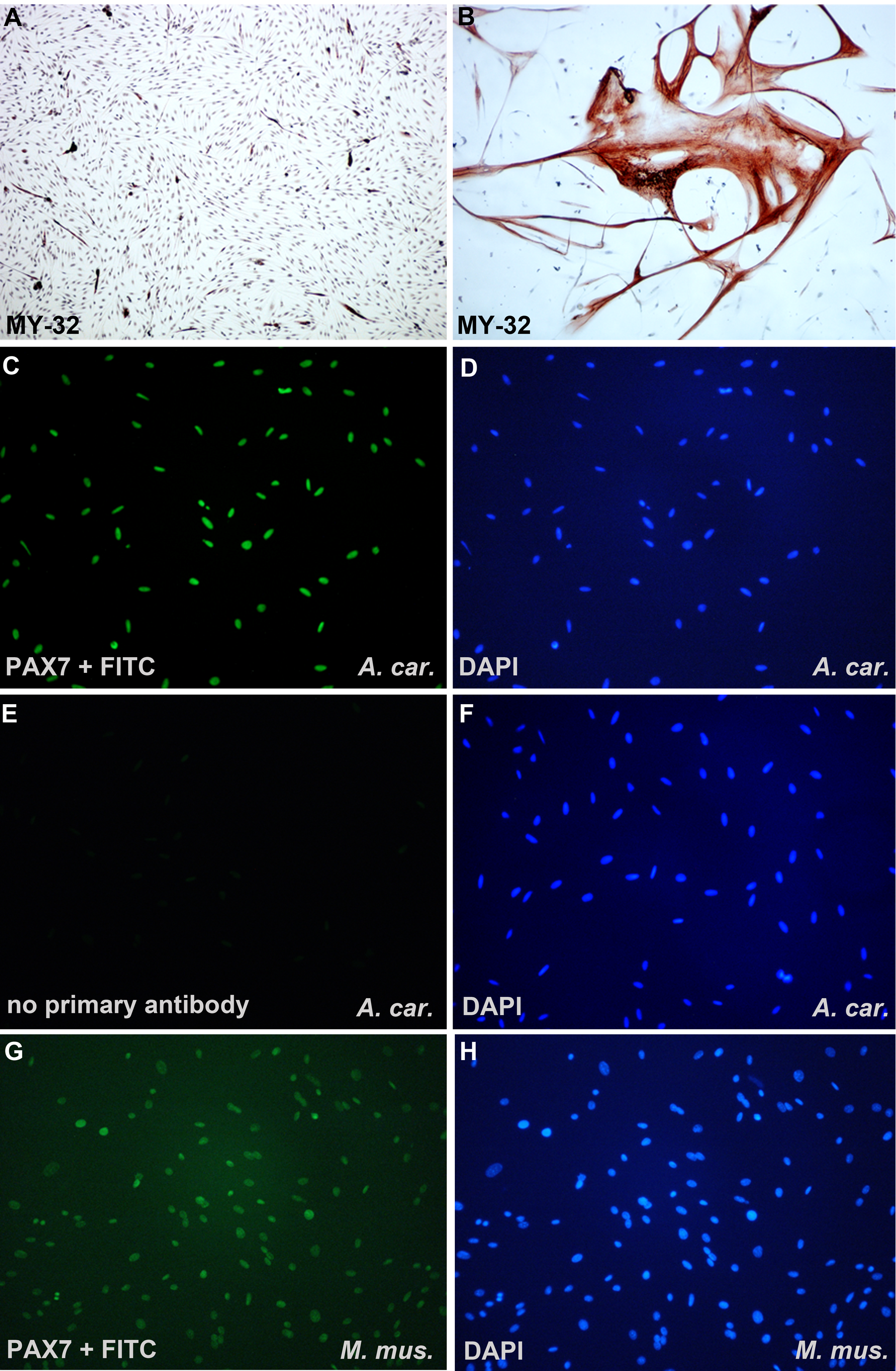

Supplement: Figure S3 — Satellite cells isolated from adult skeletal muscle express PAX7 and can differentiate into myotubes. (A-B) Detection of myosin heavy chain (MHC) in proliferating (A) and differentiated (B) A. carolinensis satellite cells. MHC was detected using MY-32 monoclonal antibody and HRP-conjugated anti-mouse secondary antibody with DAB stain. Immunofluorescence of lizard (C-F) and mouse (G-H) satellite cells. (C-D, G-H) PAX7 was detected using a monoclonal antibody and visualized by FITC-conjugated anti-mouse secondary antibody, and nuclei were stained with DAPI. (E-F) Cells with no primary antibody and FITC-conjugated anti-mouse secondary antibody only, and nuclei stained with DAPI. (TIF) [file pone.0105004.s003.tif]

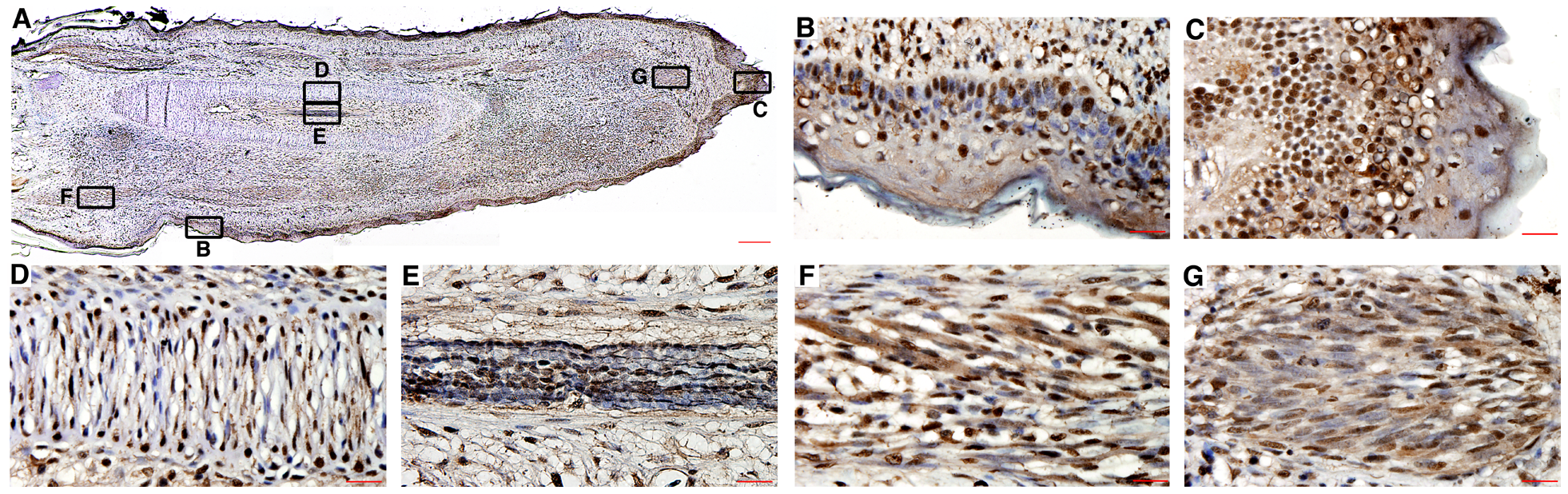

Supplement: Figure S4 — Histological analysis of proliferation in the 25 dpa regenerating tail. (A-G) PCNA immunohistochemistry of the 25 dpa regenerating tail (brown nuclei), counterstained with hematoxylin (blue nuclei). (A) PCNA is expressed throughout the regenerating tail, indicating a lack of a single proliferative zone. The dermis near the proximal base (B) and tip (C) of the regenerating tail, condensing cartilage tube (D), ependymal core (E), and developing muscles near the proximal base (F) and tip (G) of the regenerating tail all show PCNA positive cells. (TIF) [file pone.0105004.s004.tif]

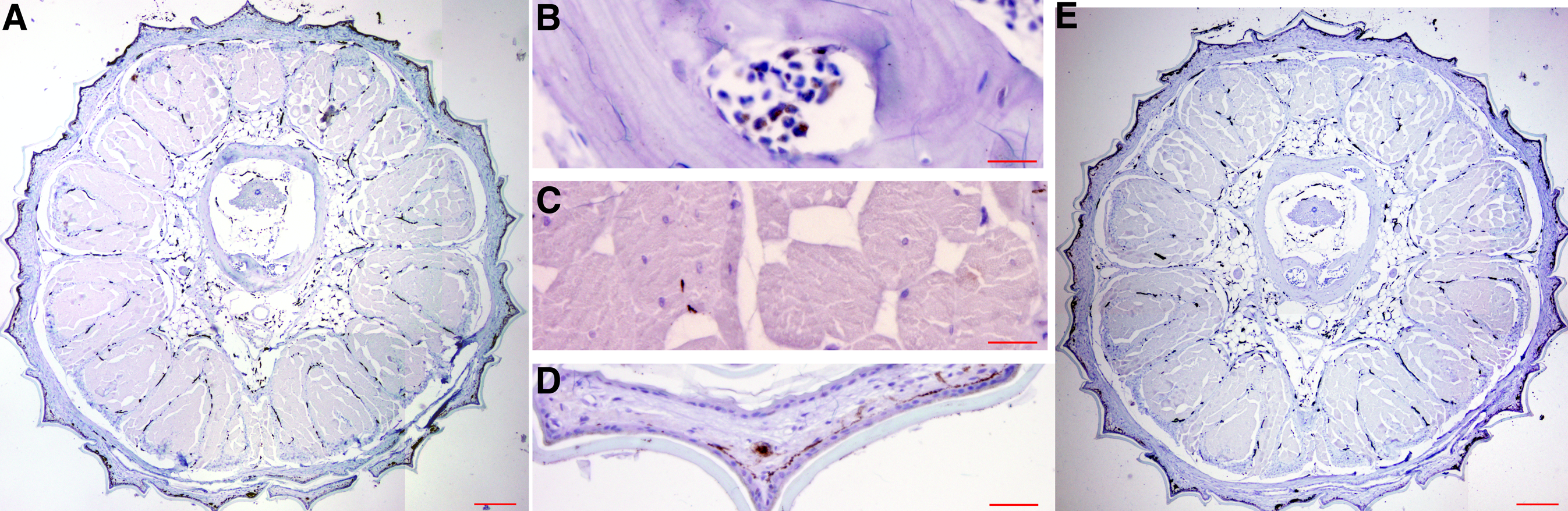

Supplement: Figure S5 — Immunohistochemistry shows few proliferating cells in the original tail. (A-D) Proliferating cell nuclear antigen (PCNA) immunohistochemistry of the original tail (brown nuclei), counterstained with hematoxylin (blue nuclei). (A) Transverse section of the original tail. (B-D) There are limited PCNA-positive cells in the centrum (B), skeletal muscle (C) and skin (D). There is some endogenous pigmentation due to chromatophores in the skin (D). (F) Original tail no primary antibody control, counterstained with hematoxylin. Composites: A & F. Scale bars: 200 µm (A, F), 20 µm (B-D). (TIF) [file pone.0105004.s005.tif]
