# Supplementary material for: Transcriptomic Analysis of Tail Regeneration in the Lizard Anolis carolinensis Reveals Activation of Conserved Vertebrate Developmental and Repair Mechanisms
Source: PLoS One. 2014 Aug 20;9(8):e105004. doi: 10.1371/journal.pone.0105004 (PMC4139331; doi:10.1371/journal.pone.0105004)
Supplement: Table S1 — A. carolinensis genome annotation version 2.2.1 in comparison with previous annotations. (DOCX) [file pone.0105004.s006.docx]

**Table S1. *Anolis carolinensis* genome annotation version 2.2.1 in comparison with current annotations.**

| **Overview** | **ASU v2.2.1** | **ASU v2.1** | **NCBI** | **Ensembl 74** |
| --- | --- | --- | --- | --- |
| Annotated genes | 22,960 | 22,962 | 15,645 | 21,913 |
| Annotated transcript isoforms | 65,670 | 59,373 | 16,533 | 22,494 |
| Annotated isoforms/gene | 2.86 | 2.59 | 1.06 | 1.03 |
| **Annotated Transcripts** |  |  |  |  |
| All transcript isoforms | 65,670 | 59,373 | 16,533 | 22,494 |
| Transcripts with start & stop codons | 59,514 | 53,401 | 14,667 | 11,503 |
| Transcripts missing start or stop codon | 6,156 | 5,972 | 1,866 | 10,991 |
| Single exon transcripts | 2,098 | 2,070 | 983 | 724 |
| Transcript N50 length | 5,325 | 5,355 | 2,364 | 2,094 |
| Average coding sequence length | 2,010 | 1,964 | 1,701 | 1,353 |
| **Exons** |  |  |  |  |
| Total number of exons | 234,269 | 229,204 | 156,742 | 179,549 |
| Exons with start codon | 32,256 | 29,677 | 13,512 | 12,488 |
| Exons without start or stop codon | 168,195 | 168,367 | 128,486 | 153,121 |
| Exons with stop codon | 32,419 | 29,727 | 13,779 | 13,225 |
| Exons/annotated transcript | 12.35 | 12.05 | 10.11 | 8.20 |
| Average exon length | 170 | 170 | 170 | 166 |
| Total exon length | 39,715,013 | 38,902,806 | 26,658,387 | 29,723,416 |
| **3' UTR** |  |  |  |  |
| Total transcripts with 3'UTR | 38,938 | 34,926 | 5,861 | 38,938 |
| Average length of transcripts with 3'UTR | 1,086 | 1,168 | 456 | 866 |
| Total 3'UTR sequence length | 42,269,162 | 40,798,794 | 2,674,388 | 15,251,066 |
| **5' UTR** |  |  |  |  |
| Total transcripts with 5'UTR | 52,377 | 46,782 | 6,168 | 16,285 |
| Average length of transcripts with 5'UTR | 244 | 244 | 86 | 232 |
| Total 5'UTR sequence length | 12,774,153 | 11,422,626 | 527,454 | 3,776,492 |
| **Introns** |  |  |  |  |
| Total number of introns | 192,478 | 192,418 | 141,362 | 160,644 |
| Average intron length | 4,520 | 4,525 | 4,463 | 3,445 |
| Total intron sequence length | 869,951,095 | 870,771,088 | 630,937,171 | 553,391,127 |
