# Supplementary material for: Transcriptomic Analysis of Tail Regeneration in the Lizard Anolis carolinensis Reveals Activation of Conserved Vertebrate Developmental and Repair Mechanisms
Source: PLoS One. 2014 Aug 20;9(8):e105004. doi: 10.1371/journal.pone.0105004 (PMC4139331; doi:10.1371/journal.pone.0105004)
Supplement: Table S2 — Summary of RNA-Seq reads. (DOCX) [file pone.0105004.s007.docx]

| **Table S2. Summary of RNA-seq reads.** | | | |
| --- | --- | --- | --- |
| **Sample** | **Raw reads** | **Trimmed read length** | **Total Mapped reads** |
| S1-1 | 202013710 | 104 bp | 126571996 |
| S2-1 | 279865440 | 104 bp | 188030955 |
| S3-1 | 154497145 | 104 bp | 99124655 |
| S4-1 | 177141461 | 104 bp | 113841805 |
| S5-1 | 136875821 | 104 bp | 86330387 |
| S1-2 | 129878748 | 39 bp | 109499590 |
| S2-2 | 98991358 | 39 bp | 81644518 |
| S3-2 | 114729492 | 39 bp | 90721840 |
| S4-2 | 46608697 | 39 bp | 37337580 |
| S5-2 | 106731245 | 39 bp | 82393206 |
| S1-3 | 95142862 | 39 bp | 78939978 |
| S2-3 | 109623596 | 39 bp | 94598693 |
| S3-3 | 67342607 | 39 bp | 50112902 |
| S4-3 | 109255766 | 39 bp | 91876649 |
| S5-3 | 75632402 | 39 bp | 63568005 |
| S1-4 | 91027920 | 39 bp | 69515324 |
| S2-4 | 51439381 | 39 bp | 40386483 |
| S3-4 | 70110896 | 39 bp | 47729837 |
| S4-4 | 67671088 | 39 bp | 54187416 |
| S5-4 | 96620231 | 39 bp | 80285422 |
| S1-5 | 93556757 | 39 bp | 80266153 |
| S2-5 | 108564680 | 39 bp | 90029267 |
| S3-5 | 104438526 | 39 bp | 88530228 |
| S4-5 | 77584875 | 39 bp | 65843773 |
| S5-5 | 83281664 | 39 bp | 73018519 |
| embryo-28S | 128096847 | 104 bp | 82731774 |
| embryo-38S | 141809501 | 104 bp | 93817244 |
| satellite cells-1 | 182885980 | 104 bp | 117608584 |
| satellite cells-2 | 159655966 | 39 bp | 123024548 |
| satellite cells-3 | 149407331 | 39 bp | 125752052 |
