# Supplementary material for: Transcriptomic Analysis of Tail Regeneration in the Lizard Anolis carolinensis Reveals Activation of Conserved Vertebrate Developmental and Repair Mechanisms
Source: PLoS One. 2014 Aug 20;9(8):e105004. doi: 10.1371/journal.pone.0105004 (PMC4139331; doi:10.1371/journal.pone.0105004)
Supplement: Table S3 — Differentially expressed genes in the lizard regenerating tail at 25 dpa analyzed by Cuffdiff2. (DOCX) [file pone.0105004.s008.docx]

| **Table S3. Differentially expressed genes in the lizard regenerating tail at 25 dpa analyzed by Cuffdiff2.** | | | | | |
| --- | --- | --- | --- | --- | --- |
| **Cluster** | **Gene** | **NCBI_ID** | **ensembl_ID** | **Ortholog** | **Orthologous Gene Description** |
| I | ASU_Acar_G.13672 | 100560196 | ENSACAG00000011592 | ***ablim2*** | actin binding LIM protein family, member 2 |
| I | ASU_Acar_G.21712 | 100554326 | ENSACAG00000013375 | ***acan*** | aggrecan |
| I | ASU_Acar_G.1152 | 100566789 | ENSACAG00000000836 | ***acta1*** | actin, alpha 1, skeletal muscle |
| I | ASU_Acar_G.859 | 100560364 | ENSACAG00000001104 | ***actc1*** | actin, alpha, cardiac muscle 1 |
| I | ASU_Acar_G.1793 | 100556098 | ENSACAG00000002769 | ***actn2*** | actinin, alpha 2 |
| I | ASU_Acar_G.20827 | 100556084 | ENSACAG00000008455 | ***actn3*** | actinin, alpha 3 (gene/pseudogene) |
| I | ASU_Acar_G.12270 | 100557764 | ENSACAG00000017722 | ***adamts15*** | ADAM metallopeptidase with thrombospondin type 1 motif, 15 |
| I | ASU_Acar_G.22820 | 100561111 | - | ***adamts18*** | ADAM metallopeptidase with thrombospondin type 1 motif, 18 |
| I | ASU_Acar_G.8360 | 100557229 | ENSACAG00000003071 | ***adamts20*** | ADAM metallopeptidase with thrombospondin type 1 motif, 20 |
| I | ASU_Acar_G.15610 | 100566047 | ENSACAG00000010688 | ***aff2*** | AF4/FMR2 family, member 2 |
| I | ASU_Acar_G.4813 | 100555454 | ENSACAG00000002643 | ***akap6*** | A kinase (PRKA) anchor protein 6 |
| I | ASU_Acar_G.13003 | 100558159 | ENSACAG00000010018 | ***alpk2*** | alpha-kinase 2 |
| I | ASU_Acar_G.18361 | - | ENSACAG00000006448 | ***alpk3*** | alpha-kinase 3 |
| I | ASU_Acar_G.1864 | 100560418 | ENSACAG00000008625 | ***asb2*** | ankyrin repeat and SOCS box containing 2 |
| I | ASU_Acar_G.21279 | 100557723 | ENSACAG00000003749 | ***atp1b4*** | ATPase, Na+/K+ transporting, beta 4 polypeptide |
| I | ASU_Acar_G.15160 | 100554310 | ENSACAG00000005552 | ***atp2a1*** | ATPase, Ca++ transporting, cardiac muscle, fast twitch 1 |
| I | ASU_Acar_G.2894 | 100558455 | ENSACAG00000016768 | ***bhmt*** | betaine--homocysteine S-methyltransferase |
| I | ASU_Acar_G.8629 | 100563219 | ENSACAG00000010162 | ***bmp3*** | bone morphogenetic protein 3 |
| I | ASU_Acar_G.15385 | - | ENSACAG00000025734 | ***c10orf71*** | chromosome 10 open reading frame 71 |
| I | ASU_Acar_G.5029 | 100556575 | ENSACAG00000001970 | ***c21orf7*** | chromosome 21 open reading frame 7 |
| I | ASU_Acar_G.5693 | - | ENSACAG00000006295 | ***c2orf82*** | chromosome 2 open reading frame 82 |
| I | ASU_Acar_G.7058 | 100558336 | ENSACAG00000024792 | ***ca3*** | carbonic anhydrase III, muscle specific |
| I | ASU_Acar_G.8645 | 100566428 | ENSACAG00000012663 | ***cacna2d1*** | calcium channel, voltage-dependent, alpha 2/delta subunit 1 |
| I | ASU_Acar_G.10230 | 100564526 | ENSACAG00000017422 | ***cacnb1*** | calcium channel, voltage-dependent, beta 1 subunit |
| I | ASU_Acar_G.3740 | 100563784 | ENSACAG00000015036 | ***camk2a*** | calcium/calmodulin-dependent protein kinase II alpha |
| I | ASU_Acar_G.21481 | 100554653 | ENSACAG00000015755 | ***casq1*** | calsequestrin 1 (fast-twitch, skeletal muscle) |
| I | ASU_Acar_G.4130 | 100566014 | ENSACAG00000003187 | ***casq2*** | calsequestrin 2 (cardiac muscle) |
| I | ASU_Acar_G.8563 | 100557562 | ENSACAG00000007661 | ***celf2*** | CUGBP, Elav-like family member 2 |
| I | ASU_Acar_G.4064 | 100553103 | ENSACAG00000002385 | ***cfl2*** | cofilin 2 (muscle) |
| I | ASU_Acar_G.16830 | 100557909 | ENSACAG00000005293 | ***chrna1*** | cholinergic receptor, nicotinic, alpha 1 (muscle) |
| I | ASU_Acar_G.22238 | 100554201 | ENSACAG00000014790 | ***chrne*** | cholinergic receptor, nicotinic, epsilon (muscle) |
| I | ASU_Acar_G.15491 | 100565393 | ENSACAG00000010910 | ***chrng*** | cholinergic receptor, nicotinic, gamma (muscle) |
| I | ASU_Acar_G.22561 | 100562721 | ENSACAG00000005396 | ***ckm*** | creatine kinase, muscle |
| I | ASU_Acar_G.17769 | 100555752 | ENSACAG00000010347 | ***clec3a*** | C-type lectin domain family 3, member A |
| I | ASU_Acar_G.5548 | 100555527 | ENSACAG00000003514 | ***clstn2*** | calsyntenin 2 |
| I | ASU_Acar_G.20941 | 100557792 | ENSACAG00000003040 | ***col11a2*** | collagen, type XI, alpha 2 |
| I | ASU_Acar_G.7464 | 100558670 | ENSACAG00000012191 | ***col22a1*** | collagen, type XXII, alpha 1 |
| I | ASU_Acar_G.10144 | 100562364 | ENSACAG00000013078 | ***col28a1*** | collagen, type XXVIII, alpha 1 |
| I | ASU_Acar_G.18867 | 100553016 | ENSACAG00000006064 | ***col2a1*** | collagen, type II, alpha 1 |
| I | ASU_Acar_G.987 | 100567245 | ENSACAG00000008314 | ***col9a1*** | collagen, type IX, alpha 1 |
| I | ASU_Acar_G.18455 | 100565206 | ENSACAG00000004683 | ***col9a2*** | collagen, type IX, alpha 2 |
| I | ASU_Acar_G.7008 | 100554037 | ENSACAG00000005213 | ***col9a3*** | collagen, type IX, alpha 3 |
| I | ASU_Acar_G.10941 | 100555576 | - | ***cpped1*** | calcineurin-like phosphoesterase domain-containing protein 1 |
| I | ASU_Acar_G.9893 | 100560783 | ENSACAG00000009025 | ***csgalnact1*** | chondroitin sulfate N-acetylgalactosaminyltransferase 1 |
| I | ASU_Acar_G.116 | 100566206 | ENSACAG00000015769 | ***csrp3*** | cysteine and glycine-rich protein 3 (cardiac LIM protein) |
| I | ASU_Acar_G.22949 | 100556256 | ENSACAG00000013760 | ***cyp2f3*** | cytochrome P450 2F3 |
| I | ASU_Acar_G.2218 | 100560815 | ENSACAG00000013847 | ***des*** | desmin |
| I | ASU_Acar_G.4806 | 100556174 | ENSACAG00000016930 | ***dhrs7c*** | dehydrogenase/reductase (SDR family) member 7C |
| I | ASU_Acar_G.12798 | 100561061 | ENSACAG00000006911 | ***dtna*** | dystrobrevin, alpha |
| I | ASU_Acar_G.2919 | - | ENSACAG00000009767 | ***ecm2*** | extracellular matrix protein 2 |
| I | ASU_Acar_G.6665 | 100561171 | ENSACAG00000006007 | ***eef1a2*** | eukaryotic translation elongation factor 1 alpha 2 |
| I | ASU_Acar_G.6389 | 100563528 | ENSACAG00000001659 | ***egfl6*** | EGF-like-domain, multiple 6 |
| I | ASU_Acar_G.5093 | 100567907 | ENSACAG00000002030 | ***enox1*** | ecto-NOX disulfide-thiol exchanger 1 |
| I | ASU_Acar_G.9223 | 100560848 | ENSACAG00000012287 | ***epyc*** | epiphycan |
| I | ASU_Acar_G.12447 | 100566174 | ENSACAG00000010346 | ***eya4*** | eyes absent homolog 4 (Drosophila) |
| I | ASU_Acar_G.11555 | 100556426 | ENSACAG00000008514 | ***fgf13*** | fibroblast growth factor 13 |
| I | ASU_Acar_G.19610 | 100557719 | ENSACAG00000011540 | ***fgfrl1*** | fibroblast growth factor receptor-like 1 |
| I | ASU_Acar_G.13814 | 100561063 | ENSACAG00000004747 | ***fibin*** | fin bud initiation factor homolog (zebrafish) |
| I | ASU_Acar_G.21948 | 100555896 | ENSACAG00000009029 | ***flnb*** | filamin B |
| I | ASU_Acar_G.21857 | 100559302 | ENSACAG00000009986 | ***flnc*** | filamin C, gamma |
| I | ASU_Acar_G.7172 | 100565108 | ENSACAG00000002165 | ***fmod*** | fibromodulin |
| I | ASU_Acar_G.5031 | 100565823 | ENSACAG00000002739 | ***fzd4*** | frizzled family receptor 4 |
| I | ASU_Acar_G.14133 | - | - | **G.14133** | gag-pol polyprotein |
| I | ASU_Acar_G.14483 | - | - | **G.14483** | predicted lncRNA |
| I | ASU_Acar_G.15880 | - | - | **G.15880** | unknown protein-coding with DUF4585 domain |
| I | ASU_Acar_G.1922 | - | ENSACAG00000028169 | **G.1922** | mir-133 |
| I | ASU_Acar_G.19355 | 100554070 | - | **G.19355** | mir-324 |
| I | ASU_Acar_G.4168 | - | - | **G.4168** | rna-directed dna polymerase from mobile element jockey-like |
| I | ASU_Acar_G.591 | - | - | **G.591** | gag-pol polyprotein |
| I | ASU_Acar_G.7180 | - | - | **G.7180** | predicted lncRNA |
| I | ASU_Acar_G.8849 | - | - | **G.8849** | predicted lncRNA |
| I | ASU_Acar_G.4437 | 100555782 | ENSACAG00000017003 | ***gas7*** | growth arrest-specific 7 |
| I | ASU_Acar_G.3693 | 100555587 | ENSACAG00000011432 | ***hapln1*** | hyaluronan and proteoglycan link protein 1 |
| I | ASU_Acar_G.10140 | 100564922 | ENSACAG00000006760 | ***hhatl*** | hedgehog acyltransferase-like |
| I | ASU_Acar_G.16488 | 100563683 | ENSACAG00000017205 | ***hsd17b14*** | 17-beta-hydroxysteroid dehydrogenase 14 |
| I | ASU_Acar_G.12359 | 100552402 | ENSACAG00000010030 | ***itga7*** | integrin, alpha 7 |
| I | ASU_Acar_G.7938 | 100562750 | ENSACAG00000016733 | ***jph2*** | junctophilin 2 |
| I | ASU_Acar_G.12249 | 100553783 | ENSACAG00000008216 | ***kcnma1*** | potassium large conductance calcium-activated channel, subfamily M, alpha member 1 |
| I | ASU_Acar_G.21385 | - | ENSACAG00000010805 | ***klhl20*** | kelch-like protein 20 |
| I | ASU_Acar_G.1581 | 100551911 | ENSACAG00000008404 | ***klhl31*** | kelch-like family member 31 |
| I | ASU_Acar_G.12414 | 100560271 | ENSACAG00000014492 | ***ldb3*** | LIM domain binding 3 |
| I | ASU_Acar_G.6073 | 100567514 | ENSACAG00000000442 | ***lect1*** | leukocyte cell derived chemotaxin 1 |
| I | ASU_Acar_G.9579 | 100553447 | ENSACAG00000012474 | ***lgals1*** | Galectin-1 |
| I | ASU_Acar_G.16693 | 100379172 | ENSACAG00000024814 | ***li-ac-27*** | Keratin-associated beta-protein 27 |
| I | ASU_Acar_G.9353 | 100563152 | ENSACAG00000012067 | ***limch1*** | LIM and calponin homology domains 1 |
| I | ASU_Acar_G.2472 | 100566155 | ENSACAG00000015388 | ***lmod3*** | leiomodin 3 (fetal) |
| I | ASU_Acar_G.16578 | 100554903 | - | ***lpp*** | LIM domain containing preferred translocation partner in lipoma |
| I | ASU_Acar_G.22907 | - | ENSACAG00000003759 | ***ltbp4*** | latent transforming growth factor beta binding protein 4 |
| I | ASU_Acar_G.22910 | 100564202 | ENSACAG00000003759 | ***ltbp4*** | latent transforming growth factor beta binding protein 4 |
| I | ASU_Acar_G.19146 | 100559813 | ENSACAG00000000957 | ***matn1*** | matrilin 1, cartilage matrix protein |
| I | ASU_Acar_G.8050 | 100560579 | ENSACAG00000012284 | ***matn4*** | matrilin 4 |
| I | ASU_Acar_G.1321 | 100553692 | ENSACAG00000009301 | ***mdga1*** | MAM domain containing glycosylphosphatidylinositol anchor 1 |
| I | ASU_Acar_G.4723 | 100559834 | ENSACAG00000000488 | ***mef2c*** | myocyte enhancer factor 2C |
| I | ASU_Acar_G.6212 | 100555258 | ENSACAG00000015543 | ***megf6*** | multiple epidermal growth factor-like domains protein 6 |
| I | ASU_Acar_G.5161 | 100553237 | ENSACAG00000002751 | ***mettl21e*** | Protein-lysine methyltransferase |
| I | ASU_Acar_G.2988 | 100563976 | ENSACAG00000017439 | ***mfap5*** | microfibrillar associated protein 5 |
| I | ASU_Acar_G.8449 | 100551807 | ENSACAG00000010792 | ***mgp*** | matrix Gla protein |
| I | ASU_Acar_G.8581 | 100566807 | - | ***mgp*** | matrix Gla protein |
| I | ASU_Acar_G.14862 | 100554244 | ENSACAG00000026043 | ***mkl2*** | myocardin-related transcription factor B |
| I | ASU_Acar_G.10180 | 100563668 | ENSACAG00000007829 | ***mkx*** | mohawk homeobox |
| I | ASU_Acar_G.7373 | 100556384 | ENSACAG00000014585 | ***msc*** | musculin |
| I | ASU_Acar_G.2697 | 100557219 | ENSACAG00000004208 | ***mstn*** | myostatin |
| I | ASU_Acar_G.8132 | 100562221 | ENSACAG00000001796 | ***murc*** | muscle-related coiled-coil protein |
| I | ASU_Acar_G.9162 | 100560974 | ENSACAG00000016657 | ***mybpc1*** | myosin binding protein C, slow type |
| I | ASU_Acar_G.10232 | 100558218 | ENSACAG00000012342 | ***mybpc2*** | myosin binding protein C, fast type |
| I | ASU_Acar_G.1763 | 100554792 | ENSACAG00000002510 | ***mybpc3*** | myosin binding protein C, cardiac |
| I | ASU_Acar_G.19997 | 100567683 | ENSACAG00000006296 | ***myh6*** | myosin-6 |
| I | ASU_Acar_G.387 | 100558249 | ENSACAG00000010643 | ***myl1*** | myosin, light chain 1, alkali; skeletal, fast |
| I | ASU_Acar_G.19536 | 100561920 | ENSACAG00000015270 | ***myl10*** | myosin, light chain 10, regulatory |
| I | ASU_Acar_G.17474 | 100566191 | ENSACAG00000002200 | ***myl2*** | myosin, light chain 2, regulatory, cardiac, slow |
| I | ASU_Acar_G.17789 | 100557845 | ENSACAG00000005125 | ***myl3*** | myosin, light chain 3, alkali; ventricular, skeletal, slow |
| I | ASU_Acar_G.3409 | 100561943 | ENSACAG00000022592 | ***myl6b*** | myosin, light chain 6B, alkali, smooth muscle and non-muscle |
| I | ASU_Acar_G.15644 | 100567803 | ENSACAG00000011501 | ***mylk4*** | myosin light chain kinase family, member 4 |
| I | ASU_Acar_G.18654 | 100559161 | ENSACAG00000010684 | ***mylpf*** | myosin light chain, phosphorylatable, fast skeletal muscle |
| I | ASU_Acar_G.469 | 100562663 | ENSACAG00000000054 | ***myod1*** | myogenic differentiation 1 |
| I | ASU_Acar_G.6620 | 100565825 | ENSACAG00000009246 | ***myom1*** | myomesin 1 |
| I | ASU_Acar_G.1040 | 100564167 | ENSACAG00000004962 | ***myom2*** | myomesin 2 |
| I | ASU_Acar_G.18467 | 100563435 | ENSACAG00000005357 | ***myom3*** | myomesin 3 |
| I | ASU_Acar_G.3730 | 100559517 | ENSACAG00000017085 | ***myot*** | myotilin |
| I | ASU_Acar_G.9302 | 100567139 | ENSACAG00000013422 | ***myoz2*** | myozenin 2 |
| I | ASU_Acar_G.13031 | 100564401 | ENSACAG00000010443 | ***mypn*** | myopalladin |
| I | ASU_Acar_G.15473 | 100563097 | ENSACAG00000003769 | ***neb*** | nebulin |
| I | ASU_Acar_G.22777 | 100558548 | ENSACAG00000013742 | ***neto2*** | neuropilin (NRP) and tolloid (TLL)-like 2 |
| I | ASU_Acar_G.6606 | 100565300 | ENSACAG00000000941 | ***nfatc1*** | nuclear factor of activated T-cells, cytoplasmic, calcineurin-dependent 1 |
| I | ASU_Acar_G.12694 | 100561114 | ENSACAG00000010135 | ***nrap*** | nebulin-related anchoring protein |
| I | ASU_Acar_G.10284 | 100566306 | ENSACAG00000006865 | ***obscn*** | obscurin, cytoskeletal calmodulin and titin-interacting RhoGEF |
| I | ASU_Acar_G.1835 | 100561483 | ENSACAG00000014245 | ***obsl1*** | obscurin-like 1 |
| I | ASU_Acar_G.11956 | 100553158 | - | ***pax7*** | paired box protein Pax-7-like |
| I | ASU_Acar_G.5742 | 100561752 | ENSACAG00000004684 | ***pcolce2*** | procollagen C-endopeptidase enhancer 2 |
| I | ASU_Acar_G.7486 | 100559258 | ENSACAG00000013127 | ***pde4dip*** | phosphodiesterase 4D interacting protein |
| I | ASU_Acar_G.9201 | 100561175 | ENSACAG00000002047 | ***pdlim3*** | PDZ and LIM domain 3 |
| I | ASU_Acar_G.13772 | 100567602 | ENSACAG00000002010 | ***pgam2*** | phosphoglycerate mutase 2 (muscle) |
| I | ASU_Acar_G.17657 | 100558233 | ENSACAG00000006657 | ***plod2*** | procollagen-lysine, 2-oxoglutarate 5-dioxygenase 2 |
| I | ASU_Acar_G.8110 | 100551935 | ENSACAG00000006771 | ***prkaa2*** | protein kinase, AMP-activated, alpha 2 catalytic subunit |
| I | ASU_Acar_G.4222 | 100551917 | ENSACAG00000004644 | ***ptprd*** | protein tyrosine phosphatase, receptor type, D |
| I | ASU_Acar_G.20064 | 100560997 | ENSACAG00000027048 | ***ptx4*** | pentraxin 4, long |
| I | ASU_Acar_G.22394 | 100566844 | ENSACAG00000012295 | ***pvalb*** | parvalbumin beta |
| I | ASU_Acar_G.22145 | 100553610 | ENSACAG00000005131 | ***pygm*** | phosphorylase, glycogen, muscle |
| I | ASU_Acar_G.10175 | 100563157 | ENSACAG00000011931 | ***rbfox2*** | RNA binding protein, fox-1 homolog (C. elegans) 2 |
| I | ASU_Acar_G.7055 | 100563922 | ENSACAG00000003962 | ***rbm24*** | RNA binding motif protein 24 |
| I | ASU_Acar_G.19788 | 100551835 | ENSACAG00000008872 | ***rpl3l*** | ribosomal protein L3-like |
| I | ASU_Acar_G.10808 | 100559227 | ENSACAG00000007451 | ***rtn2*** | reticulon 2 |
| I | ASU_Acar_G.17542 | 100558429 | ENSACAG00000001965 | ***ryr1*** | ryanodine receptor 1 |
| I | ASU_Acar_G.22456 | 100562261 | ENSACAG00000003150 | ***ryr1*** | ryanodine receptor 1 (skeletal) |
| I | ASU_Acar_G.11790 | - | - | ***ryr1*** | ryanodine receptor 1-like (skeletal) (predicted) |
| I | ASU_Acar_G.14162 | 100563744 | ENSACAG00000011002 | ***sbk2*** | SH3-binding domain kinase family, member 2 |
| I | ASU_Acar_G.18813 | 100565207 | ENSACAG00000009159 | ***scn5a*** | sodium channel, voltage-gated, type V, alpha subunit |
| I | ASU_Acar_G.15641 | 100552019 | ENSACAG00000024865 | ***serpinb2*** | serpin peptidase inhibitor, clade B (ovalbumin), member 2 |
| I | ASU_Acar_G.9126 | 100563804 | ENSACAG00000015748 | ***sfrp2*** | secreted frizzled-related protein 2 |
| I | ASU_Acar_G.2060 | 100564894 | ENSACAG00000004861 | ***slc15a2*** | solute carrier family 15 (H+/peptide transporter), member 2 |
| I | ASU_Acar_G.7677 | 100553181 | ENSACAG00000003406 | ***slc25a22*** | solute carrier family 25 (mitochondrial carrier: glutamate), member 22 |
| I | ASU_Acar_G.8253 | 100563345 | ENSACAG00000002352 | ***slc25a4*** | solute carrier family 25 (mitochondrial carrier; adenine nucleotide translocator), member 4 |
| I | ASU_Acar_G.9265 | 100558087 | ENSACAG00000001304 | ***slit2*** | slit homolog 2 (Drosophila) |
| I | ASU_Acar_G.2023 | 100563775 | ENSACAG00000003213 | ***smoc2*** | SPARC related modular calcium binding 2 |
| I | ASU_Acar_G.10684 | 100561699 | ENSACAG00000008484 | ***smyd1*** | SET and MYND domain containing 1 |
| I | ASU_Acar_G.4165 | 100567184 | ENSACAG00000024787 | ***soat2*** | sterol O-acyltransferase 2 |
| I | ASU_Acar_G.8372 | 100558409 | ENSACAG00000001791 | ***sorbs2*** | sorbin and SH3 domain containing 2 |
| I | ASU_Acar_G.14446 | 100566954 | ENSACAG00000006111 | ***sox8*** | SRY (sex determining region Y)-box 8 |
| I | ASU_Acar_G.1782 | 100562065 | ENSACAG00000013938 | ***speg*** | SPEG complex locus |
| I | ASU_Acar_G.200 | 100551915 | - | ***spnb_x*** | spectrin beta chain X |
| I | ASU_Acar_G.1435 | 100551915 | - | ***sptbn4*** | Spectrin beta chain, brain 4 |
| I | ASU_Acar_G.20936 | 100555634 | ENSACAG00000016380 | ***srl*** | sarcalumenin |
| I | ASU_Acar_G.8265 | - | - | ***stbd1*** | starch binding domain 1 |
| I | ASU_Acar_G.15883 | - | - | ***susd5*** | sushi domain containing 5 |
| I | ASU_Acar_G.17025 | 100553596 | ENSACAG00000004741 | ***sv2b*** | synaptic vesicle glycoprotein 2B |
| I | ASU_Acar_G.16913 | 100554252 | ENSACAG00000008247 | ***synm*** | synemin, intermediate filament protein |
| I | ASU_Acar_G.9304 | 100567593 | ENSACAG00000013437 | ***synpo2*** | synaptopodin 2 |
| I | ASU_Acar_G.12202 | 100567988 | ENSACAG00000009895 | ***synpo2l*** | synaptopodin 2-like |
| I | ASU_Acar_G.6624 | 100562355 | ENSACAG00000010873 | ***sypl2*** | synaptophysin-like 2 |
| I | ASU_Acar_G.13128 | 100559142 | ENSACAG00000008131 | ***tbx15*** | T-box 15 |
| I | ASU_Acar_G.2102 | 100553423 | - | ***tcf15*** | transcription factor 15-like |
| I | ASU_Acar_G.4365 | 100563715 | ENSACAG00000016545 | ***thbs4*** | thrombospondin 4 |
| I | ASU_Acar_G.13864 | 100551819 | ENSACAG00000007270 | ***tmem182*** | transmembrane protein 182 |
| I | ASU_Acar_G.2815 | 100551526 | ENSACAG00000006898 | ***tnnc1*** | troponin C type 1 (slow) |
| I | ASU_Acar_G.10863 | - | - | ***tnnc2*** | troponin C2, fast |
| I | ASU_Acar_G.6954 | 100565698 | ENSACAG00000003672 | ***tnni1*** | troponin I type 1 (skeletal, slow) |
| I | ASU_Acar_G.1753 | 100566786 | - | ***tnni2*** | troponin I type 2 (skeletal, fast) |
| I | ASU_Acar_G.16015 | 100558563 | ENSACAG00000016780 | ***tnnt1*** | troponin T type 1 (skeletal, slow) |
| I | ASU_Acar_G.1632 | 100566207 | ENSACAG00000009622 | ***tnnt3*** | troponin T type 3 (skeletal, fast) |
| I | ASU_Acar_G.3967 | 100552187 | ENSACAG00000014009 | ***tnxb*** | tenascin XB |
| I | ASU_Acar_G.20714 | 100555890 | ENSACAG00000011902 | ***tpm3*** | tropomyosin 3 |
| I | ASU_Acar_G.356 | 100568083 | ENSACAG00000001672 | ***trdn*** | triadin |
| I | ASU_Acar_G.13293 | 100556790 | ENSACAG00000006665 | ***trim72*** | tripartite motif containing 72 |
| I | ASU_Acar_G.20995 | 100566067 | ENSACAG00000009001 | ***try_x*** | one of many trypsin orthologs |
| I | ASU_Acar_G.6121 | - | ENSACAG00000008325 | ***tspear*** | thrombospondin-type laminin G domain and EAR repeats |
| I | ASU_Acar_G.12461 | 100567009 | ENSACAG00000004862 | ***txlnb*** | taxilin beta |
| I | ASU_Acar_G.8938 | 100553379 | ENSACAG00000013827 | ***unc5c*** | unc-5 homolog C (C. elegans) |
| I | ASU_Acar_G.6445 | 100563917 | ENSACAG00000004318 | ***usp13*** | ubiquitin specific peptidase 13 (isopeptidase T-3) |
| I | ASU_Acar_G.1185 | 100557275 | ENSACAG00000006244 | ***vash2*** | vasohibin 2 |
| I | ASU_Acar_G.6904 | 100560843 | - | ***vdhap*** | vitamin D3 hydroxylase-associated protein |
| I | ASU_Acar_G.2431 | 100564041 | ENSACAG00000014238 | ***vgll2*** | vestigial like 2 (Drosophila) |
| I | ASU_Acar_G.10074 | 100563347 | ENSACAG00000013461 | ***vwde*** | von Willebrand factor D and EGF domain-containing protein-like |
| I | ASU_Acar_G.10736 | 100563155 | ENSACAG00000013446 | ***vwde*** | von Willebrand factor D and EGF domain-containing protein-like |
| I | ASU_Acar_G.9773 | - | ENSACAG00000000853 | ***wipf3*** | WAS/WASL interacting protein family, member 3 |
| I | ASU_Acar_G.13393 | 100555022 | ENSACAG00000001150 | ***wnk2*** | WNK lysine deficient protein kinase 2 |
| I | ASU_Acar_G.10333 | 100560977 | ENSACAG00000009771 | ***xirp1*** | xin actin-binding repeat containing 1 |
| I | ASU_Acar_G.13007 | 100559876 | ENSACAG00000000350 | ***xirp2*** | xin actin-binding repeat containing 2 |
| II | ASU_Acar_G.20843 | 100558828 | - | ***aadacl_x*** | arylacetamide deacetylase-like 3 or 4 |
| II | ASU_Acar_G.20840 | 100559422 | ENSACAG00000002416 | ***aadacl4*** | arylacetamide deacetylase-like 4 |
| II | ASU_Acar_G.3691 | 100559712 | ENSACAG00000017629 | ***acer2*** | alkaline ceramidase 2 |
| II | ASU_Acar_G.3327 | 100566345 | ENSACAG00000012229 | ***aldh1a1*** | aldehyde dehydrogenase 1 family, member A1 |
| II | ASU_Acar_G.17179 | 100557840 | ENSACAG00000005188 | ***aloxe3*** | arachidonate lipoxygenase 3 |
| II | ASU_Acar_G.753 | 100559032 | ENSACAG00000010962 | ***aspg*** | asparaginase homolog (S. cerevisiae) |
| II | ASU_Acar_G.21997 | - | - | ***bsg*** | basigin precursor |
| II | ASU_Acar_G.8949 | 100555471 | ENSACAG00000013211 | ***cbfb*** | core-binding factor, beta subunit |
| II | ASU_Acar_G.11779 | 100558911 | ENSACAG00000012480 | ***cdc42*** | cell division cycle 42 |
| II | ASU_Acar_G.12188 | 100560914 | ENSACAG00000000244 | ***cdh9*** | cadherin 9, type 2 (T1-cadherin) |
| II | ASU_Acar_G.467 | 100557664 | ENSACAG00000015229 | ***cga*** | glycoprotein hormones, alpha polypeptide |
| II | ASU_Acar_G.7506 | 100559528 | ENSACAG00000003458 | ***chi3l1*** | chitinase-3-like 1 |
| II | ASU_Acar_G.11771 | - | ENSACAG00000026507 | ***chit1*** | chitinase 1 (chitotriosidase) |
| II | ASU_Acar_G.5366 | 100556182 | ENSACAG00000021113 | ***cldn17*** | claudin 17 |
| II | ASU_Acar_G.15269 | 100563233 | ENSACAG00000006661 | ***corin*** | corin, serine peptidase |
| II | ASU_Acar_G.4036 | 100566148 | ENSACAG00000012108 | ***cyp1a4*** | cytochrome P450 1A4 |
| II | ASU_Acar_G.9653 | 100558940 | - | ***cyp2d14*** | cytochrome P450 2D14 |
| II | ASU_Acar_G.9964 | 100564794 | ENSACAG00000008272 | ***ddc*** | dopa decarboxylase (aromatic L-amino acid decarboxylase) |
| II | ASU_Acar_G.2039 | 100553484 | ENSACAG00000017365 | ***dio2*** | deiodinase, iodothyronine, type II |
| II | ASU_Acar_G.16937 | 100557586 | ENSACAG00000008330 | ***dkk2*** | dickkopf 2 homolog (Xenopus laevis) |
| II | ASU_Acar_G.12104 | 100552794 | ENSACAG00000005782 | ***dnase2b*** | deoxyribonuclease II beta |
| II | ASU_Acar_G.19196 | 100565931 | ENSACAG00000003712 | ***edn3*** | endothelin 3 |
| II | ASU_Acar_G.8260 | 100559070 | ENSACAG00000003899 | ***ednra*** | endothelin receptor type A |
| II | ASU_Acar_G.15234 | 100559940 | ENSACAG00000011588 | ***emilin3*** | elastin microfibril interfacer 3 |
| II | ASU_Acar_G.5667 | 100553564 | ENSACAG00000004301 | ***entpd1*** | ectonucleoside triphosphate diphosphohydrolase 1 |
| II | ASU_Acar_G.3760 | 100566666 | ENSACAG00000007987 | ***f2r*** | coagulation factor II (thrombin) receptor |
| II | ASU_Acar_G.3789 | 100566862 | ENSACAG00000015211 | ***fam19a1*** | family with sequence similarity 19 (chemokine (C-C motif)-like), member A1 |
| II | ASU_Acar_G.8846 | 100555664 | ENSACAG00000014456 | ***fbln1*** | fibulin 1 |
| II | ASU_Acar_G.4735 | 100554153 | ENSACAG00000005410 | ***fgfr4*** | fibroblast growth factor receptor 4 |
| II | ASU_Acar_G.1063 | 100565351 | - | **G.1063** | predicted lncRNA |
| II | ASU_Acar_G.10886 | - | - | **G.10886** | predicted ncRNA |
| II | ASU_Acar_G.13829 | - | - | **G.13829** | predicted ncRNA |
| II | ASU_Acar_G.14791 | - | - | **G.14791** | predicted ncRNA |
| II | ASU_Acar_G.1721 | - | - | **G.1721** | predicted ncRNA |
| II | ASU_Acar_G.17546 | - | - | **G.17546** | predicted lncRNA |
| II | ASU_Acar_G.17964 | - | - | **G.17964** | predicted lncRNA |
| II | ASU_Acar_G.18922 | 100560994 | - | ***cd298l2*** | cd209 antigen-like protein 2 |
| II | ASU_Acar_G.19198 | - | - | **G.19198** | unknown protein-coding |
| II | ASU_Acar_G.20175 | - | - | **G.20175** | predicted lncRNA |
| II | ASU_Acar_G.21065 | - | ENSACAG00000025572 | ***ccer2*** | coiled-coil glutamate-rich protein 2 |
| II | ASU_Acar_G.2935 | - | - | **G.2935** | unknown protein-coding |
| II | ASU_Acar_G.3586 | - | - | **G.3586** | predicted ncRNA |
| II | ASU_Acar_G.5235 | 100564051 | - | **G.5235** | predicted lncRNA |
| II | ASU_Acar_G.8944 | - | - | **G.8944** | predicted lncRNA |
| II | ASU_Acar_G.15461 | 100560533 | ENSACAG00000004532 | ***gfra4*** | GDNF family receptor alpha 4 |
| II | ASU_Acar_G.4243 | 100551985 | ENSACAG00000003374 | ***glis3*** | GLIS family zinc finger 3 |
| II | ASU_Acar_G.18858 | 100551831 | ENSACAG00000016607 | ***gmfb*** | glia maturation factor, beta |
| II | ASU_Acar_G.11436 | - | ENSACAG00000011526 | ***gosr1*** | golgi SNAP receptor complex member 1 |
| II | ASU_Acar_G.9716 | 100566037 | ENSACAG00000008498 | ***gpr158*** | G protein-coupled receptor 158 |
| II | ASU_Acar_G.5346 | 100551602 | ENSACAG00000009972 | ***gramd1c*** | GRAM domain containing 1C |
| II | ASU_Acar_G.1712 | 100562335 | ENSACAG00000000267 | ***greb1*** | growth regulation by estrogen in breast cancer 1 |
| II | ASU_Acar_G.11121 | 100556620 | ENSACAG00000010658 | ***hbad*** | Hemoglobin subunit alpha-D |
| II | ASU_Acar_G.21468 | 100552694 | ENSACAG00000012173 | ***hbb-b1*** | hemoglobin subunit beta-1 |
| II | ASU_Acar_G.5347 | 100558532 | ENSACAG00000014257 | ***hephl1*** | hephaestin-like 1 |
| II | ASU_Acar_G.435 | 100560623 | ENSACAG00000002130 | ***hhipl2*** | HHIP-like 2 |
| II | ASU_Acar_G.16514 | 100563488 | - | ***hsd17b14*** | 17-beta-hydroxysteroid dehydrogenase 14 |
| II | ASU_Acar_G.10506 | 100568251 | ENSACAG00000016160 | ***igfbp4*** | insulin-like growth factor binding protein 4 |
| II | ASU_Acar_G.14777 | - | ENSACAG00000012548 | ***il17re*** | interleukin-17 receptor e |
| II | ASU_Acar_G.13871 | 100553004 | ENSACAG00000007454 | ***il1r1*** | interleukin-1 receptor type 1 |
| II | ASU_Acar_G.22991 | 100563808 | ENSACAG00000017868 | ***krt19*** | keratin 19 |
| II | ASU_Acar_G.22997 | 100553851 | - | ***krt42*** | keratin 42 |
| II | ASU_Acar_G.16728 | 100359375 | ENSACAG00000027236 | ***li-ac-12*** | Keratin-associated beta-protein 12 |
| II | ASU_Acar_G.16727 | 100359377 | ENSACAG00000007609 | ***li-ac-14*** | Keratin-associated beta-protein 14 |
| II | ASU_Acar_G.16695 | 100379173 | ENSACAG00000025169 | ***li-ac-17*** | Keratin-associated beta-protein 17 |
| II | ASU_Acar_G.16706 | 100554184 | ENSACAG00000007588 | ***li-ac-5*** | Keratin-associated beta-protein 5 |
| II | ASU_Acar_G.16692 | 100555160 | - | ***li-ac-x*** | Keratin-associated beta-protein X |
| II | ASU_Acar_G.16694 | 100558368 | ENSACAG00000007612 | ***li-ac-x*** | Keratin-associated beta-protein X |
| II | ASU_Acar_G.16714 | 100557315 | ENSACAG00000007603 | ***li-ac-x*** | Keratin-associated beta-protein X |
| II | ASU_Acar_G.16722 | 100558565 | ENSACAG00000007613 | ***li-ac-x*** | Keratin-associated beta-protein X |
| II | ASU_Acar_G.18499 | - | - | ***limx1b*** | lim homeobox transcription factor 1-beta isoform 1 |
| II | ASU_Acar_G.7795 | 100567585 | ENSACAG00000003883 | ***lppr4*** | lipid phosphate phosphatase-related protein type 4 |
| II | ASU_Acar_G.15467 | 100562102 | ENSACAG00000003555 | ***lypd6b*** | LY6/PLAUR domain containing 6B |
| II | ASU_Acar_G.16715 | 100559746 | - | ***maml2*** | mastermind-like protein 2-like |
| II | ASU_Acar_G.36 | - | - | ***mboat2*** | membrane bound O-acyltransferase domain containing 2 |
| II | ASU_Acar_G.141 | 100565875 | ENSACAG00000004140 | ***mdk*** | midkine (neurite growth-promoting factor 2) |
| II | ASU_Acar_G.8828 | - | - | ***mepe*** | extracelular matrix phosphoglycoprotein |
| II | ASU_Acar_G.5463 | 100552848 | ENSACAG00000012632 | ***mmp1*** | matrix metallpeptidase 1 (interstitial collagenase) |
| II | ASU_Acar_G.6293 | - | ENSACAG00000006009 | ***mxi1*** | MAX interactor 1, dimerization protein |
| II | ASU_Acar_G.10610 | 100565835 | ENSACAG00000008363 | ***myo3a*** | myosin IIIA |
| II | ASU_Acar_G.9462 | 100566168 | ENSACAG00000012994 | ***ndnf*** | neuron-derived neurotrophic factor |
| II | ASU_Acar_G.8400 | 100556249 | ENSACAG00000002584 | ***nell2*** | NEL-like 2 (chicken) |
| II | ASU_Acar_G.10755 | 100562562 | ENSACAG00000011889 | ***or5v1*** | olfactory receptor 5V1 |
| II | ASU_Acar_G.13725 | 100566043 | ENSACAG00000004288 | ***ovch2*** | ovochymase 2 |
| II | ASU_Acar_G.5371 | 100558401 | - | ***pcp4*** | Purkinje cell protein 4 |
| II | ASU_Acar_G.2989 | 100565158 | ENSACAG00000012731 | ***pcsk1*** | proprotein convertase subtilisin/kexin type 1 |
| II | ASU_Acar_G.8714 | 100568114 | ENSACAG00000016828 | ***pdgfc*** | platelet derived growth factor C |
| II | ASU_Acar_G.9594 | 100565834 | ENSACAG00000010297 | ***pdgfra*** | platelet-derived growth factor receptor, alpha polypeptide |
| II | ASU_Acar_G.5662 | 100555200 | ENSACAG00000012419 | ***pir*** | pirin (iron-binding nuclear protein) |
| II | ASU_Acar_G.840 | 100563319 | ENSACAG00000013175 | ***pla2g7*** | phospholipase A2, group VII (platelet-activating factor acetylhydrolase, plasma) |
| II | ASU_Acar_G.9193 | 100564195 | - | ***plac8*** | placenta-specific gene 8 protein |
| II | ASU_Acar_G.624 | 100556951 | ENSACAG00000004285 | ***prss35*** | protease, serine, 35 |
| II | ASU_Acar_G.21983 | - | ENSACAG00000010835 | ***psmg4*** | proteasome (prosome, macropain) assembly chaperone 4 |
| II | ASU_Acar_G.3775 | 100561490 | ENSACAG00000004851 | ***ptk7*** | protein tyrosine kinase 7 |
| II | ASU_Acar_G.7245 | 100568241 | ENSACAG00000000141 | ***ptpn22*** | protein tyrosine phosphatase, non-receptor type 22 (lymphoid) |
| II | ASU_Acar_G.19453 | 100567421 | - | ***ptprq*** | phosphotidylinositol phosphatase ptprq |
| II | ASU_Acar_G.5620 | 100557939 | ENSACAG00000006780 | ***ptx3*** | pentraxin 3, long |
| II | ASU_Acar_G.22381 | - | ENSACAG00000003740 | ***rab8b*** | RAB8B, member RAS oncogene family |
| II | ASU_Acar_G.15277 | 100562835 | ENSACAG00000005848 | ***rasl11b*** | RAS-like, family 11, member B |
| II | ASU_Acar_G.13182 | 100555482 | ENSACAG00000007894 | ***rnase_x*** | one of many ribonuclease orthologs |
| II | ASU_Acar_G.3015 | 100554214 | ENSACAG00000000922 | ***ror2*** | receptor tyrosine kinase-like orphan receptor 2 |
| II | ASU_Acar_G.4535 | - | - | ***rps28*** | 40s ribosomal protein s28 |
| II | ASU_Acar_G.13441 | 100559539 | ENSACAG00000014399 | ***sall1*** | sal-like 1 (Drosophila) |
| II | ASU_Acar_G.13416 | 100557376 | ENSACAG00000012748 | ***sall4*** | sal-like 4 (Drosophila) |
| II | ASU_Acar_G.8250 | 100557427 | ENSACAG00000015808 | ***scube1*** | signal peptide, CUB domain, EGF-like 1 |
| II | ASU_Acar_G.19817 | 100557132 | - | ***sdr16c5*** | short chain dehydrogenase/reductase family 16C, member 5 |
| II | ASU_Acar_G.10785 | 100553546 | ENSACAG00000008817 | ***selenbp1*** | selenium binding protein 1 |
| II | ASU_Acar_G.19037 | 100562580 | ENSACAG00000008067 | ***selenbp1*** | selenium binding protein 1 |
| II | ASU_Acar_G.14005 | 100556598 | ENSACAG00000017642 | ***serpinb_x*** | serpin peptidase inhibitor, clade B (ovalbumin), member X |
| II | ASU_Acar_G.3544 | - | - | ***slc1a1*** | solute carrier family 1 (neuronal/epithelial high affinity glutamate transporter, system Xag), member 1 |
| II | ASU_Acar_G.10604 | 100562367 | ENSACAG00000011641 | ***slc27a2*** | solute carrier family 27 (fatty acid transporter), member 2 |
| II | ASU_Acar_G.9982 | 100563543 | ENSACAG00000017228 | ***slc4a1*** | solute carrier family 4, anion exchanger, member 1 (erythrocyte membrane protein band 3, Diego blood group) |
| II | ASU_Acar_G.2638 | 100555912 | ENSACAG00000009875 | ***slc6a11*** | solute carrier family 6 (neurotransmitter transporter, GABA), member 11 |
| II | ASU_Acar_G.17778 | 100556664 | ENSACAG00000010309 | ***spon2*** | spondin 2, extracellular matrix protein |
| II | ASU_Acar_G.9105 | 100562556 | ENSACAG00000012670 | ***spp1*** | secreted phosphoprotein 1 |
| II | ASU_Acar_G.5738 | 100529108 | ENSACAG00000002478 | ***st6gal2*** | ST6 beta-galactosamide alpha-2,6-sialyltranferase 2 |
| II | ASU_Acar_G.10555 | - | - | ***stk17a*** | serine/threonine kinase 17a |
| II | ASU_Acar_G.432 | 100554924 | ENSACAG00000013607 | ***syt12*** | synaptotagmin XII |
| II | ASU_Acar_G.902 | 100561541 | ENSACAG00000009753 | ***th*** | tyrosine hydroxylase |
| II | ASU_Acar_G.22339 | 100557208 | ENSACAG00000007487 | ***thy1*** | Thy-1 cell surface antigen |
| II | ASU_Acar_G.10734 | 100563348 | ENSACAG00000013101 | ***tmem98*** | transmembrane protein 98 |
| II | ASU_Acar_G.10108 | 100565708 | ENSACAG00000013144 | ***tmprss11b*** | transmembrane protease, serine 11B |
| II | ASU_Acar_G.22995 | 100337544 | ENSACAG00000017876 | ***tmprss11e*** | transmembrane protease, serine 11E |
| II | ASU_Acar_G.14579 | 100565448 | ENSACAG00000008445 | ***tnfrsf11b*** | tumor necrosis factor receptor superfamily, member 11b |
| II | ASU_Acar_G.10429 | 100564923 | ENSACAG00000012640 | ***tspan13*** | tetraspanin 13 |
| II | ASU_Acar_G.9821 | 100379171 | ENSACAG00000011825 | ***twist1*** | twist homolog 1 |
| II | ASU_Acar_G.8348 | 100553712 | ENSACAG00000003473 | ***wif1*** | WNT inhibitory factor 1 |
| II | ASU_Acar_G.9450 | 100553645 | ENSACAG00000006979 | ***wnt16*** | wingless-type MMTV integration site family, member 16 |
| II | ASU_Acar_G.3955 | 100555653 | ENSACAG00000017453 | ***wnt5a*** | wingless-type MMTV integration site family, member 5A |
| II | ASU_Acar_G.892 | - | - | ***znf135*** | zinc finger protein 135 |
