# Supplementary material for: Transcriptomic Analysis of Tail Regeneration in the Lizard Anolis carolinensis Reveals Activation of Conserved Vertebrate Developmental and Repair Mechanisms
Source: PLoS One. 2014 Aug 20;9(8):e105004. doi: 10.1371/journal.pone.0105004 (PMC4139331; doi:10.1371/journal.pone.0105004)
Supplement: Table S4 — Differentially expressed genes in the lizard regenerating tail at 25 dpa analyzed by DESeq2. (DOCX) [file pone.0105004.s009.docx]

| **Table S4. Differentially expressed genes in the lizard regenerating tail at 25 dpa analyzed by  DESeq2.** | | | | | | | |
| --- | --- | --- | --- | --- | --- | --- | --- |
| **Cluster** | **Gene** | **NCBI_ID** | **ensembl_ID** | **Ortholog** | **Orthologous Gene Description** |  |  |
| I | ASU_Acar_G.13672 | 100560196 | ENSACAG00000011592 | ***ablim2*** | actin binding LIM protein family, member 2 |  |  |
| I | ASU_Acar_G.21712 | 100554326 | ENSACAG00000013375 | ***acan*** | aggrecan |  |  |
| I | ASU_Acar_G.1152 | 100566789 | ENSACAG00000000836 | ***acta1*** | actin, alpha 1, skeletal muscle |  |  |
| I | ASU_Acar_G.20111 | 100566451 | ENSACAG00000012464 | ***actc1*** | actin, alpha, cardiac muscle 1 |  |  |
| I | ASU_Acar_G.12270 | 100557764 | ENSACAG00000017722 | ***adamts15*** | ADAM metallopeptidase with thrombospondin type 1 motif, 15 |  |  |
| I | ASU_Acar_G.15610 | 100566047 | ENSACAG00000010688 | ***aff2*** | AF4/FMR2 family, member 2 |  |  |
| I | ASU_Acar_G.4813 | 100555454 | ENSACAG00000002643 | ***akap6*** | A kinase (PRKA) anchor protein 6 |  |  |
| I | ASU_Acar_G.18361 | - | ENSACAG00000006448 | ***alpk3*** | alpha-kinase 3 |  |  |
| I | ASU_Acar_G.7007 | 100565176 | ENSACAG00000003090 | ***ampd1*** | adenosine monophosphate deaminase 1 |  |  |
| I | ASU_Acar_G.7188 | 100554228 | ENSACAG00000016506 | ***apobec2*** | apolipoprotein B mRNA editing enzyme, catalytic polypeptide-like 2 |  |  |
| I | ASU_Acar_G.9218 | 100559860 | ENSACAG00000016586 | ***apol_X*** | apolipoprotein L, X |  |  |
| I | ASU_Acar_G.21026 | 100552891 | ENSACAG00000003014 | ***asb12*** | ankyrin repeat and SOCS box containing 12 |  |  |
| I | ASU_Acar_G.11972 | 100554142 | - | ***atp1a2*** | ATPase, Na+/K+ transporting, alpha 2 polypeptide |  |  |
| I | ASU_Acar_G.22415 | 100554397 | ENSACAG00000015855 | ***atp1a2*** | ATPase, Na+/K+ transporting, alpha 2 polypeptide |  |  |
| I | ASU_Acar_G.21914 | 100568151 | ENSACAG00000001902 | ***baiap2l1*** | BAI1-associated protein 2-like 1 |  |  |
| I | ASU_Acar_G.2894 | 100558455 | ENSACAG00000016768 | ***bhmt*** | betaine--homocysteine S-methyltransferase |  |  |
| I | ASU_Acar_G.15385 | - | ENSACAG00000025734 | ***c10orf71*** | chromosome 10 open reading frame 71 |  |  |
| I | ASU_Acar_G.10230 | 100564526 | ENSACAG00000017422 | ***cacnb1*** | calcium channel, voltage-dependent, beta 1 subunit |  |  |
| I | ASU_Acar_G.7334 | 100564119 | ENSACAG00000003976 | ***cap2*** | CAP, adenylate cyclase-associated protein, 2 (yeast) |  |  |
| I | ASU_Acar_G.21481 | 100554653 | ENSACAG00000015755 | ***casq1*** | calsequestrin 1 (fast-twitch, skeletal muscle) |  |  |
| I | ASU_Acar_G.4130 | 100566014 | ENSACAG00000003187 | ***casq2*** | calsequestrin 2 (cardiac muscle) |  |  |
| I | ASU_Acar_G.4712 | 100562999 | ENSACAG00000011878 | ***cav2*** | caveolin 2 |  |  |
| I | ASU_Acar_G.4348 | 100552705 | ENSACAG00000011920 | ***cav3*** | caveolin 3 |  |  |
| I | ASU_Acar_G.18303 | 100551698 | ENSACAG00000016950 | ***cdh15*** | cadherin 15, type 1, M-cadherin (myotubule) |  |  |
| I | ASU_Acar_G.4064 | 100553103 | ENSACAG00000002385 | ***cfl2*** | cofilin 2 (muscle) |  |  |
| I | ASU_Acar_G.20626 | 100560885 | ENSACAG00000004276 | ***chd3*** | chromodomain helicase DNA binding protein 3 |  |  |
| I | ASU_Acar_G.16830 | 100557909 | ENSACAG00000005293 | ***chrna1*** | cholinergic receptor, nicotinic, alpha 1 (muscle) |  |  |
| I | ASU_Acar_G.22238 | 100554201 | ENSACAG00000014790 | ***chrne*** | cholinergic receptor, nicotinic, epsilon (muscle) |  |  |
| I | ASU_Acar_G.22561 | 100562721 | ENSACAG00000005396 | ***ckm*** | creatine kinase, muscle |  |  |
| I | ASU_Acar_G.20941 | 100557792 | ENSACAG00000003040 | ***col11a2*** | collagen, type XI, alpha 2 |  |  |
| I | ASU_Acar_G.5946 | - | ENSACAG00000017768 | ***col13a1*** | collagen, type XIII, alpha 1 |  |  |
| I | ASU_Acar_G.18867 | 100553016 | ENSACAG00000006064 | ***col2a1*** | collagen, type II, alpha 1 |  |  |
| I | ASU_Acar_G.987 | 100567245 | ENSACAG00000008314 | ***col9a1*** | collagen, type IX, alpha 1 |  |  |
| I | ASU_Acar_G.13236 | 100557181 | - | ***cox6a2*** | cytochrome c oxidase subunit VIa polypeptide 2 |  |  |
| I | ASU_Acar_G.116 | 100566206 | ENSACAG00000015769 | ***csrp3*** | cysteine and glycine-rich protein 3 (cardiac LIM protein) |  |  |
| I | ASU_Acar_G.3682 | 100560236 | ENSACAG00000008394 | ***cygb*** | cytoglobin |  |  |
| I | ASU_Acar_G.8215 | 100559393 | ENSACAG00000011024 | ***dennd2a*** | DENN/MADD domain containing 2A |  |  |
| I | ASU_Acar_G.7854 | 100555207 | ENSACAG00000005686 | ***deptor*** | DEP domain containing MTOR-interacting protein |  |  |
| I | ASU_Acar_G.2218 | 100560815 | ENSACAG00000013847 | ***des*** | desmin |  |  |
| I | ASU_Acar_G.21318 | 100564621 | ENSACAG00000004019 | ***dmpk*** | dystrophia myotonica-protein kinase |  |  |
| I | ASU_Acar_G.12798 | 100561061 | ENSACAG00000006911 | ***dtna*** | dystrobrevin, alpha |  |  |
| I | ASU_Acar_G.2919 | - | ENSACAG00000009767 | ***ecm2*** | extracellular matrix protein 2 |  |  |
| I | ASU_Acar_G.6665 | 100561171 | ENSACAG00000006007 | ***eef1a2*** | eukaryotic translation elongation factor 1 alpha 2 |  |  |
| I | ASU_Acar_G.6389 | 100563528 | ENSACAG00000001659 | ***egfl6*** | EGF-like-domain, multiple 6 |  |  |
| I | ASU_Acar_G.18083 | 100555562 | ENSACAG00000006404 | ***fbxo40*** | F-box protein 40 |  |  |
| I | ASU_Acar_G.1547 | 100564828 | ENSACAG00000016070 | ***fgf4*** | fibroblast growth factor 4 |  |  |
| I | ASU_Acar_G.21948 | 100555896 | ENSACAG00000009029 | ***flnb*** | filamin B, beta |  |  |
| I | ASU_Acar_G.21857 | 100559302 | ENSACAG00000009986 | ***flnc*** | filamin C, gamma |  |  |
| I | ASU_Acar_G.17240 | - | - | ***fyttd1*** | forty-two-three domain containing 1 |  |  |
| I | ASU_Acar_G.5031 | 100565823 | ENSACAG00000002739 | ***fzd4*** | frizzled family receptor 4 |  |  |
| I | ASU_Acar_G.11714 | - | - | **G.11714** | unknown protein-coding |  |  |
| I | ASU_Acar_G.15352 | - | - | **G.15352** | unknown noncoding |  |  |
| I | ASU_Acar_G.15880 | - | - | **G.15880** | unknown protein-coding |  |  |
| I | ASU_Acar_G.16280 | - | - | **G.16280** | unknown protein-coding |  |  |
| I | ASU_Acar_G.18812 | - | - | **G.18812** | unknown protein-coding |  |  |
| I | ASU_Acar_G.2905 | - | - | **G.2905** | unknown noncoding |  |  |
| I | ASU_Acar_G.4963 | - | - | **G.4963** | unknown noncoding |  |  |
| I | ASU_Acar_G.7180 | - | - | **G.7180** | unknown protein-coding |  |  |
| I | ASU_Acar_G.9048 | - | - | **G.9048** | unknown protein-coding |  |  |
| I | ASU_Acar_G.11418 | 100560810 | ENSACAG00000004389 | ***gatm*** | glycine amidinotransferase (L-arginine:glycine amidinotransferase) |  |  |
| I | ASU_Acar_G.5392 | 100564117 | ENSACAG00000001831 | ***gpm6b*** | glycoprotein M6B |  |  |
| I | ASU_Acar_G.3693 | 100555587 | ENSACAG00000011432 | ***hapln1*** | hyaluronan and proteoglycan link protein 1 |  |  |
| I | ASU_Acar_G.21371 | 100556479 | ENSACAG00000016435 | ***hfe2*** | hemochromatosis type 2 (juvenile) |  |  |
| I | ASU_Acar_G.10140 | 100564922 | ENSACAG00000006760 | ***hhatl*** | hedgehog acyltransferase-like |  |  |
| I | ASU_Acar_G.19486 | 100557786 | ENSACAG00000002673 | ***hspb7*** | heat shock 27kDa protein family, member 7 (cardiovascular) |  |  |
| I | ASU_Acar_G.12359 | 100552402 | ENSACAG00000010030 | ***itga7*** | integrin, alpha 7 |  |  |
| I | ASU_Acar_G.7938 | 100562750 | ENSACAG00000016733 | ***jph2*** | junctophilin 2 |  |  |
| I | ASU_Acar_G.16745 | 100564938 | ENSACAG00000013024 | ***kif24*** | kinesin family member 24 |  |  |
| I | ASU_Acar_G.21385 | - | ENSACAG00000010805 | ***klhl20*** | kelch-like family member 20 |  |  |
| I | ASU_Acar_G.5785 | 100566093 | ENSACAG00000006547 | ***klhl30*** | kelch-like family member 30 |  |  |
| I | ASU_Acar_G.1581 | 100551911 | ENSACAG00000008404 | ***klhl31*** | kelch-like family member 31 |  |  |
| I | ASU_Acar_G.18458 | 100564817 | ENSACAG00000005097 | ***klhl31*** | kelch-like family member 31 |  |  |
| I | ASU_Acar_G.4422 | 100562869 | ENSACAG00000013350 | ***lamb2*** | laminin, beta 2 (laminin S) |  |  |
| I | ASU_Acar_G.9579 | 100553447 | ENSACAG00000012474 | ***lgals1*** | lectin, galactoside-binding, soluble, 1 |  |  |
| I | ASU_Acar_G.5029 | 100556575 | ENSACAG00000001970 | ***map3k7cl*** | MAP3K7 C-terminal like |  |  |
| I | ASU_Acar_G.8050 | 100560579 | ENSACAG00000012284 | ***matn4*** | matrilin 4 |  |  |
| I | ASU_Acar_G.4723 | 100559834 | ENSACAG00000000488 | ***mef2c*** | myocyte enhancer factor 2C |  |  |
| I | ASU_Acar_G.6212 | 100555258 | ENSACAG00000015543 | ***megf6*** | multiple EGF-like-domains 6 |  |  |
| I | ASU_Acar_G.15165 | 100562636 | ENSACAG00000007087 | ***mfap4*** | microfibrillar-associated protein 4 |  |  |
| I | ASU_Acar_G.10180 | 100563668 | ENSACAG00000007829 | ***mkx*** | mohawk homeobox |  |  |
| I | ASU_Acar_G.7373 | 100556384 | ENSACAG00000014585 | ***msc*** | musculin |  |  |
| I | ASU_Acar_G.2697 | 100557219 | ENSACAG00000004208 | ***mstn*** | myostatin |  |  |
| I | ASU_Acar_G.8132 | 100562221 | ENSACAG00000001796 | ***murc*** | muscle-related coiled-coil protein |  |  |
| I | ASU_Acar_G.19997 | 100567683 | ENSACAG00000006296 | ***myh6*** | myosin, heavy chain 6, cardiac muscle, alpha |  |  |
| I | ASU_Acar_G.387 | 100558249 | ENSACAG00000010643 | ***myl1*** | myosin, light chain 1, alkali; skeletal, fast |  |  |
| I | ASU_Acar_G.19536 | 100561920 | ENSACAG00000015270 | ***myl10*** | myosin, light chain 10, regulatory |  |  |
| I | ASU_Acar_G.17474 | 100566191 | ENSACAG00000002200 | ***myl2*** | myosin, light chain 2, regulatory, cardiac, slow |  |  |
| I | ASU_Acar_G.17789 | 100557845 | ENSACAG00000005125 | ***myl3*** | myosin, light chain 3, alkali; ventricular, skeletal, slow |  |  |
| I | ASU_Acar_G.10195 | 100558684 | ENSACAG00000017407 | ***myl4*** | myosin, light chain 4, alkali; atrial, embryonic |  |  |
| I | ASU_Acar_G.3409 | 100561943 | ENSACAG00000022592 | ***myl6b*** | myosin, light chain 6B, alkali, smooth muscle and non-muscle |  |  |
| I | ASU_Acar_G.15644 | 100567803 | ENSACAG00000011501 | ***mylk4*** | myosin light chain kinase family, member 4 |  |  |
| I | ASU_Acar_G.18654 | 100559161 | ENSACAG00000010684 | ***mylpf*** | myosin light chain, phosphorylatable, fast skeletal muscle |  |  |
| I | ASU_Acar_G.11107 | - | - | ***myo18b*** | myosin XVIIIB |  |  |
| I | ASU_Acar_G.11108 | - | - | ***myo18b*** | myosin XVIIIB |  |  |
| I | ASU_Acar_G.11610 | - | ENSACAG00000011529 | ***myo18b*** | myosin XVIIIB |  |  |
| I | ASU_Acar_G.7164 | 100561042 | ENSACAG00000004818 | ***myog*** | myogenin (myogenic factor 4) |  |  |
| I | ASU_Acar_G.6620 | 100565825 | ENSACAG00000009246 | ***myom1*** | myomesin 1 |  |  |
| I | ASU_Acar_G.9302 | 100567139 | ENSACAG00000013422 | ***myoz2*** | myozenin 2 |  |  |
| I | ASU_Acar_G.15473 | 100563097 | ENSACAG00000003769 | ***neb*** | nebulin |  |  |
| I | ASU_Acar_G.20295 | 100560408 | ENSACAG00000017577 | ***net1*** | neuroepithelial cell transforming 1 |  |  |
| I | ASU_Acar_G.6606 | 100565300 | ENSACAG00000000941 | ***nfatc1*** | nuclear factor of activated T-cells, cytoplasmic, calcineurin-dependent 1 |  |  |
| I | ASU_Acar_G.16788 | 100551695 | ENSACAG00000008437 | ***npnt*** | nephronectin |  |  |
| I | ASU_Acar_G.10740 | 100558091 | ENSACAG00000007504 | ***npsr1*** | neuropeptide S receptor 1 |  |  |
| I | ASU_Acar_G.3230 | 100559576 | ENSACAG00000016775 | ***nr4a1*** | nuclear receptor subfamily 4, group A, member 1 |  |  |
| I | ASU_Acar_G.6874 | 100554424 | ENSACAG00000016516 | ***oard1*** | O-acyl-ADP-ribose deacylase 1 |  |  |
| I | ASU_Acar_G.10284 | 100566306 | ENSACAG00000006865 | ***obscn*** | obscurin, cytoskeletal calmodulin and titin-interacting RhoGEF |  |  |
| I | ASU_Acar_G.18419 | 100557783 | ENSACAG00000017955 | ***pappa*** | pregnancy-associated plasma protein A, pappalysin 1 |  |  |
| I | ASU_Acar_G.11956 | 100553158 | - | ***pax7*** | paired box 7 |  |  |
| I | ASU_Acar_G.7486 | 100559258 | ENSACAG00000013127 | ***pde4dip*** | phosphodiesterase 4D interacting protein |  |  |
| I | ASU_Acar_G.13772 | 100567602 | ENSACAG00000002010 | ***pgam2*** | phosphoglycerate mutase 2 (muscle) |  |  |
| I | ASU_Acar_G.20944 | 100558975 | ENSACAG00000000559 | ***pitx3*** | paired-like homeodomain 3 |  |  |
| I | ASU_Acar_G.16672 | 100567874 | ENSACAG00000000635 | ***pknox2*** | PBX/knotted 1 homeobox 2 |  |  |
| I | ASU_Acar_G.7606 | - | - | ***prkaa2*** | protein kinase, AMP-activated, alpha 2 catalytic subunit |  |  |
| I | ASU_Acar_G.22394 | 100566844 | ENSACAG00000012295 | ***pvalb*** | parvalbumin |  |  |
| I | ASU_Acar_G.16242 | 100558891 | ENSACAG00000003680 | ***pxn*** | paxillin |  |  |
| I | ASU_Acar_G.22145 | 100553610 | ENSACAG00000005131 | ***pygm*** | phosphorylase, glycogen, muscle |  |  |
| I | ASU_Acar_G.7048 | 100563604 | ENSACAG00000008983 | ***raly*** | RALY heterogeneous nuclear ribonucleoprotein |  |  |
| I | ASU_Acar_G.10175 | 100563157 | ENSACAG00000011931 | ***rbfox2*** | RNA binding protein, fox-1 homolog (C. elegans) 2 |  |  |
| I | ASU_Acar_G.19788 | 100551835 | ENSACAG00000008872 | ***rpl3l*** | ribosomal protein L3-like |  |  |
| I | ASU_Acar_G.10808 | 100559227 | ENSACAG00000007451 | ***rtn2*** | reticulon 2 |  |  |
| I | ASU_Acar_G.11790 | - | - | ***ryr1*** | ryanodine receptor 1 (skeletal) |  |  |
| I | ASU_Acar_G.17542 | 100558429 | ENSACAG00000001965 | ***ryr1*** | ryanodine receptor 1 (skeletal) |  |  |
| I | ASU_Acar_G.18852 | 100568269 | ENSACAG00000016355 | ***samd4a*** | sterile alpha motif domain containing 4A |  |  |
| I | ASU_Acar_G.18854 | 100568269 | ENSACAG00000016355 | ***samd4a*** | sterile alpha motif domain containing 4A |  |  |
| I | ASU_Acar_G.18813 | 100565207 | ENSACAG00000009159 | ***scn5a*** | sodium channel, voltage-gated, type V, alpha subunit |  |  |
| I | ASU_Acar_G.3087 | 100563519 | ENSACAG00000016493 | ***serinc5*** | serine incorporator 5 |  |  |
| I | ASU_Acar_G.9126 | 100563804 | ENSACAG00000015748 | ***sfrp2*** | secreted frizzled-related protein 2 |  |  |
| I | ASU_Acar_G.19838 | 100561082 | - | ***slc35f2*** | solute carrier family 35, member F2 |  |  |
| I | ASU_Acar_G.22098 | 100561733 | ENSACAG00000024343 | ***slit3*** | slit homolog 3 (Drosophila) |  |  |
| I | ASU_Acar_G.2023 | 100563775 | ENSACAG00000003213 | ***smoc2*** | SPARC related modular calcium binding 2 |  |  |
| I | ASU_Acar_G.5699 | 100560245 | ENSACAG00000016732 | ***smpx*** | small muscle protein, X-linked |  |  |
| I | ASU_Acar_G.317 | 100557408 | ENSACAG00000007205 | ***smtnl1*** | smoothelin-like 1 |  |  |
| I | ASU_Acar_G.10684 | 100561699 | ENSACAG00000008484 | ***smyd1*** | SET and MYND domain containing 1 |  |  |
| I | ASU_Acar_G.4165 | 100567184 | ENSACAG00000024787 | ***soat2*** | sterol O-acyltransferase 2 |  |  |
| I | ASU_Acar_G.14446 | 100566954 | ENSACAG00000006111 | ***sox8*** | SRY (sex determining region Y)-box 8 |  |  |
| I | ASU_Acar_G.1782 | 100562065 | ENSACAG00000013938 | ***speg*** | SPEG complex locus |  |  |
| I | ASU_Acar_G.200 | 100551915 | - | ***spnb_x*** | spectrin beta chain X |  |  |
| I | ASU_Acar_G.1435 | 100551915 | - | ***sptbn4*** | spectrin, beta, non-erythrocytic 4 |  |  |
| I | ASU_Acar_G.20936 | 100555634 | ENSACAG00000016380 | ***srl*** | sarcalumenin |  |  |
| I | ASU_Acar_G.12202 | 100567988 | ENSACAG00000009895 | ***synpo2l*** | synaptopodin 2-like |  |  |
| I | ASU_Acar_G.161 | 100553891 | ENSACAG00000003859 | ***tbx18*** | T-box 18 |  |  |
| I | ASU_Acar_G.2102 | 100553423 | - | ***tcf15*** | transcription factor 15 (basic helix-loop-helix) |  |  |
| I | ASU_Acar_G.18176 | - | - | ***tdrp*** | testis development related protein |  |  |
| I | ASU_Acar_G.19898 | 100553606 | ENSACAG00000014745 | ***tmod4*** | tropomodulin 4 (muscle) |  |  |
| I | ASU_Acar_G.2815 | 100551526 | ENSACAG00000006898 | ***tnnc1*** | troponin C type 1 (slow) |  |  |
| I | ASU_Acar_G.10863 | - | - | ***tnnc2*** | troponin C type 2 (fast) |  |  |
| I | ASU_Acar_G.6954 | 100565698 | ENSACAG00000003672 | ***tnni1*** | troponin I type 1 (skeletal, slow) |  |  |
| I | ASU_Acar_G.1753 | 100566786 | - | ***tnni2*** | troponin I type 2 (skeletal, fast) |  |  |
| I | ASU_Acar_G.16015 | 100558563 | ENSACAG00000016780 | ***tnnt1*** | troponin T type 1 (skeletal, slow) |  |  |
| I | ASU_Acar_G.4322 | 100563655 | ENSACAG00000011919 | ***trim7*** | tripartite motif containing 7 |  |  |
| I | ASU_Acar_G.13293 | 100556790 | ENSACAG00000006665 | ***trim72*** | tripartite motif containing 72 |  |  |
| I | ASU_Acar_G.12461 | 100567009 | ENSACAG00000004862 | ***txlnb*** | taxilin beta |  |  |
| I | ASU_Acar_G.2431 | 100564041 | ENSACAG00000014238 | ***vgll2*** | vestigial like 2 (Drosophila) |  |  |
| I | ASU_Acar_G.10333 | 100560977 | ENSACAG00000009771 | ***xirp1*** | xin actin-binding repeat containing 1 |  |  |
| II | ASU_Acar_G.3154 | 100557614 | ENSACAG00000001874 | ***abhd6*** | abhydrolase domain containing 6 |  |  |
| II | ASU_Acar_G.17467 | 100563884 | ENSACAG00000011428 | ***adam28*** | ADAM metallopeptidase domain 28 |  |  |
| II | ASU_Acar_G.3327 | 100566345 | ENSACAG00000012229 | ***aldh1a1*** | aldehyde dehydrogenase 1 family, member A1 |  |  |
| II | ASU_Acar_G.7675 | 100565498 | ENSACAG00000005112 | ***arhgap29*** | Rho GTPase activating protein 29 |  |  |
| II | ASU_Acar_G.4698 | - | ENSACAG00000009126 | ***aspn*** | asporin |  |  |
| II | ASU_Acar_G.17349 | 100564542 | ENSACAG00000003402 | ***atp1a1*** | ATPase, Na+/K+ transporting, alpha 1 polypeptide |  |  |
| II | ASU_Acar_G.4965 | 100556957 | ENSACAG00000005114 | ***atp1b3*** | ATPase, Na+/K+ transporting, beta 3 polypeptide |  |  |
| II | ASU_Acar_G.13090 | 100557828 | ENSACAG00000015968 | ***aurka*** | aurora kinase A |  |  |
| II | ASU_Acar_G.21113 | 100566199 | ENSACAG00000011399 | ***c15orf26*** | chromosome 15 open reading frame 26 |  |  |
| II | ASU_Acar_G.5514 | 100566994 | ENSACAG00000003249 | ***c21orf62*** | chromosome 21 open reading frame 62 |  |  |
| II | ASU_Acar_G.7191 | 100552716 | ENSACAG00000014147 | ***ca13*** | carbonic anhydrase XIII |  |  |
| II | ASU_Acar_G.8949 | 100555471 | ENSACAG00000013211 | ***cbfb*** | core-binding factor, beta subunit |  |  |
| II | ASU_Acar_G.22801 | 100567596 | ENSACAG00000015087 | ***cdh11*** | cadherin 11, type 2, OB-cadherin (osteoblast) |  |  |
| II | ASU_Acar_G.7506 | 100559528 | ENSACAG00000003458 | ***chi3l1*** | chitinase 3-like 1 (cartilage glycoprotein-39) |  |  |
| II | ASU_Acar_G.9161 | 100567920 | ENSACAG00000010501 | ***chic2*** | cysteine-rich hydrophobic domain 2 |  |  |
| II | ASU_Acar_G.11771 | - | ENSACAG00000026507 | ***chit1*** | chitinase 1 (chitotriosidase) |  |  |
| II | ASU_Acar_G.252 | 100556754 | ENSACAG00000008058 | ***clcf1*** | cardiotrophin-like cytokine factor 1 |  |  |
| II | ASU_Acar_G.5618 | 100556311 | ENSACAG00000010969 | ***col8a1*** | collagen, type VIII, alpha 1 |  |  |
| II | ASU_Acar_G.18314 | 100555626 | ENSACAG00000003633 | ***col8a2*** | collagen, type VIII, alpha 2 |  |  |
| II | ASU_Acar_G.13954 | 100567088 | ENSACAG00000011823 | ***cyp2j2*** | cytochrome P450 2J2 |  |  |
| II | ASU_Acar_G.9964 | 100564794 | ENSACAG00000008272 | ***ddc*** | dopa decarboxylase (aromatic L-amino acid decarboxylase) |  |  |
| II | ASU_Acar_G.3316 | 100565752 | ENSACAG00000002411 | ***dmrt2*** | dystrophia myotonica-protein kinase |  |  |
| II | ASU_Acar_G.8260 | 100559070 | ENSACAG00000003899 | ***ednra*** | endothelin receptor type A |  |  |
| II | ASU_Acar_G.3530 | 100558202 | ENSACAG00000013121 | ***efcc1*** | EF-hand and coiled-coil domain containing 1 |  |  |
| II | ASU_Acar_G.4253 | - | - | ***egln3*** | egl-9 family hypoxia-inducible factor 3 |  |  |
| II | ASU_Acar_G.9541 | 100559791 | ENSACAG00000002953 | ***enpp6*** | ectonucleotide pyrophosphatase/phosphodiesterase 6 |  |  |
| II | ASU_Acar_G.5801 | 100561163 | ENSACAG00000005061 | ***epha4*** | EPH receptor A4 |  |  |
| II | ASU_Acar_G.5876 | 100558795 | ENSACAG00000009269 | ***ets2*** | v-ets avian erythroblastosis virus E26 oncogene homolog 2 |  |  |
| II | ASU_Acar_G.3760 | 100566666 | ENSACAG00000007987 | ***f2r*** | coagulation factor II (thrombin) receptor |  |  |
| II | ASU_Acar_G.8614 | 100567527 | ENSACAG00000016612 | ***fam198b*** | family with sequence similarity 198, member B |  |  |
| II | ASU_Acar_G.3789 | 100566862 | ENSACAG00000015211 | ***fam19a1*** | family with sequence similarity 19 (chemokine (C-C motif)-like), member A1 |  |  |
| II | ASU_Acar_G.8031 | 100553768 | ENSACAG00000003723 | ***fam69c*** | family with sequence similarity 69, member C |  |  |
| II | ASU_Acar_G.14694 | - | - | **G.14694** | unknown protein-coding |  |  |
| II | ASU_Acar_G.21065 | - | ENSACAG00000025572 | ***ccer2*** | coiled-coil glutamate-rich protein 2 |  |  |
| II | ASU_Acar_G.2194 | - | - | **G.2194** | unknown protein-coding |  |  |
| II | ASU_Acar_G.4243 | 100551985 | ENSACAG00000003374 | ***glis3*** | GLIS family zinc finger 3 |  |  |
| II | ASU_Acar_G.9689 | 100567461 | ENSACAG00000016095 | ***glt8d2*** | glycosyltransferase 8 domain containing 2 |  |  |
| II | ASU_Acar_G.2955 | 100567843 | ENSACAG00000009147 | ***hexb*** | hexosaminidase B (beta polypeptide) |  |  |
| II | ASU_Acar_G.435 | 100560623 | ENSACAG00000002130 | ***hhipl2*** | HHIP-like 2 |  |  |
| II | ASU_Acar_G.10506 | 100568251 | ENSACAG00000016160 | ***igfbp4*** | insulin-like growth factor binding protein 4 |  |  |
| II | ASU_Acar_G.14777 | - | ENSACAG00000012548 | ***il17re*** | interleukin 17 receptor E |  |  |
| II | ASU_Acar_G.13871 | 100553004 | ENSACAG00000007454 | ***il1r1*** | interleukin 1 receptor, type I |  |  |
| II | ASU_Acar_G.10470 | 100556197 | ENSACAG00000009703 | ***itga9*** | integrin, alpha 9 |  |  |
| II | ASU_Acar_G.5228 | 100557485 | ENSACAG00000002586 | ***itm2c*** | integral membrane protein 2C |  |  |
| II | ASU_Acar_G.17978 | 100554317 | ENSACAG00000003744 | ***kcnc1*** | potassium voltage-gated channel, Shaw-related subfamily, member 1 |  |  |
| II | ASU_Acar_G.6144 | 100562076 | ENSACAG00000009168 | ***kcnj6*** | potassium inwardly-rectifying channel, subfamily J, member 6 |  |  |
| II | ASU_Acar_G.2066 | 100558835 | ENSACAG00000011013 | ***kif26a*** | kinesin family member 26A |  |  |
| II | ASU_Acar_G.15083 | 100553525 | ENSACAG00000011771 | ***kif26b*** | kinesin family member 26B |  |  |
| II | ASU_Acar_G.17584 | 100563687 | ENSACAG00000009954 | ***lama1*** | laminin, alpha 1 |  |  |
| II | ASU_Acar_G.7795 | 100567585 | ENSACAG00000003883 | ***lppr4*** | lipid phosphate phosphatase-related protein type 4 |  |  |
| II | ASU_Acar_G.22850 | 100562035 | ENSACAG00000004176 | ***lrfn3*** | leucine rich repeat and fibronectin type III domain containing 3 |  |  |
| II | ASU_Acar_G.19366 | 100553536 | - | ***ly6g6d*** | lymphocyte antigen 6 complex, locus G6D |  |  |
| II | ASU_Acar_G.1684 | 100559375 | ENSACAG00000008787 | ***lyve1*** | lymphatic vessel endothelial hyaluronan receptor 1 |  |  |
| II | ASU_Acar_G.141 | 100565875 | ENSACAG00000004140 | ***mdk*** | midkine (neurite growth-promoting factor 2) |  |  |
| II | ASU_Acar_G.8828 | - | - | ***mepe*** | matrix extracellular phosphoglycoprotein |  |  |
| II | ASU_Acar_G.3199 | 100557610 | ENSACAG00000017238 | ***mettl7a*** | methyltransferase like 7A |  |  |
| II | ASU_Acar_G.4065 | 100563065 | ENSACAG00000017614 | ***mllt3*** | myeloid/lymphoid or mixed-lineage leukemia (trithorax homolog, Drosophila); translocated to, 3 |  |  |
| II | ASU_Acar_G.9462 | 100566168 | ENSACAG00000012994 | ***ndnf*** | neuron-derived neurotrophic factor |  |  |
| II | ASU_Acar_G.9654 | 100551610 | ENSACAG00000012117 | ***ndst4*** | N-deacetylase/N-sulfotransferase (heparan glucosaminyl) 4 |  |  |
| II | ASU_Acar_G.8400 | 100556249 | ENSACAG00000002584 | ***nell2*** | NEL-like 2 (chicken) |  |  |
| II | ASU_Acar_G.9361 | 100555144 | ENSACAG00000013376 | ***npffr2*** | neuropeptide FF receptor 2 |  |  |
| II | ASU_Acar_G.10524 | 100563412 | ENSACAG00000000328 | ***nphp3*** | nephronophthisis 3 (adolescent) |  |  |
| II | ASU_Acar_G.7294 | 100562549 | ENSACAG00000003024 | ***olfm3*** | olfactomedin 3 |  |  |
| II | ASU_Acar_G.10755 | 100562562 | ENSACAG00000011889 | ***or5v1*** | olfactory receptor, family 5, subfamily V, member 1 |  |  |
| II | ASU_Acar_G.13725 | 100566043 | ENSACAG00000004288 | ***ovch2*** | ovochymase 2 (gene/pseudogene) |  |  |
| II | ASU_Acar_G.7466 | 100552921 | ENSACAG00000015801 | ***pacsin1*** | protein kinase C and casein kinase substrate in neurons 1 |  |  |
| II | ASU_Acar_G.5371 | 100558401 | - | ***pcp4*** | Purkinje cell protein 4 |  |  |
| II | ASU_Acar_G.2989 | 100565158 | ENSACAG00000012731 | ***pcsk1*** | proprotein convertase subtilisin/kexin type 1 |  |  |
| II | ASU_Acar_G.9594 | 100565834 | ENSACAG00000010297 | ***pdgfra*** | platelet-derived growth factor receptor, alpha polypeptide |  |  |
| II | ASU_Acar_G.5069 | 100555463 | ENSACAG00000015055 | ***phldb2*** | pleckstrin homology-like domain, family B, member 2 |  |  |
| II | ASU_Acar_G.5662 | 100555200 | ENSACAG00000012419 | ***pir*** | pirin (iron-binding nuclear protein) |  |  |
| II | ASU_Acar_G.12074 | 100558686 | ENSACAG00000003793 | ***prr16*** | proline rich 16 |  |  |
| II | ASU_Acar_G.624 | 100556951 | ENSACAG00000004285 | ***prss35*** | protease, serine, 35 |  |  |
| II | ASU_Acar_G.2923 | 100560825 | ENSACAG00000005636 | ***prune2*** | prune homolog 2 (Drosophila) |  |  |
| II | ASU_Acar_G.12146 | 100568055 | ENSACAG00000006527 | ***ptgfr*** | prostaglandin F receptor (FP) |  |  |
| II | ASU_Acar_G.7245 | 100568241 | ENSACAG00000000141 | ***ptpn22*** | protein tyrosine phosphatase, non-receptor type 22 (lymphoid) |  |  |
| II | ASU_Acar_G.5620 | 100557939 | ENSACAG00000006780 | ***ptx3*** | pentraxin 3, long |  |  |
| II | ASU_Acar_G.15277 | 100562835 | ENSACAG00000005848 | ***rasl11b*** | RAS-like, family 11, member B |  |  |
| II | ASU_Acar_G.13182 | 100555482 | ENSACAG00000007894 | ***rnase_x*** | one of many ribonuclease orthologs |  |  |
| II | ASU_Acar_G.13494 | 100567276 | ENSACAG00000004369 | ***robo2*** | roundabout, axon guidance receptor, homolog 2 (Drosophila) |  |  |
| II | ASU_Acar_G.3015 | 100554214 | ENSACAG00000000922 | ***ror2*** | receptor tyrosine kinase-like orphan receptor 2 |  |  |
| II | ASU_Acar_G.6566 | 100557619 | ENSACAG00000003098 | ***s1pr1*** | sphingosine-1-phosphate receptor 1 |  |  |
| II | ASU_Acar_G.13441 | 100559539 | ENSACAG00000014399 | ***sall1*** | sal-like 1 (Drosophila) |  |  |
| II | ASU_Acar_G.13416 | 100557376 | ENSACAG00000012748 | ***sall4*** | sal-like 4 (Drosophila) |  |  |
| II | ASU_Acar_G.8250 | 100557427 | ENSACAG00000015808 | ***scube1*** | signal peptide, CUB domain, EGF-like 1 |  |  |
| II | ASU_Acar_G.5977 | 100552120 | ENSACAG00000003815 | ***sema4g*** | sema domain, immunoglobulin domain (Ig), transmembrane domain (TM) and short cytoplasmic domain, (semaphorin) 4G |  |  |
| II | ASU_Acar_G.5074 | 100559583 | ENSACAG00000004328 | ***serpine2*** | serpin peptidase inhibitor, clade E (nexin, plasminogen activator inhibitor type 1), member 2 |  |  |
| II | ASU_Acar_G.13786 | 100557704 | - | ***sfxn3*** | sideroflexin 3 |  |  |
| II | ASU_Acar_G.4158 | 100557870 | ENSACAG00000017719 | ***sh3gl2*** | SH3-domain GRB2-like 2 |  |  |
| II | ASU_Acar_G.3420 | 100560033 | ENSACAG00000016539 | ***shc3*** | SHC (Src homology 2 domain containing) transforming protein 3 |  |  |
| II | ASU_Acar_G.890 | 100559511 | ENSACAG00000011354 | ***slc16a10*** | solute carrier family 16 (aromatic amino acid transporter), member 10 |  |  |
| II | ASU_Acar_G.10604 | 100562367 | ENSACAG00000011641 | ***slc27a2*** | solute carrier family 27 (fatty acid transporter), member 2 |  |  |
| II | ASU_Acar_G.2638 | 100555912 | ENSACAG00000009875 | ***slc6a11*** | solute carrier family 6 (neurotransmitter transporter), member 11 |  |  |
| II | ASU_Acar_G.22803 | 100557960 | ENSACAG00000016675 | ***slc6a2*** | solute carrier family 6 (neurotransmitter transporter), member 2 |  |  |
| II | ASU_Acar_G.12023 | 100559476 | ENSACAG00000023071 | ***sncaip*** | synuclein, alpha interacting protein |  |  |
| II | ASU_Acar_G.1659 | 100562794 | ENSACAG00000005431 | ***sntg2*** | syntrophin, gamma 2 |  |  |
| II | ASU_Acar_G.17778 | 100556664 | ENSACAG00000010309 | ***spon2*** | spondin 2, extracellular matrix protein |  |  |
| II | ASU_Acar_G.9105 | 100562556 | ENSACAG00000012670 | ***spp1*** | secreted phosphoprotein 1 |  |  |
| II | ASU_Acar_G.5738 | 100529108 | ENSACAG00000002478 | ***st6gal2*** | ST6 beta-galactosamide alpha-2,6-sialyltranferase 2 |  |  |
| II | ASU_Acar_G.2099 | 100562404 | ENSACAG00000004945 | ***synj2*** | synaptojanin 2 |  |  |
| II | ASU_Acar_G.6318 | 100555262 | ENSACAG00000015148 | ***tagln3*** | transgelin 3 |  |  |
| II | ASU_Acar_G.428 | - | ENSACAG00000010836 | ***tdrd15*** | tudor domain containing 15 |  |  |
| II | ASU_Acar_G.10734 | 100563348 | ENSACAG00000013101 | ***tmem98*** | transmembrane protein 98 |  |  |
| II | ASU_Acar_G.17203 | 100561272 | ENSACAG00000012237 | ***tnfrsf6b*** | tumor necrosis factor receptor superfamily, member 6b, decoy |  |  |
| II | ASU_Acar_G.2981 | 100561028 | ENSACAG00000008316 | ***traip*** | TRAF interacting protein |  |  |
| II | ASU_Acar_G.8605 | 100566683 | ENSACAG00000007164 | ***tspan8*** | tetraspanin 8 |  |  |
| II | ASU_Acar_G.3014 | 100561219 | ENSACAG00000011160 | ***vcan*** | versican |  |  |
| II | ASU_Acar_G.13650 | 100553589 | ENSACAG00000017769 | ***vwce*** | von Willebrand factor C and EGF domains |  |  |
| II | ASU_Acar_G.3955 | 100555653 | ENSACAG00000017453 | ***wnt5a*** | wingless-type MMTV integration site family, member 5A |  |  |
| II | ASU_Acar_G.6944 | 100555532 | ENSACAG00000013504 | ***znf704*** | zinc finger protein 704 |  |  |
