# Supplementary material for: Transcriptomic Analysis of Tail Regeneration in the Lizard Anolis carolinensis Reveals Activation of Conserved Vertebrate Developmental and Repair Mechanisms
Source: PLoS One. 2014 Aug 20;9(8):e105004. doi: 10.1371/journal.pone.0105004 (PMC4139331; doi:10.1371/journal.pone.0105004)
Supplement: Table S5 — GO Biological Process analysis (DAVID) on differentially expressed genes in the proximal regenerating tail (Cluster I). (DOCX) [file pone.0105004.s010.docx]

| **Table S5. GO Biological Process analysis (DAVID) on DE genes in the proximal regenerating tail (Cluster I).** | | | | | | | | | |  |
| --- | --- | --- | --- | --- | --- | --- | --- | --- | --- | --- |
| **Term** | **Description** | **Count** | **%** | **PValue** | **Genes** | **Fold Enrichment** | **Bonferroni** | **Benjamini** | **FDR** | |
| GO:0003012 | muscle system process | 32 | 18.29 | 1.65E-30 | MYBPC2, TNNC2, TNNC1, MYL3, MYBPC1, MYBPC3, MYL1, PGAM2, TRIM72, MYOT, DES, MYOM2, MYL6B, MYOM1, CHRNA1, SCN5A, DTNA, KCNMA1, ACTC1, ACTA1, MSTN, ACTN2, MYH6, TNNI2, TRDN, TNNT3, TNNT1, RYR1, STBD1, CHRNE, CASQ2, CHRNG | 18.27 | 1.52E-27 | 1.52E-27 | 2.59E-27 | |
| GO:0006936 | muscle contraction | 30 | 17.14 | 6.63E-29 | MYBPC2, TNNC2, TNNC1, MYL3, MYBPC1, MYBPC3, MYL1, PGAM2, MYOT, DES, MYOM2, MYL6B, MYOM1, CHRNA1, SCN5A, DTNA, KCNMA1, ACTC1, ACTA1, ACTN2, MYH6, TNNI2, TRDN, TNNT3, TNNT1, RYR1, STBD1, CHRNE, CASQ2, CHRNG | 18.81 | 6.07E-26 | 3.04E-26 | 1.04E-25 | |
| GO:0007517 | muscle organ development | 28 | 16.00 | 3.44E-22 | MEF2C, MYOD1, MYL2, TNNC1, MYL3, MYBPC3, MYL1, TRIM72, SPEG, MYL6B, PAX7, OBSL1, MKX, MKL2, CHRNA1, ACTC1, ACTA1, MSTN, MYLPF, MYH6, CSRP3, FLNB, MURC, NEB, XIRP1, ITGA7, VGLL2, TCF15 | 12.73 | 3.15E-19 | 1.05E-19 | 5.37E-19 | |
| GO:0006941 | striated muscle contraction | 15 | 8.57 | 1.48E-17 | ACTC1, TNNC2, TNNC1, MYL3, MYBPC3, MYL1, PGAM2, MYH6, TNNI2, TNNT3, TNNT1, MYOM1, CHRNA1, CASQ2, DTNA | 31.29 | 1.35E-14 | 3.38E-15 | 2.31E-14 | |
| GO:0014706 | striated muscle tissue development | 17 | 9.71 | 7.88E-14 | MYOD1, ACTC1, MYL2, ACTA1, TNNC1, MYL3, MYBPC3, MYLPF, MYH6, CSRP3, XIRP1, MYL6B, PAX7, VGLL2, OBSL1, MKL2, CHRNA1 | 13.71 | 7.22E-11 | 1.44E-11 | 1.23E-10 | |
| GO:0060537 | muscle tissue development | 17 | 9.71 | 1.73E-13 | MYOD1, ACTC1, MYL2, ACTA1, TNNC1, MYL3, MYBPC3, MYLPF, MYH6, CSRP3, XIRP1, MYL6B, PAX7, VGLL2, OBSL1, MKL2, CHRNA1 | 13.05 | 1.59E-10 | 2.64E-11 | 2.71E-10 | |
| GO:0006937 | regulation of muscle contraction | 11 | 6.29 | 3.17E-09 | KCNMA1, TNNT3, TNNT1, MYL2, TNNC2, MYL3, TNNC1, MYBPC3, ATP2A1, TNNI1, TPM3 | 14.66 | 2.90E-06 | 4.14E-07 | 4.95E-06 | |
| GO:0007507 | heart development | 16 | 9.14 | 6.07E-09 | ACTC1, MYL2, ALPK3, TNNC1, MYL3, MYBPC3, PDLIM3, COL2A1, SMYD1, MYH6, CSRP3, XIRP1, OBSL1, MKL2, CASQ2, NFATC1 | 7.14 | 5.56E-06 | 6.95E-07 | 9.48E-06 | |
| GO:0048738 | cardiac muscle tissue development | 10 | 5.71 | 6.21E-09 | ACTC1, XIRP1, MYL2, MYL3, TNNC1, MYBPC3, OBSL1, MYH6, MKL2, CSRP3 | 16.83 | 5.68E-06 | 6.32E-07 | 9.69E-06 | |
| GO:0044057 | regulation of system process | 18 | 10.29 | 2.00E-08 | KCNMA1, MYL2, TNNC2, TNNC1, MYL3, MYBPC3, MSTN, MYH6, CSRP3, TNNI1, TPM3, TNNT3, DES, TNNT1, ATP2A1, CELF2, SCN5A, CAMK2A | 5.59 | 1.83E-05 | 1.83E-06 | 3.12E-05 | |
| GO:0003009 | skeletal muscle contraction | 6 | 3.43 | 8.26E-08 | TNNT3, TNNT1, TNNC2, TNNC1, CHRNA1, TNNI2 | 47.97 | 7.56E-05 | 6.88E-06 | 1.29E-04 | |
| GO:0031032 | actomyosin structure organization | 7 | 4.00 | 3.45E-07 | ACTC1, ACTA1, XIRP1, MYL2, LIMCH1, OBSL1, MYH6 | 23.99 | 3.16E-04 | 2.63E-05 | 5.38E-04 | |
| GO:0060538 | skeletal muscle organ development | 9 | 5.14 | 3.73E-07 | MYOD1, ACTA1, MYL3, MYL6B, PAX7, MYLPF, VGLL2, CHRNA1, CSRP3 | 13.08 | 3.41E-04 | 2.63E-05 | 5.82E-04 | |
| GO:0007519 | skeletal muscle tissue development | 9 | 5.14 | 3.73E-07 | MYOD1, ACTA1, MYL3, MYL6B, PAX7, MYLPF, VGLL2, CHRNA1, CSRP3 | 13.08 | 3.41E-04 | 2.63E-05 | 5.82E-04 | |
| GO:0042692 | muscle cell differentiation | 11 | 6.29 | 4.86E-07 | MYOD1, ACTC1, ACTA1, XIRP1, MYL2, SPEG, LGALS1, OBSL1, MYH6, MKL2, CHRNA1 | 8.72 | 4.45E-04 | 3.18E-05 | 7.59E-04 | |
| GO:0050881 | musculoskeletal movement | 6 | 3.43 | 1.14E-06 | TNNT3, TNNT1, TNNC2, TNNC1, CHRNA1, TNNI2 | 30.30 | 0.001 | 6.98E-05 | 0.002 | |
| GO:0050879 | multicellular organismal movement | 6 | 3.43 | 1.14E-06 | TNNT3, TNNT1, TNNC2, TNNC1, CHRNA1, TNNI2 | 30.30 | 0.001 | 6.98E-05 | 0.002 | |
| GO:0030029 | actin filament-based process | 14 | 8.00 | 1.28E-06 | ACTC1, TNXB, MYL2, ACTA1, MYL1, PDLIM3, MYH6, GAS7, FLNB, XIRP1, XIRP2, MYL6B, LIMCH1, OBSL1 | 5.57 | 0.001 | 7.30E-05 | 0.002 | |
| GO:0030239 | myofibril assembly | 6 | 3.43 | 1.97E-06 | ACTC1, ACTA1, XIRP1, MYL2, OBSL1, MYH6 | 27.41 | 0.002 | 1.06E-04 | 0.003 | |
| GO:0006942 | regulation of striated muscle contraction | 6 | 3.43 | 2.53E-06 | TNNT3, MYL2, MYL3, MYBPC3, ATP2A1, TNNI1 | 26.17 | 0.002 | 1.29E-04 | 0.004 | |
| GO:0060047 | heart contraction | 6 | 3.43 | 3.20E-06 | ACTC1, MYL2, MYL3, TNNC1, MYBPC3, MYL1 | 25.03 | 0.003 | 1.54E-04 | 0.005 | |
| GO:0003015 | heart process | 6 | 3.43 | 3.20E-06 | ACTC1, MYL2, MYL3, TNNC1, MYBPC3, MYL1 | 25.03 | 0.003 | 1.54E-04 | 0.005 | |
| GO:0055008 | cardiac muscle tissue morphogenesis | 6 | 3.43 | 7.43E-06 | ACTC1, MYL2, MYL3, TNNC1, MYBPC3, MYH6 | 21.32 | 0.007 | 3.40E-04 | 0.012 | |
| GO:0060415 | muscle tissue morphogenesis | 6 | 3.43 | 7.43E-06 | ACTC1, MYL2, MYL3, TNNC1, MYBPC3, MYH6 | 21.32 | 0.007 | 3.40E-04 | 0.012 | |
| GO:0003007 | heart morphogenesis | 8 | 4.57 | 1.01E-05 | ACTC1, MYL2, MYL3, TNNC1, MYBPC3, COL2A1, MYH6, MKL2 | 10.51 | 0.009 | 4.40E-04 | 0.016 | |
| GO:0051216 | cartilage development | 8 | 4.57 | 1.10E-05 | BMP3, COL9A1, LECT1, PAX7, ACAN, MGP, COL2A1, COL11A2 | 10.37 | 0.010 | 4.60E-04 | 0.017 | |
| GO:0007010 | cytoskeleton organization | 17 | 9.71 | 1.13E-05 | ABLIM2, ACTC1, TNXB, MYL2, ACTA1, SPTBN4, PDLIM3, MYH6, GAS7, FLNB, DES, XIRP1, XIRP2, LIMCH1, OBSL1, SYNM, KLHL20 | 3.74 | 0.010 | 4.48E-04 | 0.018 | |
| GO:0055002 | striated muscle cell development | 7 | 4.00 | 1.52E-05 | ACTC1, ACTA1, XIRP1, MYL2, OBSL1, MYH6, CHRNA1 | 12.92 | 0.014 | 5.79E-04 | 0.024 | |
| GO:0043462 | regulation of ATPase activity | 5 | 2.86 | 1.82E-05 | TNNT3, MYL3, TNNC1, MYBPC3, MYH6 | 29.98 | 0.016 | 6.65E-04 | 0.028 | |
| GO:0030036 | actin cytoskeleton organization | 12 | 6.86 | 2.27E-05 | ACTC1, TNXB, ACTA1, XIRP1, MYL2, XIRP2, LIMCH1, PDLIM3, OBSL1, MYH6, GAS7, FLNB | 5.09 | 0.021 | 8.01E-04 | 0.036 | |
| GO:0055001 | muscle cell development | 7 | 4.00 | 2.34E-05 | ACTC1, ACTA1, XIRP1, MYL2, OBSL1, MYH6, CHRNA1 | 11.99 | 0.021 | 7.94E-04 | 0.037 | |
| GO:0010927 | cellular component assembly involved in morphogenesis | 6 | 3.43 | 3.22E-05 | ACTC1, ACTA1, XIRP1, MYL2, OBSL1, MYH6 | 15.99 | 0.029 | 0.001 | 0.050 | |
| GO:0007155 | cell adhesion | 21 | 12.00 | 3.41E-05 | HAPLN1, TNXB, MYBPC2, CLSTN2, EGFL6, LPP, MYBPC1, COL22A1, MYBPC3, COL28A1, MGP, ACTN2, COL2A1, ACTN3, ECM2, COL9A1, ITGA7, ACAN, SUSD5, COL11A2, THBS4 | 2.88 | 0.031 | 0.001 | 0.053 | |
| GO:0051146 | striated muscle cell differentiation | 8 | 4.57 | 3.45E-05 | MYOD1, ACTC1, ACTA1, XIRP1, MYL2, OBSL1, MYH6, CHRNA1 | 8.72 | 0.031 | 0.001 | 0.054 | |
| GO:0022610 | biological adhesion | 21 | 12.00 | 3.48E-05 | HAPLN1, TNXB, MYBPC2, CLSTN2, EGFL6, LPP, MYBPC1, COL22A1, MYBPC3, COL28A1, MGP, ACTN2, COL2A1, ACTN3, ECM2, COL9A1, ITGA7, ACAN, SUSD5, COL11A2, THBS4 | 2.87 | 0.031 | 0.001 | 0.054 | |
| GO:0060048 | cardiac muscle contraction | 5 | 2.86 | 3.77E-05 | ACTC1, MYL3, TNNC1, MYBPC3, MYL1 | 25.25 | 0.034 | 0.001 | 0.059 | |
| GO:0055010 | ventricular cardiac muscle morphogenesis | 5 | 2.86 | 6.95E-05 | MYL2, MYL3, TNNC1, MYBPC3, MYH6 | 21.81 | 0.062 | 0.002 | 0.109 | |
| GO:0055013 | cardiac muscle cell development | 4 | 2.29 | 1.68E-04 | ACTC1, XIRP1, MYL2, OBSL1 | 34.89 | 0.143 | 0.005 | 0.263 | |
| GO:0055006 | cardiac cell development | 4 | 2.29 | 1.68E-04 | ACTC1, XIRP1, MYL2, OBSL1 | 34.89 | 0.143 | 0.005 | 0.263 | |
| GO:0001501 | skeletal system development | 12 | 6.86 | 4.79E-04 | BMP3, COL9A1, COL9A2, TBX15, LECT1, CLEC3A, PAX7, ACAN, MGP, COL2A1, COL11A2, TCF15 | 3.61 | 0.355 | 0.012 | 0.746 | |
| GO:0008015 | blood circulation | 9 | 5.14 | 7.00E-04 | KCNMA1, ACTC1, MYL2, MYL3, TNNC1, MYBPC3, MYL1, MYH6, SCN5A | 4.64 | 0.474 | 0.018 | 1.089 | |
| GO:0003013 | circulatory system process | 9 | 5.14 | 7.00E-04 | KCNMA1, ACTC1, MYL2, MYL3, TNNC1, MYBPC3, MYL1, MYH6, SCN5A | 4.64 | 0.474 | 0.018 | 1.089 | |
| GO:0030198 | extracellular matrix organization | 7 | 4.00 | 7.29E-04 | CSGALNACT1, TNXB, ADAMTS20, ACAN, COL2A1, COL11A2, ECM2 | 6.46 | 0.487 | 0.018 | 1.133 | |
| GO:0002062 | chondrocyte differentiation | 4 | 2.29 | 7.90E-04 | COL9A1, ACAN, COL2A1, COL11A2 | 21.32 | 0.515 | 0.019 | 1.227 | |
| GO:0008016 | regulation of heart contraction | 6 | 3.43 | 0.001 | DES, MYL3, CELF2, MYH6, CSRP3, SCN5A | 7.48 | 0.668 | 0.028 | 1.861 | |
| GO:0043062 | extracellular structure organization | 8 | 4.57 | 0.002 | CSGALNACT1, TNXB, ADAMTS20, ACAN, COL2A1, CHRNA1, COL11A2, ECM2 | 4.71 | 0.753 | 0.034 | 2.356 | |
| GO:0055003 | cardiac myofibril assembly | 3 | 1.71 | 0.002 | ACTC1, MYL2, OBSL1 | 47.97 | 0.759 | 0.034 | 2.398 | |
| GO:0055007 | cardiac muscle cell differentiation | 4 | 2.29 | 0.002 | ACTC1, XIRP1, MYL2, OBSL1 | 16.69 | 0.780 | 0.035 | 2.548 | |
| GO:0030048 | actin filament-based movement | 4 | 2.29 | 0.002 | ACTC1, MYL6B, MYL1, MYH6 | 16.69 | 0.780 | 0.035 | 2.548 | |
| GO:0035051 | cardiac cell differentiation | 4 | 2.29 | 0.003 | ACTC1, XIRP1, MYL2, OBSL1 | 13.71 | 0.933 | 0.061 | 4.496 | |
| GO:0006816 | calcium ion transport | 7 | 4.00 | 0.004 | CACNA2D1, JPH2, ATP2A1, RYR1, CACNB1, CAMK2A, NFATC1 | 4.73 | 0.963 | 0.072 | 5.453 | |
| GO:0033275 | actin-myosin filament sliding | 3 | 1.71 | 0.004 | MYL6B, MYL1, MYH6 | 31.98 | 0.965 | 0.072 | 5.553 | |
| GO:0070252 | actin-mediated cell contraction | 3 | 1.71 | 0.004 | MYL6B, MYL1, MYH6 | 31.98 | 0.965 | 0.072 | 5.553 | |
| GO:0030049 | muscle filament sliding | 3 | 1.71 | 0.004 | MYL6B, MYL1, MYH6 | 31.98 | 0.965 | 0.072 | 5.553 | |
| GO:0006873 | cellular ion homeostasis | 11 | 6.29 | 0.006 | KCNMA1, JPH2, XIRP1, PYGM, ATP2A1, RYR1, CHRNA1, CHRNE, CSRP3, SYPL2, CHRNG | 2.82 | 0.994 | 0.104 | 8.268 | |
| GO:0048705 | skeletal system morphogenesis | 6 | 3.43 | 0.006 | TBX15, ACAN, MGP, COL2A1, COL11A2, TCF15 | 5.14 | 0.996 | 0.113 | 9.147 | |
| GO:0055082 | cellular chemical homeostasis | 11 | 6.29 | 0.006 | KCNMA1, JPH2, XIRP1, PYGM, ATP2A1, RYR1, CHRNA1, CHRNE, CSRP3, SYPL2, CHRNG | 2.78 | 0.996 | 0.111 | 9.178 | |
| GO:0045932 | negative regulation of muscle contraction | 3 | 1.71 | 0.007 | KCNMA1, TNNT1, ATP2A1 | 23.99 | 0.998 | 0.116 | 9.767 | |
| GO:0032989 | cellular component morphogenesis | 11 | 6.29 | 0.008 | ACTC1, ACTA1, XIRP1, MYL2, SPTBN4, OBSL1, MYH6, UNC5C, GAS7, SLIT2, NFATC1 | 2.66 | 0.999 | 0.141 | 12.145 | |
| GO:0006029 | proteoglycan metabolic process | 4 | 2.29 | 0.010 | CSGALNACT1, LECT1, ACAN, COL2A1 | 8.92 | 1.000 | 0.164 | 14.392 | |
| GO:0015674 | di-, tri-valent inorganic cation transport | 7 | 4.00 | 0.010 | CACNA2D1, JPH2, ATP2A1, RYR1, CACNB1, CAMK2A, NFATC1 | 3.82 | 1.000 | 0.162 | 14.482 | |
| GO:0050801 | ion homeostasis | 11 | 6.29 | 0.010 | KCNMA1, JPH2, XIRP1, PYGM, ATP2A1, RYR1, CHRNA1, CHRNE, CSRP3, SYPL2, CHRNG | 2.58 | 1.000 | 0.160 | 14.602 | |
| GO:0048729 | tissue morphogenesis | 7 | 4.00 | 0.011 | ACTC1, MYL2, MYL3, TNNC1, MYBPC3, MYH6, SLIT2 | 3.73 | 1.000 | 0.172 | 15.936 | |
| GO:0002026 | regulation of the force of heart contraction | 3 | 1.71 | 0.012 | MYL3, MYH6, CSRP3 | 17.99 | 1.000 | 0.177 | 16.669 | |
| GO:0006811 | ion transport | 16 | 9.14 | 0.013 | KCNMA1, CACNA2D1, JPH2, SLC25A4, ATP1B4, CACNB1, COL9A1, ATP2A1, SLC25A22, RYR1, CHRNA1, SCN5A, CAMK2A, CHRNE, CHRNG, NFATC1 | 2.00 | 1.000 | 0.195 | 18.750 | |
| GO:0001502 | cartilage condensation | 3 | 1.71 | 0.016 | ACAN, MGP, COL2A1 | 15.15 | 1.000 | 0.231 | 22.527 | |
| GO:0006875 | cellular metal ion homeostasis | 7 | 4.00 | 0.016 | KCNMA1, JPH2, PYGM, ATP2A1, RYR1, CSRP3, SYPL2 | 3.43 | 1.000 | 0.228 | 22.571 | |
| GO:0030705 | cytoskeleton-dependent intracellular transport | 4 | 2.29 | 0.017 | ACTC1, MYL6B, MYL1, MYH6 | 7.38 | 1.000 | 0.229 | 22.976 | |
| GO:0048878 | chemical homeostasis | 12 | 6.86 | 0.017 | KCNMA1, SOAT2, JPH2, XIRP1, PYGM, ATP2A1, RYR1, CHRNA1, CHRNE, CSRP3, SYPL2, CHRNG | 2.25 | 1.000 | 0.234 | 23.865 | |
| GO:0055065 | metal ion homeostasis | 7 | 4.00 | 0.020 | KCNMA1, JPH2, PYGM, ATP2A1, RYR1, CSRP3, SYPL2 | 3.28 | 1.000 | 0.259 | 26.836 | |
| GO:0019725 | cellular homeostasis | 11 | 6.29 | 0.023 | KCNMA1, JPH2, XIRP1, PYGM, ATP2A1, RYR1, CHRNA1, CHRNE, CSRP3, SYPL2, CHRNG | 2.26 | 1.000 | 0.290 | 30.425 | |
| GO:0042592 | homeostatic process | 15 | 8.57 | 0.024 | KCNMA1, SOAT2, JPH2, COL2A1, CSRP3, SYPL2, COL9A1, PYGM, XIRP1, ATP2A1, RYR1, COL11A2, CHRNA1, CHRNE, CHRNG | 1.92 | 1.000 | 0.293 | 31.106 | |
| GO:0032971 | regulation of muscle filament sliding | 2 | 1.14 | 0.031 | TNNC1, MYBPC3 | 63.96 | 1.000 | 0.360 | 38.591 | |
| GO:0030199 | collagen fibril organization | 3 | 1.71 | 0.036 | ACAN, COL2A1, COL11A2 | 9.93 | 1.000 | 0.403 | 43.587 | |
| GO:0007015 | actin filament organization | 4 | 2.29 | 0.039 | ACTC1, ACTA1, PDLIM3, GAS7 | 5.33 | 1.000 | 0.421 | 45.900 | |
| GO:0043501 | skeletal muscle adaptation | 2 | 1.14 | 0.041 | ACTA1, MSTN | 47.97 | 1.000 | 0.434 | 47.804 | |
| GO:0006874 | cellular calcium ion homeostasis | 6 | 3.43 | 0.042 | JPH2, PYGM, ATP2A1, RYR1, CSRP3, SYPL2 | 3.15 | 1.000 | 0.435 | 48.474 | |
| GO:0055074 | calcium ion homeostasis | 6 | 3.43 | 0.046 | JPH2, PYGM, ATP2A1, RYR1, CSRP3, SYPL2 | 3.06 | 1.000 | 0.463 | 51.886 | |
| GO:0030003 | cellular cation homeostasis | 7 | 4.00 | 0.049 | KCNMA1, JPH2, PYGM, ATP2A1, RYR1, CSRP3, SYPL2 | 2.64 | 1.000 | 0.479 | 54.051 | |
