# Supplementary material for: Transcriptomic Analysis of Tail Regeneration in the Lizard Anolis carolinensis Reveals Activation of Conserved Vertebrate Developmental and Repair Mechanisms
Source: PLoS One. 2014 Aug 20;9(8):e105004. doi: 10.1371/journal.pone.0105004 (PMC4139331; doi:10.1371/journal.pone.0105004)
Supplement: Table S6 — GO Biological Process analysis (DAVID) on differentially expressed genes in the regenerating tail tip (Cluster II). (DOCX) [file pone.0105004.s011.docx]

**Table S6. GO Biological Process analysis (DAVID) on DE genes in the regenerating tail tip (Cluster II).**

| **Term** | **Description** | **Count** | | **%** | | **PValue** | **Genes** | **Fold Enrichment** | **Bonferroni** | **Benjamini** | **FDR** |
| --- | --- | --- | --- | --- | --- | --- | --- | --- | --- | --- | --- |
| GO:0001501 | skeletal system development | | 9 | | 9.28 | 5.81E-04 | WNT5A, TNFRSF11B, PDGFRA, ROR2, MEPE, CBFB, IGFBP4, SPP1, TWIST1 | 4.71 | 0.44 | 0.44 | 0.91 |
| GO:0035295 | tube development | | 7 | | 7.22 | 0.002 | WNT5A, EDNRA, FGFR4, SALL1, PDGFRA, PTK7, TWIST1 | 5.31 | 0.85 | 0.61 | 2.97 |
| GO:0051050 | positive regulation of transport | | 7 | | 7.22 | 0.002 | EDNRA, EDN3, PCSK1, RAB8B, PTX3, F2R, THY1 | 5.24 | 0.87 | 0.49 | 3.17 |
| GO:0009611 | response to wounding | | 10 | | 10.31 | 0.004 | PCSK1, SCUBE1, PDGFRA, PLA2G7, ENTPD1, PTX3, MDK, IGFBP4, F2R, SPP1 | 3.15 | 0.98 | 0.63 | 6.09 |
| GO:0032844 | regulation of homeostatic process | | 5 | | 5.15 | 0.005 | EDNRA, TNFRSF11B, F2R, SPP1, THY1 | 7.33 | 0.99 | 0.60 | 7.05 |
| GO:0048545 | response to steroid hormone stimulus | | 6 | | 6.19 | 0.006 | PCSK1, KRT19, TNFRSF11B, TH, PDGFRA, SPP1 | 5.22 | 1.00 | 0.60 | 8.43 |
| GO:0009725 | response to hormone stimulus | | 8 | | 8.25 | 0.006 | CGA, PCSK1, KRT19, TNFRSF11B, BSG, TH, PDGFRA, SPP1 | 3.64 | 1.00 | 0.57 | 8.98 |
| GO:0007223 | Wnt receptor signaling pathway, calcium modulating pathway | | 3 | | 3.09 | 0.007 | WNT5A, WNT16, ROR2 | 23.86 | 1.00 | 0.57 | 10.12 |
| GO:0016055 | Wnt receptor signaling pathway | | 5 | | 5.15 | 0.008 | DKK2, WNT5A, WNT16, ROR2, WIF1 | 6.28 | 1.00 | 0.58 | 11.82 |
| GO:0048598 | embryonic morphogenesis | | 7 | | 7.22 | 0.010 | WNT5A, SALL4, TH, PTK7, ROR2, TWIST1, PTPRQ | 3.81 | 1.00 | 0.61 | 14.11 |
| GO:0010033 | response to organic substance | | 11 | | 11.34 | 0.010 | EDNRA, CGA, PCSK1, KRT19, IL1R1, TNFRSF11B, BSG, TH, PDGFRA, F2R, SPP1 | 2.55 | 1.00 | 0.59 | 14.38 |
| GO:0009719 | response to endogenous stimulus | | 8 | | 8.25 | 0.010 | CGA, PCSK1, KRT19, TNFRSF11B, BSG, TH, PDGFRA, SPP1 | 3.30 | 1.00 | 0.56 | 14.62 |
| GO:0007166 | cell surface receptor linked signal transduction | | 20 | | 20.62 | 0.011 | WNT5A, CGA, EDN3, FGFR4, IL1R1, WNT16, GPR158, BSG, MAML2, PTPN22, THY1, DKK2, EDNRA, OR5V1, PDGFRA, ROR2, WIF1, PDGFC, ENTPD1, F2R | 1.80 | 1.00 | 0.55 | 15.46 |
| GO:0051928 | positive regulation of calcium ion transport | | 3 | | 3.09 | 0.016 | EDNRA, F2R, THY1 | 15.18 | 1.00 | 0.68 | 22.71 |
| GO:0032846 | positive regulation of homeostatic process | | 3 | | 3.09 | 0.023 | EDNRA, SPP1, THY1 | 12.53 | 1.00 | 0.79 | 31.06 |
| GO:0007267 | cell-cell signaling | | 9 | | 9.28 | 0.025 | WNT5A, EDN3, CGA, PCSK1, WNT16, FGFR4, SALL1, TH, SLC1A1 | 2.51 | 1.00 | 0.79 | 33.09 |
| GO:0043270 | positive regulation of ion transport | | 3 | | 3.09 | 0.026 | EDNRA, F2R, THY1 | 11.93 | 1.00 | 0.78 | 33.49 |
| GO:0006590 | thyroid hormone generation | | 2 | | 2.06 | 0.035 | CGA, DIO2 | 55.67 | 1.00 | 0.86 | 42.97 |
| GO:0051173 | positive regulation of nitrogen compound metabolic process | | 9 | | 9.28 | 0.036 | GLIS3, SALL4, MAML2, PDGFRA, PDGFC, RUNX1, PTX3, CBFB, F2R | 2.33 | 1.00 | 0.85 | 44.05 |
| GO:0006032 | chitin catabolic process | | 2 | | 2.06 | 0.041 | CHI3L1, CHIT1 | 47.72 | 1.00 | 0.87 | 48.06 |
| GO:0006030 | chitin metabolic process | | 2 | | 2.06 | 0.041 | CHI3L1, CHIT1 | 47.72 | 1.00 | 0.87 | 48.06 |
| GO:0006954 | inflammatory response | | 6 | | 6.19 | 0.043 | SCUBE1, PLA2G7, PTX3, IGFBP4, F2R, SPP1 | 3.08 | 1.00 | 0.88 | 50.24 |
| GO:0043009 | chordate embryonic development | | 6 | | 6.19 | 0.046 | EDNRA, SALL4, PDGFRA, PTK7, ROR2, TWIST1 | 3.03 | 1.00 | 0.88 | 52.58 |
| GO:0009792 | embryonic development ending in birth or egg hatching | | 6 | | 6.19 | 0.048 | EDNRA, SALL4, PDGFRA, PTK7, ROR2, TWIST1 | 3.00 | 1.00 | 0.88 | 53.74 |
| GO:0033674 | positive regulation of kinase activity | | 5 | | 5.15 | 0.048 | EDNRA, EDN3, CDC42, PDGFC, F2R | 3.61 | 1.00 | 0.87 | 53.92 |
| GO:0031328 | positive regulation of cellular biosynthetic process | | 9 | | 9.28 | 0.049 | GLIS3, SALL4, MAML2, PDGFRA, PDGFC, RUNX1, PTX3, CBFB, F2R | 2.19 | 1.00 | 0.86 | 54.73 |
| GO:0051281 | positive regulation of release of sequestered calcium ion into cytosol | | 2 | | 2.06 | 0.052 | EDNRA, THY1 | 37.11 | 1.00 | 0.87 | 56.93 |
| GO:0014829 | vascular smooth muscle contraction | | 2 | | 2.06 | 0.052 | EDNRA, EDN3 | 37.11 | 1.00 | 0.87 | 56.93 |
| GO:0009891 | positive regulation of biosynthetic process | | 9 | | 9.28 | 0.053 | GLIS3, SALL4, MAML2, PDGFRA, PDGFC, RUNX1, PTX3, CBFB, F2R | 2.16 | 1.00 | 0.86 | 57.31 |
| GO:0051347 | positive regulation of transferase activity | | 5 | | 5.15 | 0.054 | EDNRA, EDN3, CDC42, PDGFC, F2R | 3.48 | 1.00 | 0.86 | 58.17 |
| GO:0007155 | cell adhesion | | 9 | | 9.28 | 0.054 | CLDN17, CDH9, NELL2, PTK7, ROR2, SPON2, ENTPD1, SPP1, THY1 | 2.15 | 1.00 | 0.85 | 58.60 |
| GO:0022610 | biological adhesion | | 9 | | 9.28 | 0.055 | CLDN17, CDH9, NELL2, PTK7, ROR2, SPON2, ENTPD1, SPP1, THY1 | 2.14 | 1.00 | 0.84 | 58.85 |
| GO:0010817 | regulation of hormone levels | | 4 | | 4.12 | 0.060 | EDN3, CGA, PCSK1, DIO2 | 4.42 | 1.00 | 0.86 | 62.42 |
| GO:0043549 | regulation of kinase activity | | 6 | | 6.19 | 0.060 | EDNRA, EDN3, CDC42, PDGFC, F2R, THY1 | 2.81 | 1.00 | 0.85 | 62.42 |
| GO:0006796 | phosphate metabolic process | | 11 | | 11.34 | 0.060 | GMFB, FGFR4, LPPR4, MYO3A, PDGFRA, PTK7, PTPN22, ROR2, STK17A, F2R, PTPRQ | 1.89 | 1.00 | 0.84 | 62.45 |
| GO:0006793 | phosphorus metabolic process | | 11 | | 11.34 | 0.060 | GMFB, FGFR4, LPPR4, MYO3A, PDGFRA, PTK7, PTPN22, ROR2, STK17A, F2R, PTPRQ | 1.89 | 1.00 | 0.84 | 62.45 |
| GO:0051924 | regulation of calcium ion transport | | 3 | | 3.09 | 0.063 | EDNRA, F2R, THY1 | 7.26 | 1.00 | 0.85 | 64.07 |
| GO:0035270 | endocrine system development | | 3 | | 3.09 | 0.063 | CGA, PCSK1, MDK | 7.26 | 1.00 | 0.85 | 64.07 |
| GO:0042423 | catecholamine biosynthetic process | | 2 | | 2.06 | 0.063 | DDC, TH | 30.37 | 1.00 | 0.84 | 64.29 |
| GO:0032101 | regulation of response to external stimulus | | 4 | | 4.12 | 0.068 | EDNRA, EDN3, F2R, SPP1 | 4.20 | 1.00 | 0.86 | 67.09 |
| GO:0042403 | thyroid hormone metabolic process | | 2 | | 2.06 | 0.069 | CGA, DIO2 | 27.84 | 1.00 | 0.85 | 67.48 |
| GO:0051482 | elevation of cytosolic calcium ion concentration during G-protein signaling, coupled to IP3 second messenger (phospholipase C activating) | | 2 | | 2.06 | 0.069 | EDNRA, F2R | 27.84 | 1.00 | 0.85 | 67.48 |
| GO:0051338 | regulation of transferase activity | | 6 | | 6.19 | 0.069 | EDNRA, EDN3, CDC42, PDGFC, F2R, THY1 | 2.69 | 1.00 | 0.84 | 67.73 |
| GO:0003006 | reproductive developmental process | | 5 | | 5.15 | 0.070 | CGA, BSG, PDGFRA, ROR2, SPP1 | 3.19 | 1.00 | 0.84 | 67.93 |
| GO:0046697 | decidualization | | 2 | | 2.06 | 0.074 | BSG, SPP1 | 25.69 | 1.00 | 0.85 | 70.39 |
| GO:0045935 | positive regulation of nucleobase, nucleoside, nucleotide and nucleic acid metabolic process | | 8 | | 8.25 | 0.076 | GLIS3, SALL4, MAML2, PDGFRA, PDGFC, RUNX1, CBFB, F2R | 2.14 | 1.00 | 0.85 | 71.04 |
| GO:0030334 | regulation of cell migration | | 4 | | 4.12 | 0.078 | EDN3, PDGFRA, F2R, THY1 | 3.95 | 1.00 | 0.85 | 72.45 |
| GO:0034097 | response to cytokine stimulus | | 3 | | 3.09 | 0.079 | PCSK1, IL1R1, PDGFRA | 6.34 | 1.00 | 0.85 | 72.93 |
| GO:0010959 | regulation of metal ion transport | | 3 | | 3.09 | 0.083 | EDNRA, F2R, THY1 | 6.19 | 1.00 | 0.86 | 74.50 |
| GO:0001893 | maternal placenta development | | 2 | | 2.06 | 0.085 | BSG, SPP1 | 22.27 | 1.00 | 0.86 | 75.44 |
| GO:0046850 | regulation of bone remodeling | | 2 | | 2.06 | 0.085 | TNFRSF11B, SPP1 | 22.27 | 1.00 | 0.86 | 75.44 |
| GO:0035088 | establishment or maintenance of apical/basal cell polarity | | 2 | | 2.06 | 0.085 | CDC42, PTK7 | 22.27 | 1.00 | 0.86 | 75.44 |
| GO:0051279 | regulation of release of sequestered calcium ion into cytosol | | 2 | | 2.06 | 0.085 | EDNRA, THY1 | 22.27 | 1.00 | 0.86 | 75.44 |
| GO:0045124 | regulation of bone resorption | | 2 | | 2.06 | 0.085 | TNFRSF11B, SPP1 | 22.27 | 1.00 | 0.86 | 75.44 |
| GO:0001775 | cell activation | | 5 | | 5.15 | 0.090 | PDGFRA, PTPN22, ENTPD1, CBFB, F2R | 2.91 | 1.00 | 0.87 | 77.45 |
| GO:0043278 | response to morphine | | 2 | | 2.06 | 0.091 | EDNRA, PCSK1 | 20.88 | 1.00 | 0.86 | 77.64 |
| GO:0014072 | response to isoquinoline alkaloid | | 2 | | 2.06 | 0.091 | EDNRA, PCSK1 | 20.88 | 1.00 | 0.86 | 77.64 |
| GO:0010557 | positive regulation of macromolecule biosynthetic process | | 8 | | 8.25 | 0.092 | GLIS3, SALL4, MAML2, PDGFRA, PDGFC, RUNX1, CBFB, F2R | 2.04 | 1.00 | 0.86 | 78.01 |
| GO:0035113 | embryonic appendage morphogenesis | | 3 | | 3.09 | 0.094 | WNT5A, SALL4, TWIST1 | 5.76 | 1.00 | 0.86 | 78.82 |
| GO:0030326 | embryonic limb morphogenesis | | 3 | | 3.09 | 0.094 | WNT5A, SALL4, TWIST1 | 5.76 | 1.00 | 0.86 | 78.82 |
| GO:0048015 | phosphoinositide-mediated signaling | | 3 | | 3.09 | 0.096 | EDNRA, EDN3, F2R | 5.69 | 1.00 | 0.86 | 79.48 |
| GO:0070555 | response to interleukin-1 | | 2 | | 2.06 | 0.096 | PCSK1, IL1R1 | 19.65 | 1.00 | 0.86 | 79.64 |
| GO:0042310 | vasoconstriction | | 2 | | 2.06 | 0.096 | EDNRA, EDN3 | 19.65 | 1.00 | 0.86 | 79.64 |
| GO:0030335 | positive regulation of cell migration | | 3 | | 3.09 | 0.097 | EDN3, PDGFRA, F2R | 5.63 | 1.00 | 0.86 | 80.13 |
| GO:0031344 | regulation of cell projection organization | | 3 | | 3.09 | 0.097 | CDC42, SPP1, THY1 | 5.63 | 1.00 | 0.86 | 80.13 |
| GO:0006468 | protein amino acid phosphorylation | | 8 | | 8.25 | 0.099 | GMFB, FGFR4, MYO3A, PDGFRA, PTK7, ROR2, STK17A, F2R | 2.00 | 1.00 | 0.86 | 80.70 |
