# Supplementary material for: Transcriptomic Analysis of Tail Regeneration in the Lizard Anolis carolinensis Reveals Activation of Conserved Vertebrate Developmental and Repair Mechanisms
Source: PLoS One. 2014 Aug 20;9(8):e105004. doi: 10.1371/journal.pone.0105004 (PMC4139331; doi:10.1371/journal.pone.0105004)
Supplement: Table S7 — KEGG pathway analysis (DAVID) on differentially expressed genes in the 25 dpa regenerating tail. (DOCX) [file pone.0105004.s012.docx]

**Table S7. KEGG pathway analysis (DAVID) on DE genes in the 25 dpa regenerating tail.**

| **Term** | **Term Description** | **Count** | **%** | **PValue** | **Genes** | **Fold Enrichment** | | **Bonferroni** | **Benjamini** | **FDR** |
| --- | --- | --- | --- | --- | --- | --- | --- | --- | --- | --- |
| hsa05410 | Hypertrophic cardiomyopathy (HCM) | 12 | 4.43 | 5.29E-08 | ACTC1, CACNA2D1, DES, MYL2, MYL3, TNNC1, MYBPC3, ITGA7, CACNB1, MYH6, PRKAA2, TPM3 | | 8.97 | 4.71E-06 | 4.71E-06 | 5.74E-05 |
| hsa04510 | Focal adhesion | 16 | 5.90 | 3.08E-07 | TNXB, MYL2, MYLPF, ACTN2, MYL10, COL2A1, ACTN3, FLNC, FLNB, CDC42, ITGA7, PDGFRA, PDGFC, COL11A2, SPP1, THBS4 | | 5.06 | 2.74E-05 | 1.37E-05 | 3.34E-04 |
| hsa05414 | Dilated cardiomyopathy | 11 | 4.06 | 1.18E-06 | ACTC1, CACNA2D1, DES, MYL2, MYL3, TNNC1, MYBPC3, ITGA7, CACNB1, MYH6, TPM3 | | 7.60 | 1.05E-04 | 3.51E-05 | 0.00 |
| hsa04260 | Cardiac muscle contraction | 9 | 3.32 | 2.31E-05 | ACTC1, CACNA2D1, MYL2, MYL3, TNNC1, ATP1B4, CACNB1, MYH6, TPM3 | | 7.33 | 0.00 | 5.14E-04 | 0.03 |
| hsa04810 | Regulation of actin cytoskeleton | 13 | 4.80 | 1.00E-04 | FGFR4, MYL2, MYLPF, ACTN2, MYL10, FGF13, ACTN3, CDC42, CFL2, ITGA7, PDGFRA, PDGFC, F2R | | 3.84 | 0.01 | 0.00 | 0.11 |
| hsa04512 | ECM-receptor interaction | 7 | 2.58 | 0.002 | TNXB, ITGA7, SV2B, COL2A1, COL11A2, THBS4, SPP1 | | 5.30 | 0.15 | 0.03 | 1.94 |
| hsa04670 | Leukocyte transendothelial migration | 8 | 2.95 | 0.002 | CLDN17, CDC42, MYL2, MYLPF, MYL10, ACTN2, ACTN3, THY1 | | 4.31 | 0.18 | 0.03 | 2.37 |
| hsa04530 | Tight junction | 8 | 2.95 | 0.005 | CLDN17, CDC42, MYL2, MYLPF, MYL10, ACTN2, MYH6, ACTN3 | | 3.79 | 0.33 | 0.05 | 4.79 |
| hsa04020 | Calcium signaling pathway | 9 | 3.32 | 0.006 | EDNRA, SLC25A4, TNNC2, TNNC1, ATP2A1, PDGFRA, RYR1, CAMK2A, F2R | | 3.25 | 0.39 | 0.05 | 5.94 |
| hsa05412 | Arrhythmogenic right ventricular cardiomyopathy (ARVC) | 6 | 2.21 | 0.006 | CACNA2D1, DES, ITGA7, CACNB1, ACTN2, ACTN3 | | 5.02 | 0.43 | 0.05 | 6.56 |
| hsa04310 | Wnt signaling pathway | 8 | 2.95 | 0.009 | DKK2, WNT5A, WNT16, SFRP2, WIF1, FZD4, CAMK2A, NFATC1 | | 3.37 | 0.54 | 0.07 | 8.94 |
| hsa04010 | MAPK signaling pathway | 10 | 3.69 | 0.022 | MEF2C, CDC42, FGFR4, IL1R1, CACNA2D1, PDGFRA, CACNB1, FGF13, FLNC, FLNB | | 2.38 | 0.86 | 0.15 | 21.19 |
| hsa04360 | Axon guidance | 6 | 2.21 | 0.049 | ABLIM2, CDC42, CFL2, UNC5C, SLIT2, NFATC1 | | 2.96 | 0.99 | 0.29 | 42.24 |
