# Supplementary material for: Transcriptomic Analysis of Tail Regeneration in the Lizard Anolis carolinensis Reveals Activation of Conserved Vertebrate Developmental and Repair Mechanisms
Source: PLoS One. 2014 Aug 20;9(8):e105004. doi: 10.1371/journal.pone.0105004 (PMC4139331; doi:10.1371/journal.pone.0105004)
Supplement: Table S8 — Differentially expressed genes elevated (10-fold) in the regenerating tip compared to embryo and satellite cells. (DOCX) [file pone.0105004.s013.docx]

| **Table S8. DE genes elevated (10-fold) in the regenerating tip compared to embryo and satellite cells.** | | | | |
| --- | --- | --- | --- | --- |
| **Gene** | **NCBI_ID** | **ensembl_ID** | **Ortholog** | **Orthologous Gene Description** |
| ASU_Acar_G.20843 | 100558828 | - | ***aadacl_x*** | arylacetamide deacetylase-like 3 or 4 |
| ASU_Acar_G.20840 | 100559422 | ENSACAG00000002416 | ***aadacl4*** | arylacetamide deacetylase-like 4 |
| ASU_Acar_G.17179 | 100557840 | ENSACAG00000005188 | ***aloxe3*** | arachidonate lipoxygenase 3 |
| ASU_Acar_G.753 | 100559032 | ENSACAG00000010962 | ***aspg*** | asparaginase homolog (S. cerevisiae) |
| ASU_Acar_G.7506 | 100559528 | ENSACAG00000003458 | ***chi3l1*** | chitinase 3-like 1 (cartilage glycoprotein-39) |
| ASU_Acar_G.11771 | - | ENSACAG00000026507 | ***chit1*** | chitinase 1 (chitotriosidase) |
| ASU_Acar_G.5366 | 100556182 | ENSACAG00000021113 | ***cldn17*** | claudin 17 |
| ASU_Acar_G.15269 | 100563233 | ENSACAG00000006661 | ***corin*** | corin, serine peptidase |
| ASU_Acar_G.9653 | 100558940 | - | ***cyp2d14*** | cytochrome P450 2D14 |
| ASU_Acar_G.2039 | 100553484 | ENSACAG00000017365 | ***dio2*** | deiodinase, iodothyronine, type II |
| ASU_Acar_G.16937 | 100557586 | ENSACAG00000008330 | ***dkk2*** | dickkopf 2 homolog (Xenopus laevis) |
| ASU_Acar_G.12104 | 100552794 | ENSACAG00000005782 | ***dnase2b*** | deoxyribonuclease II beta |
| ASU_Acar_G.8260 | 100559070 | ENSACAG00000003899 | ***ednra*** | endothelin receptor type A |
| ASU_Acar_G.19198 | - | - | **G.19198** | unknown protein-coding |
| ASU_Acar_G.10506 | 100568251 | ENSACAG00000016160 | ***igfbp4*** | insulin-like growth factor binding protein 4 |
| ASU_Acar_G.22991 | 100563808 | ENSACAG00000017868 | ***krt19*** | keratin 19 |
| ASU_Acar_G.22997 | 100553851 | - | ***krt42*** | keratin 42 |
| ASU_Acar_G.16728 | 100359375 | ENSACAG00000027236 | ***li-ac-12*** | Keratin-associated beta-protein 12 |
| ASU_Acar_G.16727 | 100359377 | ENSACAG00000007609 | ***li-ac-14*** | Keratin-associated beta-protein 14 |
| ASU_Acar_G.16695 | 100379173 | ENSACAG00000025169 | ***li-ac-17*** | Keratin-associated beta-protein 17 |
| ASU_Acar_G.16706 | 100554184 | ENSACAG00000007588 | ***li-ac-5*** | Keratin-associated beta-protein 5 |
| ASU_Acar_G.16692 | 100555160 | - | ***li-ac-x*** | Keratin-associated beta-protein X |
| ASU_Acar_G.16694 | 100558368 | ENSACAG00000007612 | ***li-ac-x*** | Keratin-associated beta-protein X |
| ASU_Acar_G.16714 | 100557315 | ENSACAG00000007603 | ***li-ac-x*** | Keratin-associated beta-protein X |
| ASU_Acar_G.16722 | 100558565 | ENSACAG00000007613 | ***li-ac-x*** | Keratin-associated beta-protein X |
| ASU_Acar_G.15467 | 100562102 | ENSACAG00000003555 | ***lypd6b*** | LY6/PLAUR domain containing 6B |
| ASU_Acar_G.16715 | 100559746 | - | ***maml2*** | mastermind-like protein 2-like |
| ASU_Acar_G.8828 | - | - | ***mepe*** | extracelular matrix phosphoglycoprotein |
| ASU_Acar_G.9462 | 100566168 | ENSACAG00000012994 | ***ndnf*** | neuron-derived neurotrophic factor |
| ASU_Acar_G.5371 | 100558401 | - | ***pcp4*** | Purkinje cell protein 4 |
| ASU_Acar_G.9193 | 100564195 | - | ***plac8*** | placenta-specific gene 8 protein |
| ASU_Acar_G.19453 | 100567421 | - | ***ptprq*** | phosphotidylinositol phosphatase ptprq |
| ASU_Acar_G.15277 | 100562835 | ENSACAG00000005848 | ***rasl11b*** | RAS-like, family 11, member B |
| ASU_Acar_G.13182 | 100555482 | ENSACAG00000007894 | ***rnase_x*** | one of many ribonuclease orthologs |
| ASU_Acar_G.13441 | 100559539 | ENSACAG00000014399 | ***sall1*** | sal-like 1 (Drosophila) |
| ASU_Acar_G.19817 | 100557132 | - | ***sdr16c5*** | short chain dehydrogenase/reductase family 16C, member 5 |
| ASU_Acar_G.10785 | 100553546 | ENSACAG00000008817 | ***selenbp1*** | selenium binding protein 1 |
| ASU_Acar_G.19037 | 100562580 | ENSACAG00000008067 | ***selenbp1*** | selenium binding protein 1 |
| ASU_Acar_G.14005 | 100556598 | ENSACAG00000017642 | ***serpinb_x*** | serpin peptidase inhibitor, clade B (ovalbumin), member X |
| ASU_Acar_G.17778 | 100556664 | ENSACAG00000010309 | ***spon2*** | spondin 2, extracellular matrix protein |
| ASU_Acar_G.9105 | 100562556 | ENSACAG00000012670 | ***spp1*** | secreted phosphoprotein 1 |
| ASU_Acar_G.22339 | 100557208 | ENSACAG00000007487 | ***thy1*** | Thy-1 cell surface antigen |
| ASU_Acar_G.10108 | 100565708 | ENSACAG00000013144 | ***tmprss11b*** | transmembrane protease, serine 11B |
| ASU_Acar_G.9450 | 100553645 | ENSACAG00000006979 | ***wnt16*** | wingless-type MMTV integration site family, member 16 |
