# Supplementary material for: Transcriptomic Analysis of Tail Regeneration in the Lizard Anolis carolinensis Reveals Activation of Conserved Vertebrate Developmental and Repair Mechanisms
Source: PLoS One. 2014 Aug 20;9(8):e105004. doi: 10.1371/journal.pone.0105004 (PMC4139331; doi:10.1371/journal.pone.0105004)
Supplement: Table S9 — Differentially genes elevated (10-fold) in the proximal regenerating tail compared to embryo and satellite cells. (DOCX) [file pone.0105004.s014.docx]

| **Table S9. DE genes elevated (10-fold) in the proximal regenerating tail compared to embryo and satellite cells.** | | | | |
| --- | --- | --- | --- | --- |
| **Gene** | **NCBI_ID** | **ensembl_ID** | **Ortholog** | **Orthologous Gene Description** |
| ASU_Acar_G.21712 | 100554326 | ENSACAG00000013375 | ***acan*** | aggrecan |
| ASU_Acar_G.1152 | 100566789 | ENSACAG00000000836 | ***acta1*** | actin, alpha 1, skeletal muscle |
| ASU_Acar_G.859 | 100560364 | ENSACAG00000001104 | ***actc1*** | actin, alpha, cardiac muscle 1 |
| ASU_Acar_G.1793 | 100556098 | ENSACAG00000002769 | ***actn2*** | actinin, alpha 2 |
| ASU_Acar_G.12270 | 100557764 | ENSACAG00000017722 | ***adamts15*** | ADAM metallopeptidase with thrombospondin type 1 motif, 15 |
| ASU_Acar_G.21279 | 100557723 | ENSACAG00000003749 | ***atp1b4*** | ATPase, Na+/K+ transporting, beta 4 polypeptide |
| ASU_Acar_G.15160 | 100554310 | ENSACAG00000005552 | ***atp2a1*** | ATPase, Ca++ transporting, cardiac muscle, fast twitch 1 |
| ASU_Acar_G.8629 | 100563219 | ENSACAG00000010162 | ***bmp3*** | bone morphogenetic protein 3 |
| ASU_Acar_G.5693 | - | ENSACAG00000006295 | ***c2orf82*** | chromosome 2 open reading frame 82 |
| ASU_Acar_G.7058 | 100558336 | ENSACAG00000024792 | ***ca3*** | carbonic anhydrase III, muscle specific |
| ASU_Acar_G.8645 | 100566428 | ENSACAG00000012663 | ***cacna2d1*** | calcium channel, voltage-dependent, alpha 2/delta subunit 1 |
| ASU_Acar_G.21481 | 100554653 | ENSACAG00000015755 | ***casq1*** | casq1 |
| ASU_Acar_G.4130 | 100566014 | ENSACAG00000003187 | ***casq2*** | calsequestrin 2 (cardiac muscle) |
| ASU_Acar_G.22561 | 100562721 | ENSACAG00000005396 | ***ckm*** | creatine kinase, muscle |
| ASU_Acar_G.17769 | 100555752 | ENSACAG00000010347 | ***clec3a*** | C-type lectin domain family 3, member A |
| ASU_Acar_G.20941 | 100557792 | ENSACAG00000003040 | ***col11a2*** | collagen, type XI, alpha 2 |
| ASU_Acar_G.7464 | 100558670 | ENSACAG00000012191 | ***col22a1*** | collagen, type XXII, alpha 1 |
| ASU_Acar_G.10144 | 100562364 | ENSACAG00000013078 | ***col28a1*** | collagen, type XXVIII, alpha 1 |
| ASU_Acar_G.18867 | 100553016 | ENSACAG00000006064 | ***col2a1*** | collagen, type II, alpha 1 |
| ASU_Acar_G.987 | 100567245 | ENSACAG00000008314 | ***col9a1*** | collagen, type IX, alpha 1 |
| ASU_Acar_G.18455 | 100565206 | ENSACAG00000004683 | ***col9a2*** | collagen, type IX, alpha 2 |
| ASU_Acar_G.7008 | 100554037 | ENSACAG00000005213 | ***col9a3*** | collagen, type IX, alpha 3 |
| ASU_Acar_G.9893 | 100560783 | ENSACAG00000009025 | ***csgalnact1*** | chondroitin sulfate N-acetylgalactosaminyltransferase 1 |
| ASU_Acar_G.22949 | 100556256 | ENSACAG00000013760 | ***cyp2f3*** | cytochrome P450 2F3 |
| ASU_Acar_G.4806 | 100556174 | ENSACAG00000016930 | ***dhrs7c*** | dehydrogenase/reductase (SDR family) member 7C |
| ASU_Acar_G.2919 | - | ENSACAG00000009767 | ***ecm2*** | extracellular matrix protein 2 |
| ASU_Acar_G.6665 | 100561171 | ENSACAG00000006007 | ***eef1a2*** | eukaryotic translation elongation factor 1 alpha 2 |
| ASU_Acar_G.9223 | 100560848 | ENSACAG00000012287 | ***epyc*** | epiphycan |
| ASU_Acar_G.7172 | 100565108 | ENSACAG00000002165 | ***fmod*** | fibromodulin |
| ASU_Acar_G.15880 | - | - | **G.15880** | unknown protein-coding with DUF4585 domain |
| ASU_Acar_G.19355 | 100554070 | - | **G.19355** | mir-324 |
| ASU_Acar_G.4168 | - | - | **G.4168** | rna-directed dna polymerase from mobile element jockey-like |
| ASU_Acar_G.4437 | 100555782 | ENSACAG00000017003 | ***gas7*** | growth arrest-specific 7 |
| ASU_Acar_G.3693 | 100555587 | ENSACAG00000011432 | ***hapln1*** | hyaluronan and proteoglycan link protein 1 |
| ASU_Acar_G.10140 | 100564922 | ENSACAG00000006760 | ***hhatl*** | hedgehog acyltransferase-like |
| ASU_Acar_G.16488 | 100563683 | ENSACAG00000017205 | ***hsd17b14*** | 17-beta-hydroxysteroid dehydrogenase 14 |
| ASU_Acar_G.1581 | 100551911 | ENSACAG00000008404 | ***klhl31*** | kelch-like family member 31 |
| ASU_Acar_G.12414 | 100560271 | ENSACAG00000014492 | ***ldb3*** | LIM domain binding 3 |
| ASU_Acar_G.6073 | 100567514 | ENSACAG00000000442 | ***lect1*** | leukocyte cell derived chemotaxin 1 |
| ASU_Acar_G.9579 | 100553447 | ENSACAG00000012474 | ***lgals1*** | lectin, galactoside-binding, soluble, 1 |
| ASU_Acar_G.16693 | 100379172 | ENSACAG00000024814 | ***li-ac-27*** | Keratin-associated beta-protein 27 |
| ASU_Acar_G.2472 | 100566155 | ENSACAG00000015388 | ***lmod3*** | leiomodin 3 (fetal) |
| ASU_Acar_G.22910 | 100564202 | ENSACAG00000003759 | ***ltbp4*** | latent transforming growth factor beta binding protein 4 |
| ASU_Acar_G.19146 | 100559813 | ENSACAG00000000957 | ***matn1*** | matrilin 1, cartilage matrix protein |
| ASU_Acar_G.8050 | 100560579 | ENSACAG00000012284 | ***matn4*** | matrilin 4 |
| ASU_Acar_G.5161 | 100553237 | ENSACAG00000002751 | ***mettl21e*** | Protein-lysine methyltransferase |
| ASU_Acar_G.10180 | 100563668 | ENSACAG00000007829 | ***mkx*** | mohawk homeobox |
| ASU_Acar_G.2697 | 100557219 | ENSACAG00000004208 | ***mstn*** | myostatin |
| ASU_Acar_G.9162 | 100560974 | ENSACAG00000016657 | ***mybpc1*** | myosin binding protein C, slow type |
| ASU_Acar_G.10232 | 100558218 | ENSACAG00000012342 | ***mybpc2*** | myosin binding protein C, fast type |
| ASU_Acar_G.387 | 100558249 | ENSACAG00000010643 | ***myl1*** | myosin, light chain 1, alkali; skeletal, fast |
| ASU_Acar_G.17474 | 100566191 | ENSACAG00000002200 | ***myl2*** | myosin, light chain 2, regulatory, cardiac, slow |
| ASU_Acar_G.17789 | 100557845 | ENSACAG00000005125 | ***myl3*** | myosin, light chain 3, alkali; ventricular, skeletal, slow |
| ASU_Acar_G.3409 | 100561943 | ENSACAG00000022592 | ***myl6b*** | myosin, light chain 6B, alkali, smooth muscle and non-muscle |
| ASU_Acar_G.18654 | 100559161 | ENSACAG00000010684 | ***mylpf*** | myosin light chain, phosphorylatable, fast skeletal muscle |
| ASU_Acar_G.1040 | 100564167 | ENSACAG00000004962 | ***myom2*** | myomesin 2 |
| ASU_Acar_G.18467 | 100563435 | ENSACAG00000005357 | ***myom3*** | myomesin 3 |
| ASU_Acar_G.9302 | 100567139 | ENSACAG00000013422 | ***myoz2*** | myozenin 2 |
| ASU_Acar_G.13031 | 100564401 | ENSACAG00000010443 | ***mypn*** | myopalladin |
| ASU_Acar_G.15473 | 100563097 | ENSACAG00000003769 | ***neb*** | nebulin |
| ASU_Acar_G.6606 | 100565300 | ENSACAG00000000941 | ***nfatc1*** | nuclear factor of activated T-cells, cytoplasmic, calcineurin-dependent 1 |
| ASU_Acar_G.12694 | 100561114 | ENSACAG00000010135 | ***nrap*** | nebulin-related anchoring protein |
| ASU_Acar_G.10284 | 100566306 | ENSACAG00000006865 | ***obscn*** | obscurin, cytoskeletal calmodulin and titin-interacting RhoGEF |
| ASU_Acar_G.13772 | 100567602 | ENSACAG00000002010 | ***pgam2*** | phosphoglycerate mutase 2 (muscle) |
| ASU_Acar_G.22394 | 100566844 | ENSACAG00000012295 | ***pvalb*** | parvalbumin beta |
| ASU_Acar_G.22145 | 100553610 | ENSACAG00000005131 | ***pygm*** | phosphorylase, glycogen, muscle |
| ASU_Acar_G.19788 | 100551835 | ENSACAG00000008872 | ***rpl3l*** | ribosomal protein L3-like |
| ASU_Acar_G.11790 | - | - | ***ryr1*** | ryanodine receptor 1-like (skeletal) (predicted) |
| ASU_Acar_G.17542 | 100558429 | ENSACAG00000001965 | ***ryr1*** | ryanodine receptor 1 |
| ASU_Acar_G.15641 | 100552019 | ENSACAG00000024865 | ***serpinb2*** | serpin peptidase inhibitor, clade B (ovalbumin), member 2 |
| ASU_Acar_G.2023 | 100563775 | ENSACAG00000003213 | ***smoc2*** | SPARC related modular calcium binding 2 |
| ASU_Acar_G.4165 | 100567184 | ENSACAG00000024787 | ***soat2*** | sterol O-acyltransferase 2 |
| ASU_Acar_G.1782 | 100562065 | ENSACAG00000013938 | ***speg*** | SPEG complex locus |
| ASU_Acar_G.20936 | 100555634 | ENSACAG00000016380 | ***srl*** | sarcalumenin |
| ASU_Acar_G.12202 | 100567988 | ENSACAG00000009895 | ***synpo2l*** | synaptopodin 2-like |
| ASU_Acar_G.2102 | 100553423 | - | ***tcf15*** | transcription factor 15-like |
| ASU_Acar_G.6954 | 100565698 | ENSACAG00000003672 | ***tnni1*** | troponin I type 1 (skeletal, slow) |
| ASU_Acar_G.1753 | 100566786 | - | ***tnni2*** | troponin I type 2 (skeletal, fast) |
| ASU_Acar_G.16015 | 100558563 | ENSACAG00000016780 | ***tnnt1*** | troponin T type 1 (skeletal, slow) |
| ASU_Acar_G.1632 | 100566207 | ENSACAG00000009622 | ***tnnt3*** | troponin T type 3 (skeletal, fast) |
| ASU_Acar_G.356 | 100568083 | ENSACAG00000001672 | ***trdn*** | triadin |
| ASU_Acar_G.13293 | 100556790 | ENSACAG00000006665 | ***trim72*** | tripartite motif containing 72 |
| ASU_Acar_G.20995 | 100566067 | ENSACAG00000009001 | ***try_x*** | one of many trypsin orthologs |
| ASU_Acar_G.6121 | - | ENSACAG00000008325 | ***tspear*** | thrombospondin-type laminin G domain and EAR repeats |
| ASU_Acar_G.10736 | 100563155 | ENSACAG00000013446 | ***vwde*** | von Willebrand factor D and EGF domain-containing protein-like |
| ASU_Acar_G.13007 | 100559876 | ENSACAG00000000350 | ***xirp2*** | xin actin-binding repeat containing 2 |
|  |  |  |  |  |
|  |  |  |  |  |
|  |  |  |  |  |
|  |  |  |  |  |
